# Chemical Diversity from a Chinese Marine Red Alga, *Symphyocladia latiuscula*

**DOI:** 10.3390/md15120374

**Published:** 2017-12-01

**Authors:** Xiuli Xu, Haijin Yang, Zeinab G. Khalil, Liyuan Yin, Xue Xiao, Pratik Neupane, Paul V. Bernhardt, Angela A. Salim, Fuhang Song, Robert J. Capon

**Affiliations:** 1 School of Ocean Sciences, China University of Geosciences, Beijing 100083, China; xuxl@cugb.edu.cn (X.X.); yanghaijin52@163.com (H.Y.); yinliyuan999@sina.com (L.Y.); 2 CAS Key Laboratory of Pathogenic Microbiology and Immunology, Institute of Microbiology, Chinese Academy of Sciences, Beijing 100101, China; 3 Institute for Molecular Bioscience, The University of Queensland, Brisbane, QLD 4072, Australia; z.khalil@uq.edu.au (Z.G.K.); x.xue@uq.edu.au (X.X.); p.neupane@imb.uq.edu.au (P.N.); a.salim@uq.edu.au (A.A.S.); 4 School of Chemistry and Molecular Biosciences, The University of Queensland, Brisbane, QLD 4072, Australia; p.bernhardt@uq.edu.au

**Keywords:** marine red alga, *Symphyocladia latiuscula*, bromophenols, symphyocladins, aconitates

## Abstract

This study describes an investigation into secondary metabolites that are produced by a marine red alga, *Symphyocladia latiuscula*, which was collected from coastal waters off Qingdao, China. A combination of normal, reversed phase, and gel chromatography was used to isolate six citric acid derived natural products, aconitates A–F (**1**–**6**), together with two known and ten new polybrominated phenols, symphyocladins C/D (**7a/b**), and symphyocladins H–Q (**8a/b**, **9a/b** and **10**–**15**), respectively. Structure elucidation was achieved by detailed spectroscopic (including X-ray crystallographic) analysis. We propose a plausible and convergent biosynthetic pathway involving a key quinone methide intermediate, linking aconitates and symphyocladins.

## 1. Introduction

Historically, natural products have inspired the development of many pharmaceuticals and agrochemicals, which, have in turn, played an important role in improving human and animal health and agricultural productivity, enhancing the quality of life for communities across the globe [1]. One of the defining characteristics of natural products is their structure diversity, which can encompass complex carbocyclic and heterocyclic scaffolds, annotated with a wide array of functional groups and stereochemical features. As such, even a limited set of biosynthetic precursors can deliver remarkable chemical diversity. Illustrative of this phenomenon are bromophenols from marine red algae (Rhodophyta) [2,3,4,5,6,7,8,9,10,11,12,13,14,15,16,17,18,19,20]. For example, the red alga *Symphyocladia latiuscula* (Harvey) Yamada has been reported to produce a diverse array of bromophenols elaborated by sulfoxides, sulphones, sulfates, glutamines, pyrrolidin-2-ones, ureas, diketopiperazines, and aconitic acids, with biological properties spanning antibacterial [11,12], antifungal [10,11,12,13,14], antiviral [15], anticancer [16], free radical scavenging [9,17,18], aldose reductase inhibitory [19], and Taq DNA polymerase inhibitory activities [20]. *S. latiuscula* bromophenols typically contain at least one 2,3,6-tribromo-4,5-dihydroxybenzyl moiety, as consistent with a highly conserved biosynthetic pathway. This report described our efforts to further elaborate the chemical diversity of *S. latiuscula*.

## 2. Results and Discussion

**Figure 1:**
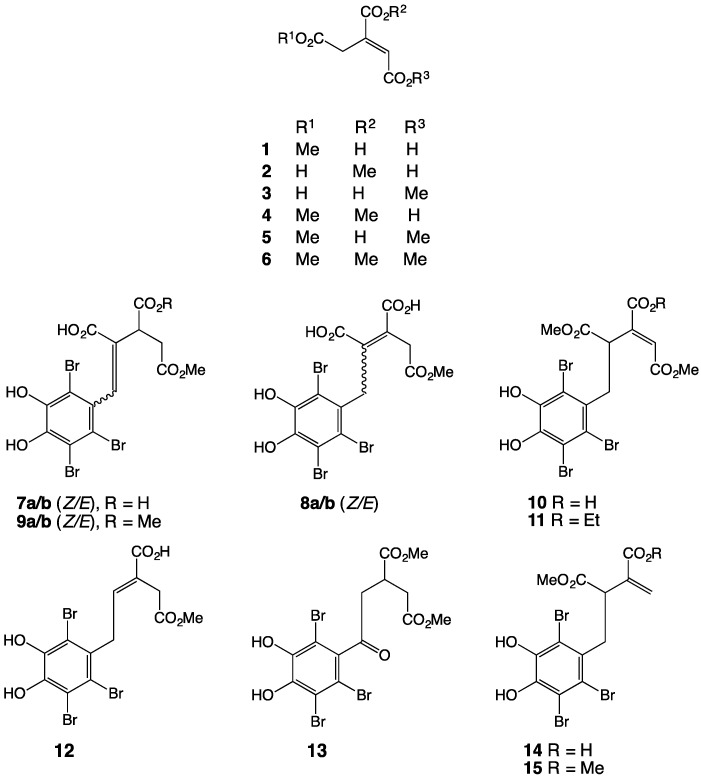
*S. latiuscula* metabolites **1**–**15**.

The EtOAc extract of a Chinese collection of *S. latiuscula* was concentrated *in vacuo* and subjected to a sequence of normal, reversed phase, and gel chromatography, with HPLC-MS analysis being used to prioritize fractions of interest. Following this strategy, we isolated and characterized six citric acid derived natural products, aconitates A–F (**1**–**6**), together with two known and ten new polybrominated phenol adducts, symphyocladins C/D (**7a/b**), and symphyocladins H–Q (**8a/b**, **9a/b** and **10**–**15**), respectively (Figure 1). A spectroscopic analysis approach (see Table 1, Table 2, Table 3, Table 4 and Table 5) to the structure elucidation of all of these metabolites is summarized below.

**Figure 2:**
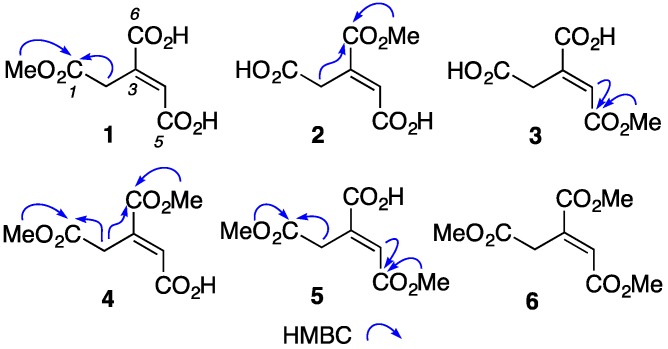
Diagnostic 2D NMR (methanol-*d*_4_) correlations for aconitates A–F (**1**–**6**).

HRESI(+)MS measurements confirmed that **1** (C_7_H_8_O_6_, Δmmu +0.2), **2** (C_7_H_8_O_6_, Δmmu +0.1) and **3** (C_7_H_8_O_6_, Δmmu +0.1) were isomeric, while analysis of the one-dimensional (1D) and two-dimensional (2D) NMR (methanol-*d*_4_) data (Figures S8–S13, Tables S2 and S3) suggested they were mono methyl esters of *E*-aconitic acid, for which we attribute the trivial names aconitates A–C. Assignment of *E* ∆^3,4^ configurations were inferred from diagnostic chemical shifts for H-4 and C-2 in **1** (δ_H_ 6.92; δ_C_ 33.8), **2** (δ_H_ 6.89; δ_C_ 33.9), and **3** (δ_H_ 6.91; δ_C_ 33.9), when compared to the authentic standards for *E* (δ_H_ 6.90; δ_C_ 33.8) and *Z* (δ_H_ 6.26; δ_C_ 40.2) aconitic acid (Figures S1–S7, Table S1). HMBC correlations permitted assignment of the methyl ester regiochemistry across **1**–**3** with correlations from (i) the OMe (δ_H_ 3.67) and H_2_-2 (δ_H_ 3.89) to C-1 (δ_C_ 172.7) confirming a C-1 CO_2_Me in aconitate A (**1**), (ii) the OMe (δ_H_ 3.81) and H_2_-2 (δ_H_ 3.89) to C-6 (δ_C_ 168.4) confirming a C-6 CO_2_Me in aconitate B (**2**), and (iii) the OMe (δ_H_ 3.77) and H-4 (δ_H_ 6.91) to C-5 (δ_C_ 167.5) confirming a C-5 CO_2_Me in aconitate C (**3**) (Figure 2).

HRESI(+)MS measurements suggested that **4** (C_8_H_10_O_6_, Δmmu +0.2) and **5** (C_8_H_10_O_6_, Δmmu +0.2) were isomeric dimethyl esters, and **6** (C_9_H_12_O_6_, Δmmu +0.1) was a trimethyl ester, of aconitic acid. Analysis of the NMR (methanol-*d*_4_) data for **4**–**6** (Figures S14–S19, Tables S3 and S4) confirmed these assignments, with *E* ∆^3,4^ configurations being inferred from diagnostic chemical shifts for H-4 and C-2 in aconitate D (**4**) (δ_H_ 6.91; δ_C_ 33.8), aconitate E (**5**) (δ_H_ 6.92; δ_C_ 33.9) and aconitate F (**6**) (δ_H_ 6.92; δ_C_ 33.8), and HMBC correlations permitting the assignment of the dimethyl ester regiochemistry across **4** and **5**. For example, correlations from an OMe (δ_H_ 3.67) and H_2_-2 (δ_H_ 3.91) to C-1 (δ_C_ 172.5), and from an OMe (δ_H_ 3.80) and H_2_-2 to C-6 (δ_C_ 168.2), confirmed the presence of C-1 CO_2_Me and C-6 CO_2_Me moieties in **4**, whereas correlations from an OMe (δ_H_ 3.67) and H_2_-2 (δ_H_ 3.91) to C-1 (δ_C_ 172.5), and from an OMe (δ_H_ 3.76) and H-4 (δ_H_ 6.92) to C-5 (δ_C_ 167.5), confirmed C-1 CO_2_Me and C-5 CO_2_Me moieties in **5** (Figure 2).

**Figure 3:**
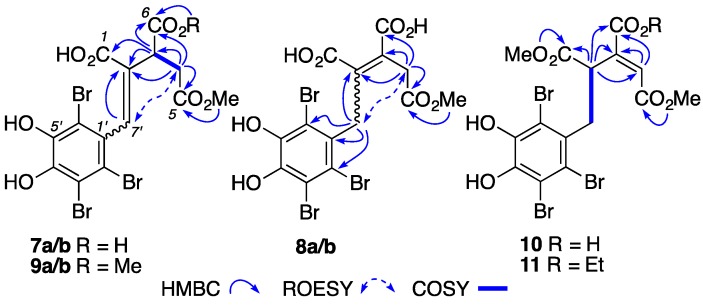
Diagnostic 2D NMR correlations for symphyocladins C/D (**7a/b**), H/I (**8a/b**), J/K (**9a/b**) and L–M (**10**–**11**) (see Tables and Supporting Information for NMR solvents).

HRESI(+)MS measurements confirmed that **7a/b** (C_14_H_11_Br_3_O_8_, Δmmu +0.5) and **8a/b** (C_14_H_11_Br_3_O_8_, ∆mmu +0.4) were isomeric, and suggested that **9a/b** (C_15_H_13_Br_3_O_8_, Δmmu +0.4) and **10** (C_15_H_13_Br_3_O_8_, Δmmu +0.5) were CH_2_ homologues, and **11** (C_17_H_17_Br_3_O_8_, Δmmu +0.6) was a CH_2_CH_2_ homologue of **7a/b** and **8a/b**. Analysis of the NMR (acetone-*d*_6_) data for **7a/b** (Figures S20 and S21, Table 1, Table 2 and Table S5) confirmed them as symphyocladins C/D, first reported in 2012 from *S. latiuscula* as an inseparable mixture of *Z/E* ∆^2,7′^ isomers [13]. Analysis of the NMR (methanol-*d*_4_) data for symphyocladins H/I (**8a/b**) (Figures S22–S27, Table 1, Table 2 and Table S6) revealed ∆^2,3^ and C-5 CO_2_Me moieties, as evidenced by diagnostic HMBC correlations (Figure 3). Significantly, these data also revealed an interconverting mixture of *E/Z* ∆^2,3^ isomers, in which the minor *Z* isomer, symphyocladin H (**8a**), as evidenced by a ROESY correlation between H_2_-4 and H_2_-7′ (Figure 3), was in equilibrium with the major *E* isomer, symphyocladin I (**8b**). Further analysis of this NMR data revealed chemical shift differences diagnostic for ∆^2,3^ geometric isomers; H_2_-4 (*E* δ_H_ 3.65, δ_C_ 35.0; *Z* δ_H_ 3.19, δ_C_ 29.5), C-1 (*E* δ_C_ 170.3; *Z* δ_C_ 166.5), C-3 (*E* δ_C_ 127.4; *Z* δ_C_ 137.8), and C-5 CO_2_Me (*E* δ_H_ 3.71; *Z* δ_H_ 3.51). Remarkably, the NMR (acetonitrile-*d*_3_) data for **8a/b** revealed a single *Z* isomer **8a**, as evidenced by simplified spectra, a ROESY correlation between H_2_-4 and H_2_-7′, and diagnostic chemical shifts (Figures S28 and S29, Table S7). We speculate that in aprotic solvents (i.e., acetonitrile-*d*_3_), hydrogen bonding between adjacent CO_2_H moieties exclusively favors the lower energy *Z* ∆^2,3^ isomer. By contrast, in protic solvents (i.e., methanol-*d*_4_), the disruption of this hydrogen bonding favor equilibration to an *E/Z* ∆^2,3^ mixture dominated by the less sterically constrained *E* isomer. This observation highlights the critical importance that NMR solvents can play in the analysis and structure elucidation of natural products.

Analysis of the NMR (DMSO-*d*_6_) data for symphyocladins J/K (**9a/b**) (Figures S30 and S31, Table 1, Table 2 and Table S8) identified an inseparable mixture of C-6 CO_2_Me homologues of **7a/b** and **8a/b**, as evidenced by spectroscopic comparisons and diagnostic HMBC correlations from the additional CO_2_Me resonances to C-6 (Figure 3). In this instance, as hydrogen bonding does not stabilize double bond isomers, the *Z/E* mixture prevails even in an aprotic solvent (i.e., DMSO-*d*_6_). Analysis of the NMR (acetone-*d*_6_) data for symphyocladin L (**10**) (Figures S32 and S33, Table 3, Table 5 and Table S9) revealed a ∆^3,4^ isomer and 1-CO_2_Me homologue of **7a/b** and **8a/b**, as evidenced by COSY correlations between H-2 and H_2_-7′, and diagnostic HMBC correlations positioning both C-1 CO_2_Me and C-5 CO_2_Me (Figure 3). The structure of **10** inclusive of an *E* ∆^3,4^ configuration and its racemic nature were confirmed by single crystal X-ray analysis with the compound crystallizing in a centrosymmetric space group (Figures S34 and S35). Analysis of the NMR (methanol-*d*_4_) data for symphyocladin M (**11**) (Figures S36 and S37, Table 3, Table 5 and Table S10) revealed it as a C-6 CO_2_Et homologue of **10**, as evidenced by spectroscopic comparisons and an HMBC correlation from the CO_2_Et moiety to C-6.

**Figure 4:**
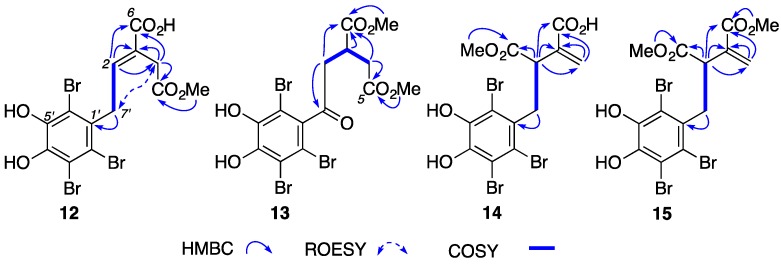
Diagnostic 2D NMR correlations for symphyocladins N–Q (**12**–**15**) (see Table 4 and Table 5 and Supporting Information for NMR solvents).

HRESI(+)MS measurements suggested that **12** (C_13_H_11_Br_3_O_6_, Δmmu +0.5) was a decarboxy analogue of **7a/b** and **8a/b**; **13** (C_14_H_13_Br_3_O_7_, Δmmu +0.5) was a dihydro oxidized methyl ester of **12**; **14** (C_13_H_11_Br_3_O_6_, Δmmu +0.5) was a decarboxymethyl analogue of **10**; and, **15** (C_14_H_13_Br_3_O_6_, Δmmu +0.5) was a CH_2_ homologue of **14**. Comparison of the NMR (methanol-*d*_4_) data for symphyocladin N (**12**) (Figures S38 and S39, Table 4, Table 5 and Table S11) with that for **8a/b** revealed the key difference as replacement of the C-1 CO_2_H moiety in **8a/b** with an H-2 olefinic methine (δ_H_ 6.79) coupled to H_2_-7′ (δ_H_ 4.05). The presence of a C-5 CO_2_Me moiety in **12** was evident from an HMBC correlation from the OMe (δ_H_ 3.66) to C-5 (δ_C_ 171.3), while an *E* Δ^2,3^ configuration was confirmed by a ROESY correlation between H_2_-4 (δ_H_ 3.60) and H_2_-7′ (Figure 4). Analysis of the NMR (methanol-*d*_4_) data for symphyocladin O (**13**) (Figures S40 and S41, Table 4, Table 5, and Table S12) revealed it to be a saturated oxidized analogue of **12**, as evidenced by COSY correlations between a diastereotopic H_2_-2 (δ_H_ 3.35/3.27), through H-3 (δ_H_ 3.34) to a diastereotopic H_2_-4 (δ_H_ 2.86/2.73). Likewise, replacement of the C-7′ sp^3^ methylene in **12** (δ_C_ 39.0) with a carbonyl resonance in 13 (δ_C_ 202.2) was evidence of a 7-oxo moiety. Diagnostic HMBC correlations also established the presence of incorporated C-5 CO_2_Me (δ_H_ 3.68) and C-6 CO_2_Me (δ_H_ 3.71) moieties (see Figure 3).

Comparison of the NMR (methanol-*d*_4_) data for symphyocladin P (**14**) (Figures S42 and S43, Table 4, Table 5 and Table S13) with that for **10** revealed the key difference as replacement of the C-5 CO_2_Me moiety in **10** with a diastereotopic H_2_-4 olefinic methylene (δ_H_ 6.22/5.44). Comparison of the NMR (acetone-*d*_6_) data for symphyocladin Q (**15**) (Figures S44 and S45, Table 4, Table 5 and Table S14) with that for **14** revealed the key difference as an additional resonance, attributed to a C-6 CO_2_Me moiety (δ_H_ 3.71). Structure assignments for **14** and **15** were further supported by diagnostic 2D NMR correlations (Figure 4).

**Figure 5:**
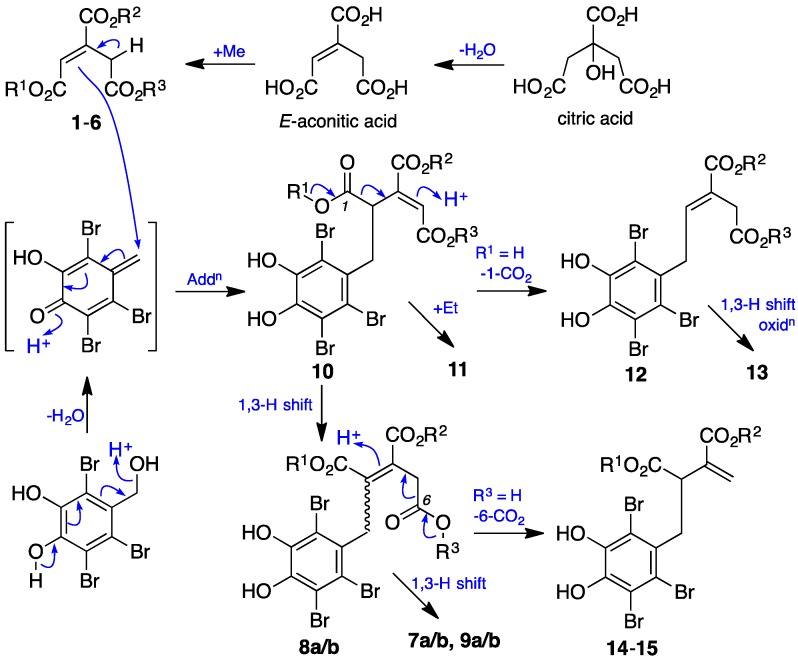
A plausible biosynthetic relationship linking **1**–**15.**

Structural similarities across **1**–**15** suggest a highly conserved biosynthesis. Building on this observation, we propose a biosynthetic relationship (Figure 5), in which the aconitates A–F (**1**–**6**) are viewed as mono, di, and tri methyl esters of the precursor *E*-aconitic acid, itself a dehydration product of citric acid. Likewise, metabolites **7**–**15** can be viewed as adducts between aconitates and an intermediate quinone methide that is generated from 2,3,6-tribromo-4,5-dihydroxybenzyl alcohol, further elaborated by a combination of 1,3-hydride shifts, decarboxylations and oxidations. Although **7a/b**, **9a/b**, **10**–**11,** and **13** incorporate a single chiral sp^3^ center, as they do not exhibit measurable optical rotations they are presumed to be racemic, as confirmed for **10** by X-ray crystallography. The absence of double bond migrations (i.e., racemization) during isolation and handling suggests that this racemic character is a function of achiral adduct addition. The proposed biosynthetic relationship informs a possible biomimetic synthesis of **7**–**15**, although, in our hands, synthetic 2,3,6-tribromo-4,5-dihydroxybenzyl alcohol proved stable to both acid and base conditions indicative of a requirement to activate the benzyl alcohol moiety to effect dehydration and the formation of a quinone methide.

## 3. Materials and Methods

**General Experimental Procedures.** Specific optical rotations ([α]_D_) were measured on a polarimeter in a 100 × 2 mm cell at 22 °C. NMR spectra were obtained on a Bruker Avance DRX600 or DRX500 spectrometers, in the solvents indicated and referenced to residual ^1^H and ^13^C signals in deuterated solvents. Electrospray ionization mass spectra (ESIMS) were acquired using an Agilent 1100 Series separations module equipped with an Agilent 1100 Series LC/MSD mass detector in both positive and negative ion modes. High-resolution ESIMS measurements were obtained on a Bruker micrOTOF mass spectrometer by direct infusion in MeCN at 3 mL/min using sodium formate clusters as an internal calibrant. HPLC was performed using an Agilent 1100 Series separations module equipped with Agilent 1100 Series diode array and/or multiple wavelength detectors and Agilent 1100 Series fraction collector, controlled using ChemStation Rev.B02.01 and Purify version A.1.2 software.

**Algal material.** *Symphyocladia latiuscula* was collected on the coast of Qingdao, Shandong Province, China, in May 2004. The specimen identification was verified by Dr. Kui-Shuang Shao (Institute of Oceanology, Chinese Academy of Sciences, Qingdao 266071, China). A voucher specimen (No. 2004X16) was deposited at the Herbarium of the Institute of Oceanology, Chinese Academy of Sciences, Qingdao 266071, China.

**Extraction and isolation.** The air-dried red alga *Symphyocladia latiuscula* (4.3 kg) was extracted with 95% EtOH at room temperature (3 × 72 h). After the solvent was removed under reduced pressure at <40 °C, a dark residue (610 g) was obtained. The residue was partitioned between EtOAc and H_2_O, and the EtOAc-soluble partition (320 g) was chromatographed over silica gel, eluting with a gradient of 0–100% Me_2_CO/petroleum ether to yield 85 fractions (F1–F85) (see Supporting Information Scheme S1 for the fractionation scheme). Fraction F54 was further fractionated over Sephadex LH-20 using CHCl_3_–MeOH (2:1) to afford 21 fractions.

The sixth fraction from Sephadex LH-20 chromatography was fractionated on an ODS column, eluted by a stepwise gradient (0–100% MeOH/H_2_O) to afford 11 fractions. The third fraction was subjected to HPLC separation (Zorbax Eclipse XDB-C_8_, 5 μm, 250 × 9.4 mm column, 3.0 mL/min, gradient elution from 10 to 80% MeCN/H_2_O over 15 min, with isocratic 0.01% TFA modifier) to yield **1**–**6**; the sixth fraction was subjected to HPLC separation (Zorbax SB-C_18_, 5 μm, 250 × 9.4 mm column, 3.0 mL/min, gradient elution from 30 to 75% MeCN/H_2_O over 14 min, with isocratic 0.01% TFA modifier) to yield **8a/b** and **9a/b**; and the seventh fraction was subjected to HPLC separation (Zorbax Eclipse XDB-C_8_, 5 μm, 250 × 9.4 mm column, 3.0 mL/min, gradient elution from 40 to 55% MeCN/H_2_O over 20 min, with isocratic 0.01% TFA modifier) to yield **11** and **13**.

The seventh fraction from Sephadex LH-20 chromatography was subjected to HPLC fractionation (Zorbax SB-C_18_, 5 um, 250 × 9.4 mm column, 3.0 mL/min, gradient elution from 30 to 80% MeCN/H_2_O over 14 min, with isocratic 0.01% TFA modifier) to yield **10**.

The eighth fraction from Sephadex LH-20 chromatography was fractionated on an ODS column, eluted with a stepwise gradient (0–100% MeOH/H_2_O) to afford 11 fractions; the sixth fraction was subjected to HPLC fractionation (Zorbax SB-C_18_, 5 δm, 250 × 9.4 mm column, 3.0 mL/min, gradient elution from 35 to 50% MeCN/H_2_O over 15 min, with isocratic 0.01% TFA modifier) to yield **14**; the seventh fraction was subjected to HPLC fractionation (Zorbax SB-C_18_, 5 μm, 250 × 9.4 mm column, 3.0 mL/min, gradient elution from 50 to 60% MeCN/H_2_O over 12 min, with isocratic 0.01% TFA modifier) to yield **12** and **15**.

The ninth fraction from Sephadex LH-20 chromatography was fractionated on an ODS column, eluted with a stepwise gradient (0–100% MeOH/H_2_O) to afford 11 fractions; the third fraction was subjected to HPLC fractionation (Zorbax SB-C_18_, 5 μm, 250 × 9.4 mm column, 3.0 mL/min, gradient elution from 30 to 80% MeCN/H_2_O over 20 min, with isocratic 0.01% TFA modifier) to yield **7a/b**.

*Aconitate A* (**1**): White solid; NMR (600 MHz, methanol-*d*_4_) see Table S2; HRESIMS *m/z* 189.0396 [M + H]^+^ (calcd. for C_7_H_8_O_6_ 189.0394).

*Aconitate B* (**2**): White solid; NMR (600 MHz, methanol-*d*_4_) see Table S2; HRESIMS *m/z* 189.0395 [M + H]^+^ (calcd. for C_7_H_8_O_6_ 189.0394).

*Aconitate C* (**3**): White solid; NMR (600 MHz, methanol-*d*_4_) see Table S3; HRESIMS *m/z* 189.0395 [M + H]^+^ (calcd. for C_7_H_8_O_6_ 189.0394).

*Aconitate D* (**4**): White solid; NMR (600 MHz, methanol-*d*_4_) see Table S3; HRESIMS *m/z* 203.0552 [M + H]^+^ (calcd. for C_8_H_11_O_6_, 203.0550)

*Aconitate E* (**5**): White solid; NMR (600 MHz, methanol-*d*_4_) see Table S4; HRESIMS *m/z* 203.0551 [M + H]^+^ (calcd. for C_8_H_11_O_6_, 203.0550).

*Aconitate F* (**6**): White solid; NMR (600 MHz, methanol-*d*_4_) see Table S4; HRESIMS *m/z* 217.0708 [M + H]^+^ (calcd. for C_9_H_13_O_6_, 217.0707).

*Symphyocladins C/D* (**7a/b**): Light brown solid; NMR (600 MHz, acetone-*d*_6_) see Table 1, Table 2 and Table S5; HRESIMS *m/z* 544.8082 [M + H]^+^ (calcd. for C_14_H_12_Br_3_O_8_, 544.8077).

*Symphyocladins H/I* (**8a/b**): Light brown solid; NMR (600 MHz, methanol-*d*_4_, acetonitrile-*d*_3_) see Table 1, Table 2, Tables S6 and S7; HRESIMS *m/z* 544.8081 [M + H]^+^ (calcd. for C_14_H_12_Br_3_O_8_, 544.8077).

*Symphyocladins J/K* (**9a/b**): Light brown solid; NMR (600 MHz, DMSO-*d*_6_) see Table 1, Table 2 and Table S8; HRESIMS *m/z* 558.8237 [M + H]^+^ (calcd. for C_15_H_14_Br_3_O_8_, 558.8233).

*Symphyocladin L* (**10**): Light brown solid; NMR (600 MHz, acetone-*d*_6_) see Table 3, Table 5 and Table S9; HRESIMS *m/z* 558.8238 [M + H]^+^ (calcd. for C_15_H_14_Br_3_O_8_, 558.8233).

*Symphyocladin M* (**11**): Light brown solid; NMR (600 MHz, methanol-*d*_4_) see Table 3, Table 5 and Table S10; HRESIMS *m/z* 586.8552 [M + H]^+^ (calcd. for C_17_H_17_Br_3_O_8_, 586.8546).

*Symphyocladin N* (**12**): Light brown solid; NMR (600 MHz, acetone-*d*_6_) see Table 4, Table 5 and Table S11; HRESIMS *m/z* 500.8184 [M + H]^+^ (calcd. for C_13_H_12_Br_3_O_6_, 500.8179).

*Symphyocladin O* (**13**): Light brown solid; NMR (600 MHz, methanol-*d*_4_) see Table 4, Table 5 and Table S12; HRESIMS *m/z* 530.8289 [M + H]^+^ (calcd. for C_14_H_14_Br_3_O_7_, 530.8284).

*Symphyocladin P* (**14**): Light brown solid; NMR (600 MHz, methanol-*d*_4_) see Table 4, Table 5 and Table S13; HRESIMS *m/z* 500.8184 [M + H]^+^ (calcd. for C_13_H_12_Br_3_O_6_, 500.8179).

*Symphyocladin Q* (**15**): Light brown solid; NMR (600 MHz, acetone-*d*_6_) see Table 3, Table 4 and Table S14; HRESIMS *m/z* 514.8340 [M + H]^+^ (calcd. for C_14_H_14_Br_3_O_6_, 514.8335).

**X-ray crystallography.** X-ray crystallographic data were collected on an Oxford Diffraction Gemini CCD diffractometer with Mo-Kα radiation (0.71073 Å) operating within the range 2 < 2θ < 50°. The sample was cooled to 190 K with an Oxford Cryosystems Desktop Cooler. Data reduction and empirical absorption corrections were performed using CrysAlisPro (Rigaku Oxford Diffraction, Yarnton, Oxfordshire, UK). The structure was solved by Direct Methods and refined with SHELX [21] and all of the calculations and refinements were carried out by WinGX package [22]. All non-H atoms were refined aniostropically. The thermal ellipsoid plot was produced with ORTEP [23] and the unit cell diagram was drawn with PLATON [24]. Crystallographic data including structure factors in CIF format have been deposited with the Cambridge Crystallographic Data Centre (CCDC 1569026). 

## Figures and Tables

**Table 1:** ^1^H NMR Data for Compounds **7a/b**–**9a/b** (600 MHz).

Position	7a ^a^	7b ^a^	8a ^b^	8b ^b^	9a ^c^	9b ^c^
3	3.74, m	3.74, m			3.50, m	3.50, m
4a	3.17, m	3.17, m	3.65, s	3.19, s	2.98, dd ^e^	2.976, dd ^e^
4b	2.490, dd ^d^	2.489, dd ^d^			2.41, dd ^f^	2.40, dd ^f^
5-OCH3	3.55, s	3.55, s	3.71, s	3.51, s	3.52, s	3.52, s
6-OCH3					3.541, s	3.535, s
7′	7.544, s	7.538, s	4.22, s	4.34, s	7.391, s	7.388, s

^a^ acetone-*d*_6_, ^b^ methanol-*d*_4_, ^c^ DMSO-*d*_6_, ^d^ *J* = 16.8, 7.8 Hz, ^e^ *J* = 16.8, 10.8 Hz, ^f^ *J* = 16.8, 3.0 Hz.

**Table 2:** ^13^C NMR Data for Compounds **7a/b**–**9a/b** (150 MHz).

Position	7a ^a^	7b ^a^	8a ^b^	8b ^b^	9a ^c^	9b ^c^
1	167.18, C	167.18, C	170.3, C	166.5, C	166.8, C	166.8, C
2	135.46, C	135.34, C	145.0, C	144.4, C	133.5, C	133.5, C
3	41.39, CH	41.37, CH	127.4, C	137.8, C	40.2, CH	40.2, CH
4	35.40, CH_2_	35.35, CH_2_	35.0, CH_2_	29.5, CH_2_	33.9, CH_2_	33.9, CH_2_
5	172.80, C	172.76, C	168.7, C	166.8, C	171.5, C	171.5, C
6	172.54, C	172.49, C	172.4, C	169.0, C	171.5, C	171.5, C
5-OCH_3_	51.87, CH_3_	51.83, CH_3_	52.8, CH_3_	53.1, CH_3_	52.0, CH_3_	52.0, CH_3_
6-OCH_3_					51.6, CH_3_	51.6, CH_3_
1′	129.79, C	129.76, C	128.8, C	127.9, C	128.07, C	128.07, C
2′	115.38, C	115.23, C	118.9, C	118.4, C	113.9, C	113.9, C
3′	113.98, C	113.63, C	114.8, C	114.2, C	113.7, C	113.6, C
4′	144.17, C	144.10, C	146.2, C	145.5, C	143.9, C	143.8, C
5′	145.36, C	145.32, C	145.6, C	144.8, C	145.2, C	145.0, C
6′	110.71, C	110.67, C	114.7, C	114.3, C	110.7, C	110.6, C
7′	141.52, C	141.48, C	41.1, CH	35.9, CH	140.7, C	140.7, C

^a^ acetone-*d*_6_, ^b^ methanol-*d*_4_, ^c^ DMSO-*d*_6_.

**Table 3:** ^1^H NMR Data for Compounds **10**–**11** (600 MHz).

Position	10 ^a^ δ_H_, m (J in Hz)	11 ^b^ δ_H_, m (J in Hz)
2	4.98, dd (11.4, 3.0)	4.98, dd (11.4, 3.0)
4	6.76, s	6.73, s
1-OCH_3_	3.66, s	3.70, s
5-OCH_3_	3.44, s	3.45, s
6-OCH_2_CH_3_		4.26, br q (7.2)
6-OCH_2_CH_3_		1.31, t (7.2)
7′a	3.87, dd (14.4, 3.0)	3.81, dd (14.4, 3.0)
7′b	3.61, dd (14.4, 11.4)	3.56, dd (14.4, 11.4)

^a^ acetone-*d*_6_, ^b^ methanol-*d*_4_.

**Table 4:** ^1^H NMR Data for Compounds **12**–**15** (600 MHz).

Position	12 ^a^ δ_H_, m (*J* in Hz)	13 ^b^ δ_H_, m (*J* in Hz)	14 ^b^ δ_H_, m (*J* in Hz)	15 ^a^ δ_H_, m (*J* in Hz)
2a	6.79, br t (6.6)	3.35, m	3.75, dd (9.0, 6.0^)^	3.79, dd (9.6, 5.4)
2b		3.27, dd (20.4, 7.8)		
3		3.34, m		
4a	3.60, br s	2.86, dd (17.4, 7.2)	6.22. d (1.2)	6.17, d (1.2)
4b		2.73, dd (17.4, 6.0)	5.44, br s	5.52, s
1-OCH_3_			3.64, s	3.62, s
5-OCH_3_	3.66, s	3.68, s		
6-OCH_3_		3.71, s		3.71, s
7′a	4.05, s		3.65, dd (13.8, 6.0)	3.69, dd (14.4, 5.4)
7′b			3.54, dd (13.8, 9.0)	3.56, dd (14.4, 9.6)

^a^ acetone-*d*_6_, ^b^ methanol-*d*_4_.

**Table 5:** ^13^C NMR Data for Compounds **10**–**15** (150 MHz).

Position	10 ^a^ δ_C_, Type	11 ^b^ δ_C_, Type	12 ^a^ δ_C_, Type	13 ^b^ δ_C_, Type	14 ^b^ δ_C_, Type	15 ^a^ δ_C_, Type
1	171.9, C	173.5, C			174.5, C	172.4, C
2	43.0, C	43.7, C	141.3, C	44.9, CH_2_	48.8, CH	48.2, CH
3	142.4, C	142.4, C	128.1, C	37.4, CH	139.3, C	138.3, C
4	130.5, CH	131.3, CH	33.2, CH_2_	35.6, CH_2_	129.1, CH_2_	128.7, CH_2_
5	165.8, C	166.7, C	171.3, C	173.9, C		
6	167.0, C	167.0, C	167.9, C	175.5, C	169.2, C	166.6, C
1-OCH_3_	52.3, CH_3_	52.9, CH_3_			52.8, CH_3_	52.3, CH_3_
5-OCH_3_	52.1, CH_3_	52.5, CH_3_	52.0, CH_3_	52.5, CH_3_		
6-OCH_3_				52.8, CH_3_		52.3, CH_3_
6-OCH_2_CH_3_		63.2, C				
6-OCH_2_CH_3_		14.5, CH_3_				
1′	130.7, C	130.6, C	131.0, C	136.1, C	131.4, C	131.3, C
2′	118.5, C	118.8, C	117.3, C	114.5, C	118.3, C	118.0, C
3′	113.7, C	114.4, C	114.0, C	110.5, C	114.5, C	113.9, C
4′	144.1, C	145.1, C	144.4, C	147.3, C	145.0, C	144.0, C
5′	143.8, C	144.8, C	144.3, C	145.3, C	144.8, C	143.9, C
6′	114.3, C	114.8, C	113.0, C	106.3, C	114.3, C	113.8, C
7′	39.0, CH_2_	39.4, CH_2_	39.0, CH_2_	202.2, C	39.4, CH_2_	39.1, CH_2_

^a^ acetone-*d*_6_, ^b^ methanol-*d*_4_.

## Supplementary Materials

The following are available online at www.mdpi.com/1660-3397/15/12/374/s1, Isolation Scheme as well as Tabulated 1D and 2D NMR data and spectra for 1–15, X-ray of compound **10**.
