# Supplementary material for: Chemical Diversity from a Chinese Marine Red Alga, Symphyocladia latiuscula
Source: Mar Drugs. 2017 Dec 1;15(12):374. doi: 10.3390/md15120374 (PMC5742834; doi:10.3390/md15120374)
Supplement: Supplementary file 1 [file marinedrugs-15-00374-s001.pdf]

SUPPORTING INFORMATION

Chemical diversity from a Chinese marine red alga, *Symphyocladia latiuscula*

Xiuli Xu[1], Haijin Yang[1,2], Zeinab G. Khalil[3], Liyuan Yin[1], Xue Xiao[3], Pratik Neupane[3], Paul Bernhardt[4], Angela A. Salim[3], Fuhang Song[1*] and Robert J. Capon[3*]

[1]School of Ocean Sciences, China University of Geosciences, Beijing 100083, P. R. China

[2]CAS Key Laboratory of Pathogenic Microbiology and Immunology, Institute of Microbiology, Chinese Academy of Sciences, Beijing, 100101, P. R. China

[3]Institute for Molecular Bioscience, The University of Queensland, Brisbane, QLD 4072, Australia

[4]School of Chemistry and Molecular biosciences, The University of Queensland, Brisbane, QLD, 4072, Australia

Table of Contents

Scheme S1. Isolation scheme of **1 - 15**. 4

List of Tables

Table S1. 1D and 2D NMR data (500 MHz, methanol-d_4) of *Z*- and *E*-aconitic acids 8
Table S2. 1D and 2D NMR data (600 MHz, methanol-d_4) of aconitates A-B (**1**-**2**) 11
Table S3. 1D and 2D NMR data (600 MHz, methanol-d_4) of aconitates C-D (**3-4**) 14
Table S4. 1D and 2D NMR data (600 MHz, methanol-d_4) of aconitates E-F (**5-6**) 17
Table S5. 1D and 2D NMR data (600 MHz, acetone-d_6) of symphyocladins C/D (**7a/b**) 19
Table S6. 1D and 2D NMR data (600 MHz, methanol-d_4) of symphyocladins H/I (**8a/b**) 24
Table S7. 1D and 2D NMR data (600 MHz, acetonitrile-d_3) of symphyocladin H (**8a**) 26
Table S8. 1D and 2D NMR data (600 MHz, DMSO-d_6) of symphyocladins J/K (**9a/b**) 28
Table S9. 1D and 2D NMR data (600 MHz, acetone-d_6) of symphyocladin L (**10**) 30
Table S10. 1D and 2D NMR data (600 MHz, methanol-d_4) of symphyocladin M (**11**) 33
Table S11. 1D and 2D NMR data (600 MHz, acetone-d_6) of symphyocladin N (**12**) 35
Table S12. 1D and 2D NMR data (600 MHz, methanol-d_4) of symphyocladin O (**13**) 37
Table S13. 1D and 2D NMR (600 MHz, methanol-d_4) of symphyocladin P (**14**) 39
Table S14. 1D and 2D NMR data (600 MHz, acetone-d_6) of symphyocladin Q (**15**) 41

List of Figures

Figure S1. ^{1}H NMR (methanol-d_4) spectrum of *Z*-aconitic acid 5
Figure S2. ^{13}C NMR (methanol-d_4) spectrum of *Z*-aconitic acid 5
Figure S3. HMBC spectrum (methanol -d_4) of *Z*-aconitic acid 6
Figure S4. ROESY (methanol-d_4) spectrum of *Z*-aconitic acid 6
Figure S5. ^{1}H NMR (methanol-d_4) spectrum of *E*-aconitic acid 7
Figure S6. ^{13}C NMR (methanol-d_4) spectrum of *E*-aconitic acid 7
Figure S7. HMBC spectrum (methanol-d_4) for *E*-aconitic acid 8
Figure S8. ^{1}H NMR (methanol-d_4) spectrum of aconitate A (**1**) 9
Figure S9. ^{13}C NMR (methanol-d_4) spectrum of aconitate A (**1**) 9
Figure S10. ^{1}H NMR (methanol-d_4) spectrum of aconitate B (**2**) 10
Figure S11. ^{13}C NMR (methanol-d_4) spectrum of aconitate B (**2**) 10
Figure S12. ^{1}H NMR (methanol-d_4) spectrum of aconitate C (**3**) 12
Figure S13. ^{13}C NMR (methanol-d_4) spectrum of aconitate C (**3**) 12
Figure S14. ^{1}H NMR (methanol-d_4) spectrum of aconitate D (**4**) 13
Figure S15. ^{13}C NMR (methanol-d_4) spectrum of aconitate D (**4**) 13
Figure S16. ^{1}H NMR (methanol-d_4) spectrum of aconitate E (**5**) 15
Figure S17. ^{13}C NMR (methanol-d_4) spectrum of aconitate E (**5**) 15
Figure S18. ^{1}H NMR (methanol-d_4) spectrum of aconitate F (**6**) 16
Figure S19. ^{13}C NMR (methanol-d_4) spectrum of aconitate F (**6**) 16
Figure S20. ^{1}H NMR (acetone-d_6) spectrum of symphyocladins C/D (**7a/b**) 18
Figure S21. ^{13}C NMR (acetone-d_6) spectrum of symphyocladins C/D (**7a/b**) 18
Figure S22. ^{1}H NMR (methanol-d_4) spectrum of symphyocladins H/I (**8a/b**) (prior to equil) 20
Figure S23. ^{1}H NMR (methanol-d_4) spectrum of symphyocladins H/I (**8a/b**) (after overnight) 21
Figure S24. ^{13}C NMR (methanol-d_4) spectrum of symphyocladins H/I (**8a/b**) 22
Figure S25. HSQC (methanol-d_4) spectrum of symphyocladins H/I (**8a/b**) 22
Figure S26. HMBC (methanol-d_4) spectrum of symphyocladins H/I (**8a/b**) 23
Figure S27. ROESY (methanol-d_4) spectrum of symphyocladins H/I (**8a/b**) 23
Figure S28. ^{1}H NMR (acetonitrile-d_3) spectrum of symphyocladin H (**8a**) 25
Figure S29. ^{13}C NMR (acetonitrile-d_3) spectrum of symphyocladin H (**8a**) 25
Figure S30. ^{1}H NMR (DMSO-d_6) spectrum of symphyocladins J/K (**9a/b**) 27
Figure S31. ^{13}C NMR (DMSO-d_6) spectrum of symphyocladins J/K (**9a/b**) 27

Figure S32. ^{1}H NMR (acetone-d_6) spectrum of symphyocladin L (**10**) 29
Figure S33. ^{13}C NMR (acetone-d_6) spectrum of symphyocladin L (**10**) 29
Figure S34. X-ray of symphyocladin L (**10**) 31
Figure S35. X-ray of symphyocladin L (**10**) (unit cell).. 31
Figure S36. ^{1}H NMR (methanol-d_4) spectrum of symphyocladin M (**11**) 32
Figure S37. ^{13}C NMR (methanol-d_4) spectrum of symphyocladin M (**11**) 32
Figure S38. ^{1}H NMR (acetone-d_6) spectrum of symphyocladin N (**12**)... 34
Figure S39. ^{13}C NMR (acetone-d_6) spectrum of symphyocladin N (**12**).. 34
Figure S40. ^{1}H NMR (methanol-d_4) spectrum of symphyocladin O (**13**) 36
Figure S41. ^{13}C NMR (methanol-d_4) spectrum of symphyocladin O (**13**) 36
Figure S42. ^{1}H NMR (methanol-d_4) spectrum of symphyocladin P (**14**).. 38
Figure S43. ^{13}C NMR (methanol-d_4) spectrum of symphyocladin P (**14**)....................................... 38
Figure S44. ^{1}H NMR (acetone-d_6) spectrum of symphyocladin Q (**15**)... 40
Figure S45. ^{13}C NMR (acetone-d_6) spectrum of symphyocladin Q (**15**).. 40

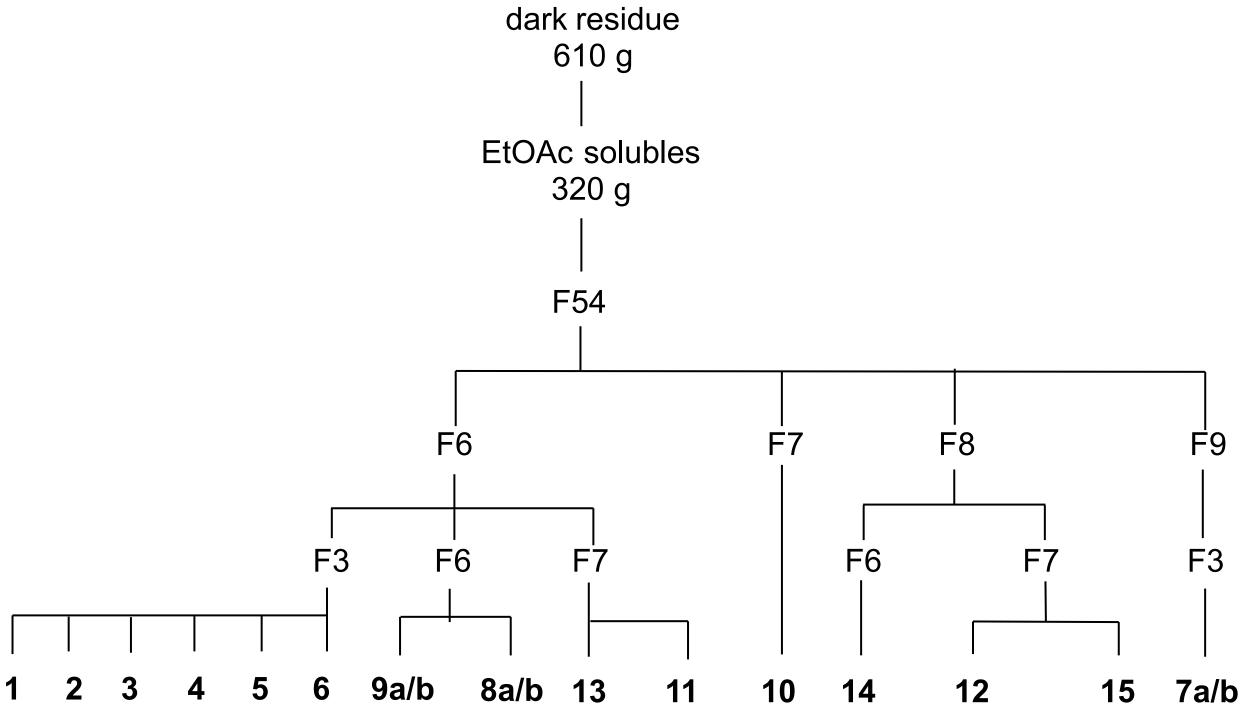

Scheme S1. Isolation scheme of **1** - **15**

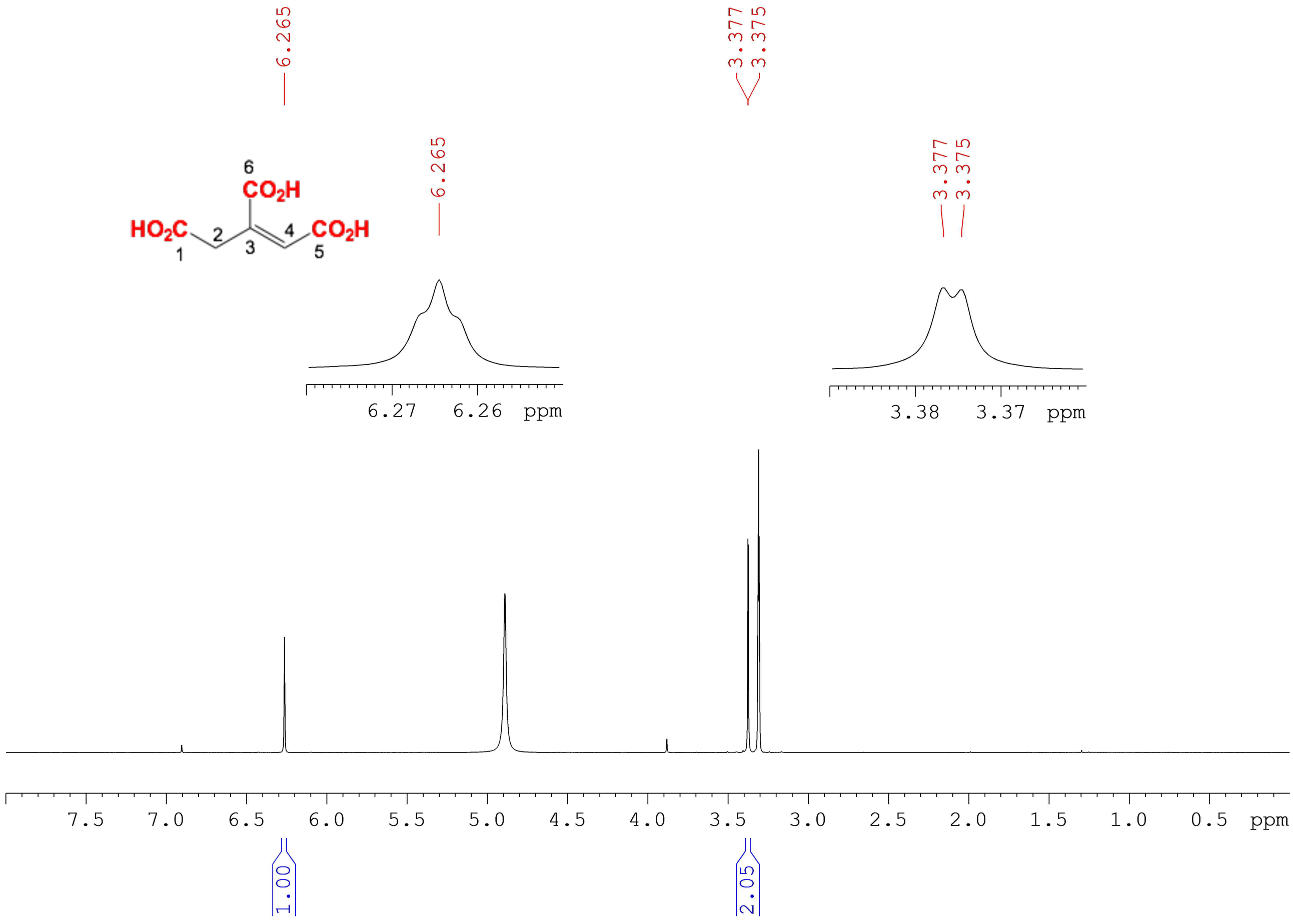

Figure S1. ^{1}H NMR (methanol-d_4) spectrum of *Z*-aconitic acid

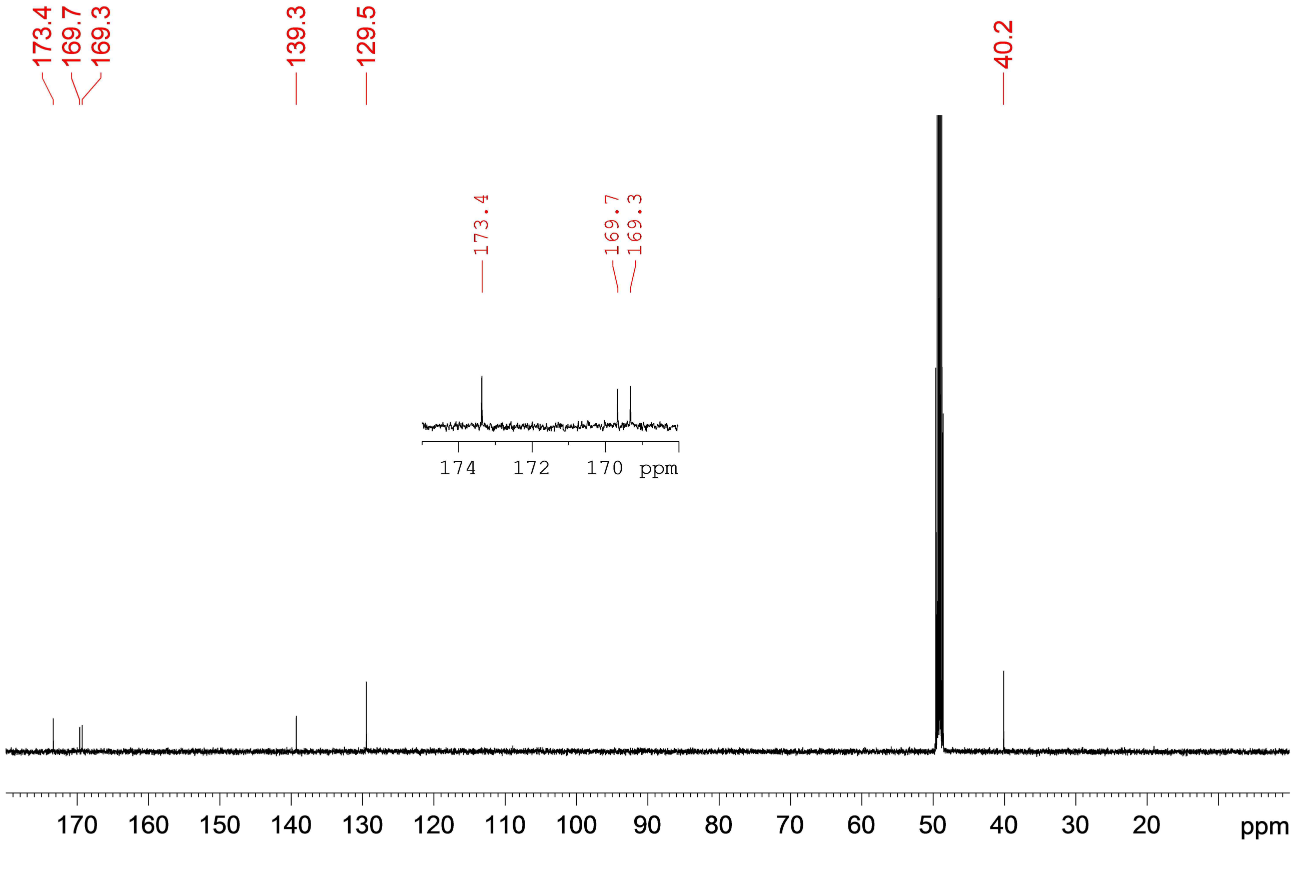

Figure S2. ^{13}C NMR (methanol-d_4) spectrum of *Z*-aconitic acid

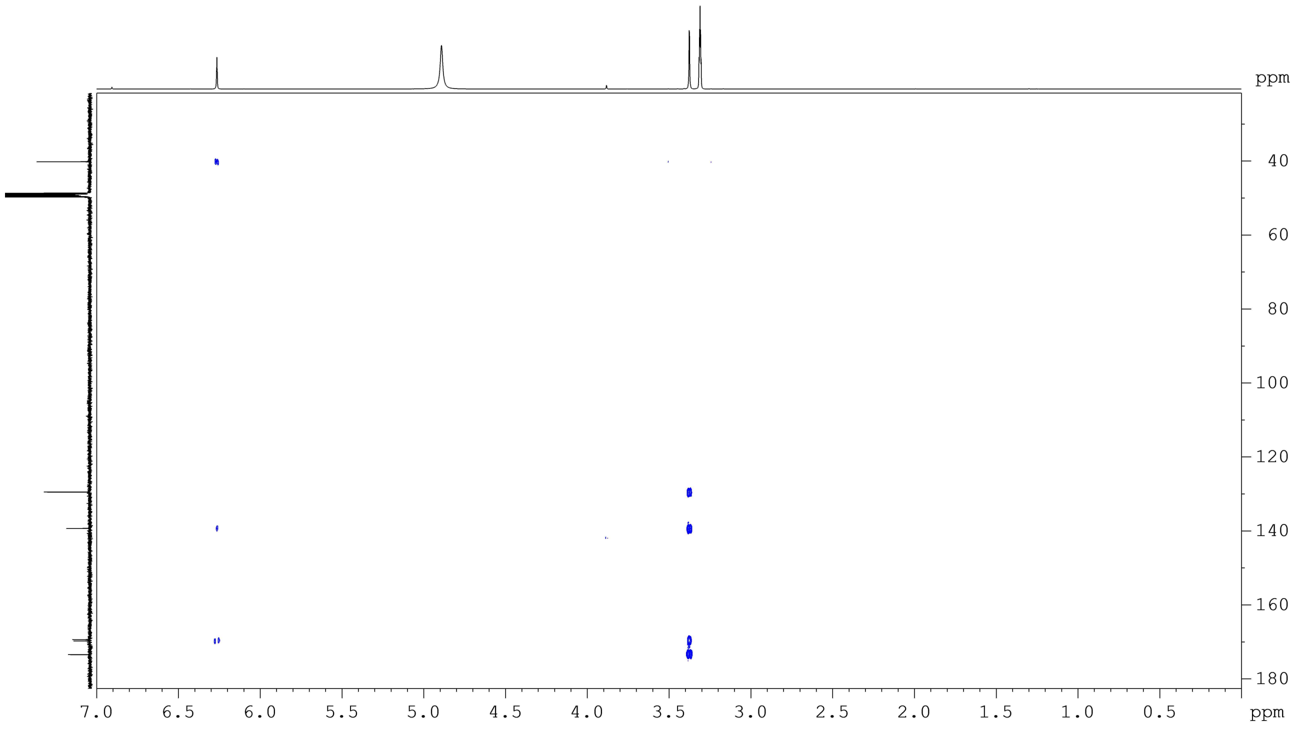

Figure S3. HMBC spectrum (methanol -d_4) of *Z*-aconitic acid

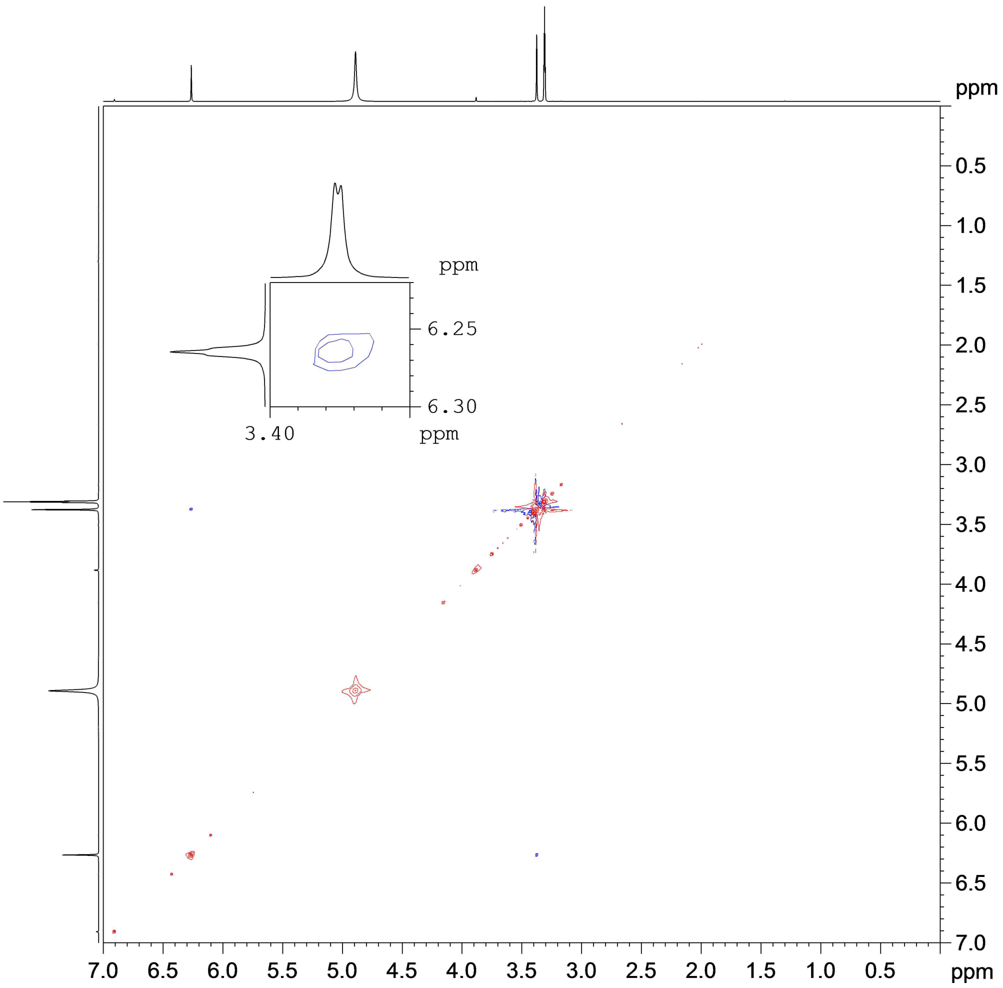

Figure S4. ROESY (methanol-d_4) spectrum of *Z*-aconitic acid

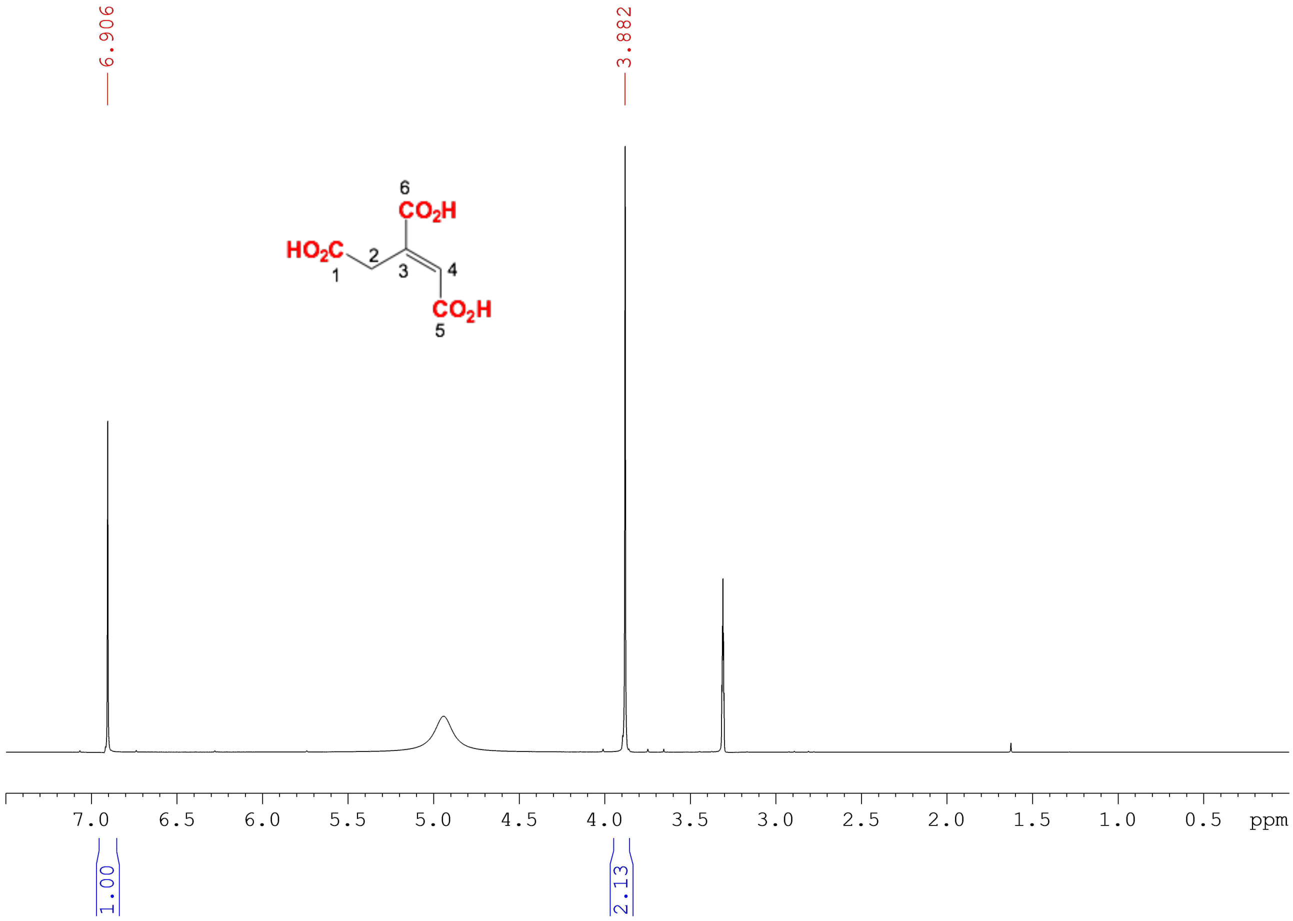

Figure S5. ^{1}H NMR (methanol-d_4) spectrum of *E*-aconitic acid

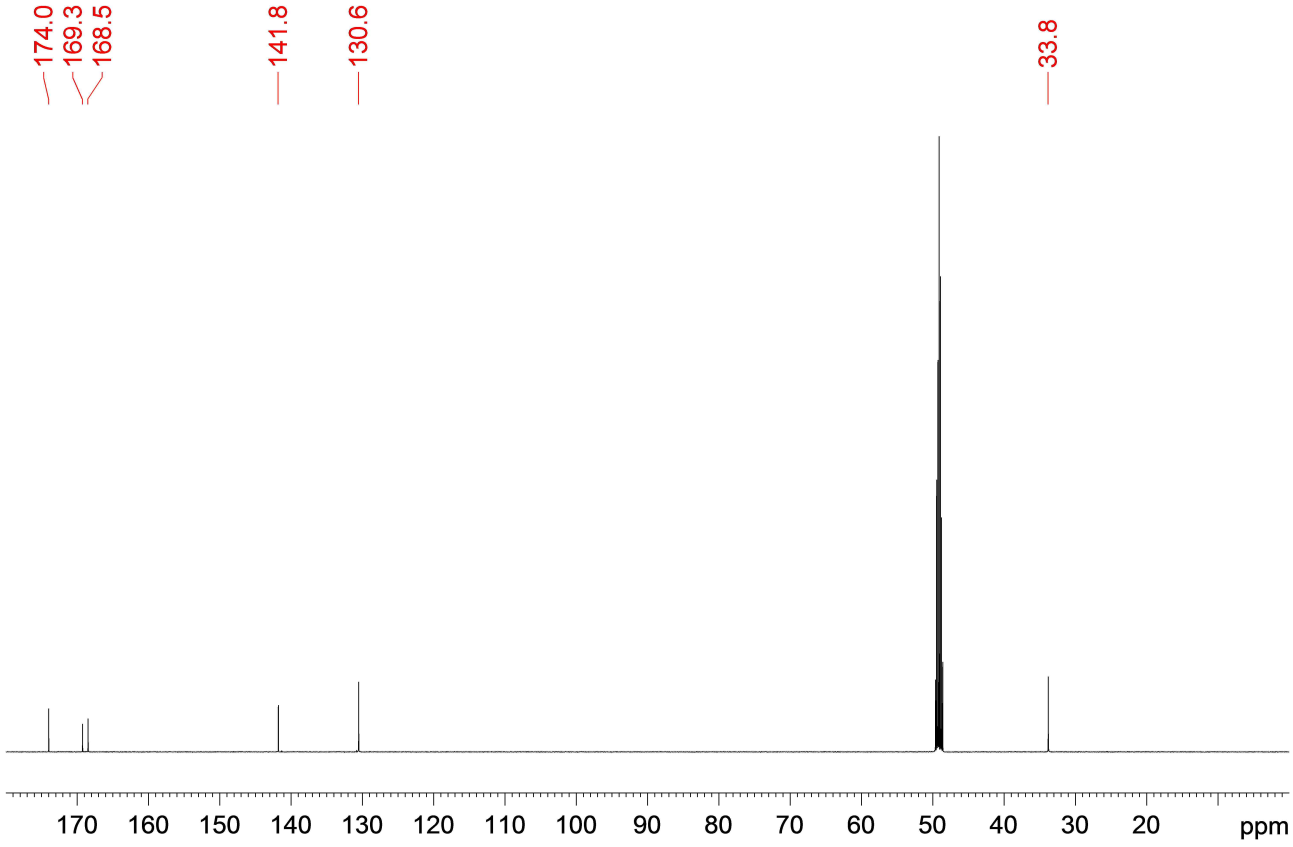

Figure S6. ^{13}C NMR (methanol-d_4) spectrum of *E*-aconitic acid

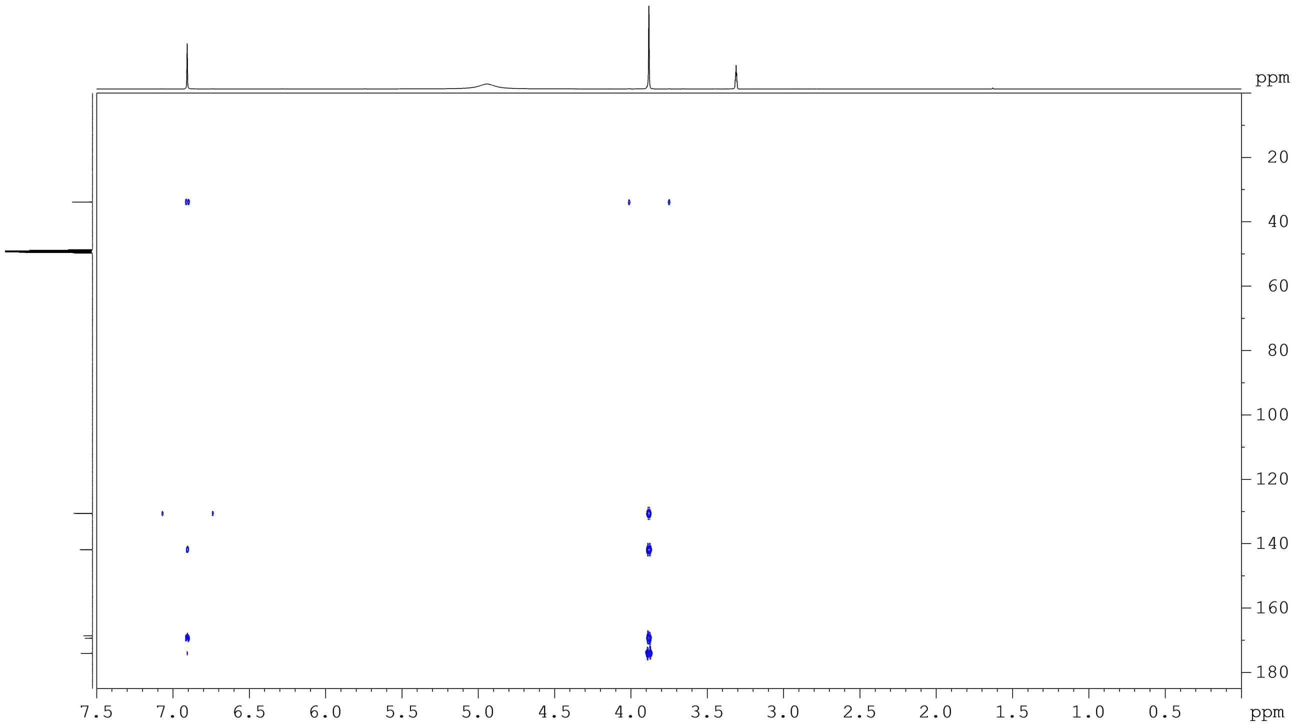

Figure S7. HMBC spectrum (methanol-*d*$_4$) for *E*-aconitic acid

Table S1. 1D and 2D NMR (methanol-*d*$_4$) data for *Z*- and *E*-aconitic acids

pos	*Z*-aconitic acids			*E*-aconitic acids		
	$\delta_{H,}$ mult (*J* in Hz)	δ_C	ROESY	$\delta_{H,}$ mult (*J* in Hz)	δ_C	ROESY
1		173.4			174.0	
2	3.38, d (1.0)	40.2	4	3.88, s	33.8	
3		139.3			141.8	
4	6.26, br t (1.0)	129.5	2	6.90, s	130.6	
5		169.3			168.5	
6		169.7			169.3	

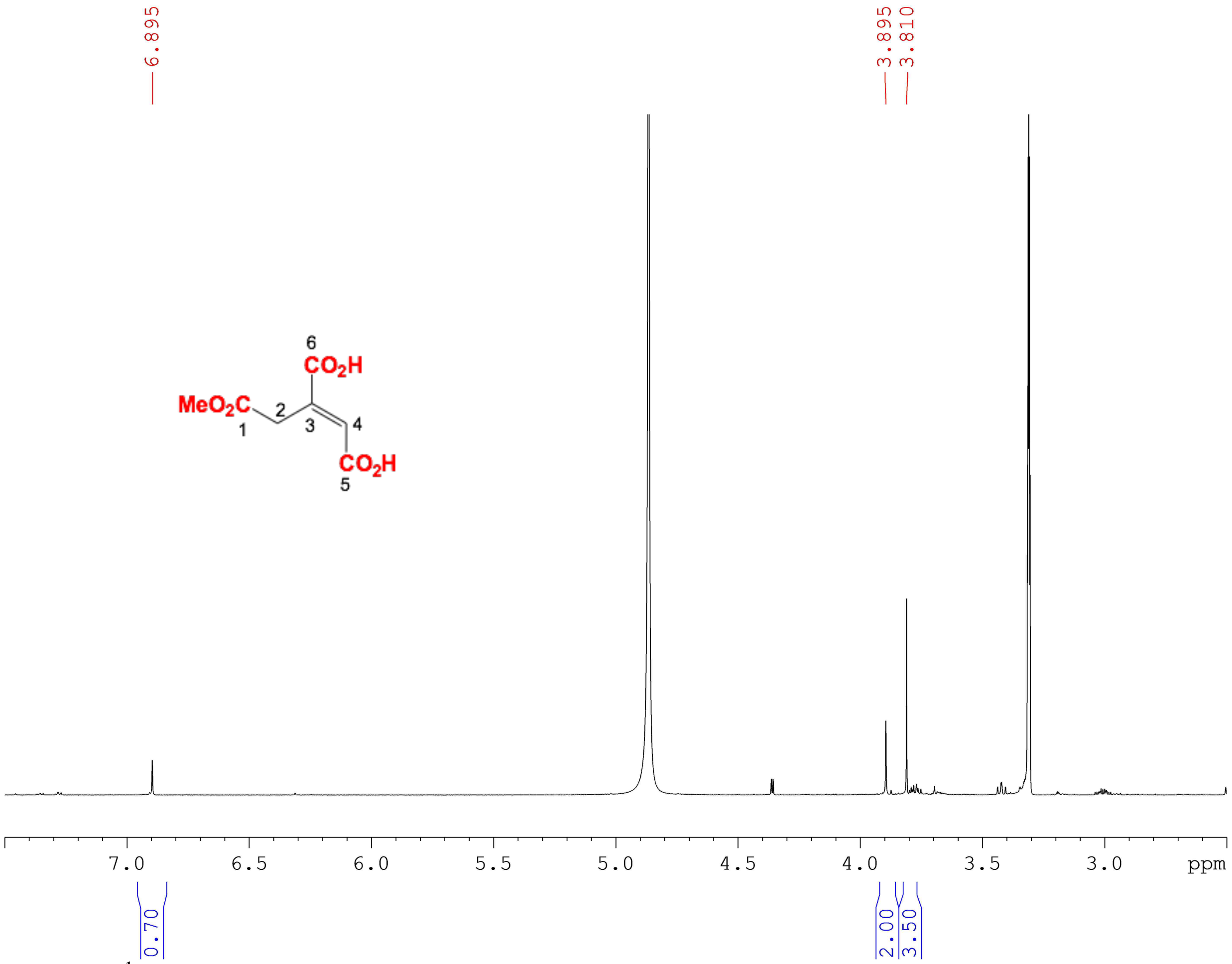

Figure S8. ^{1}H NMR (methanol-d_4) spectrum of aconitate A (**1**)

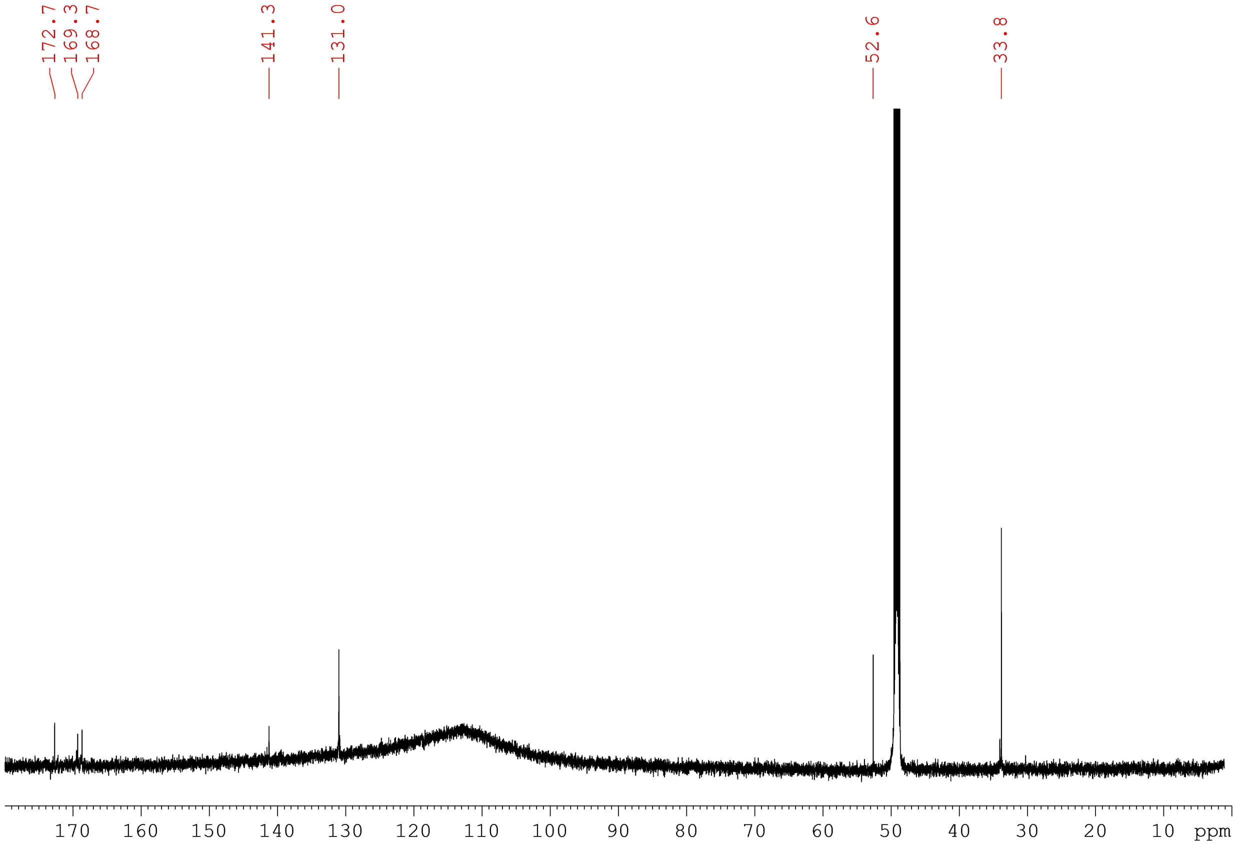

Figure S9. ^{13}C NMR (methanol-d_4) spectrum of aconitate A (**1**)

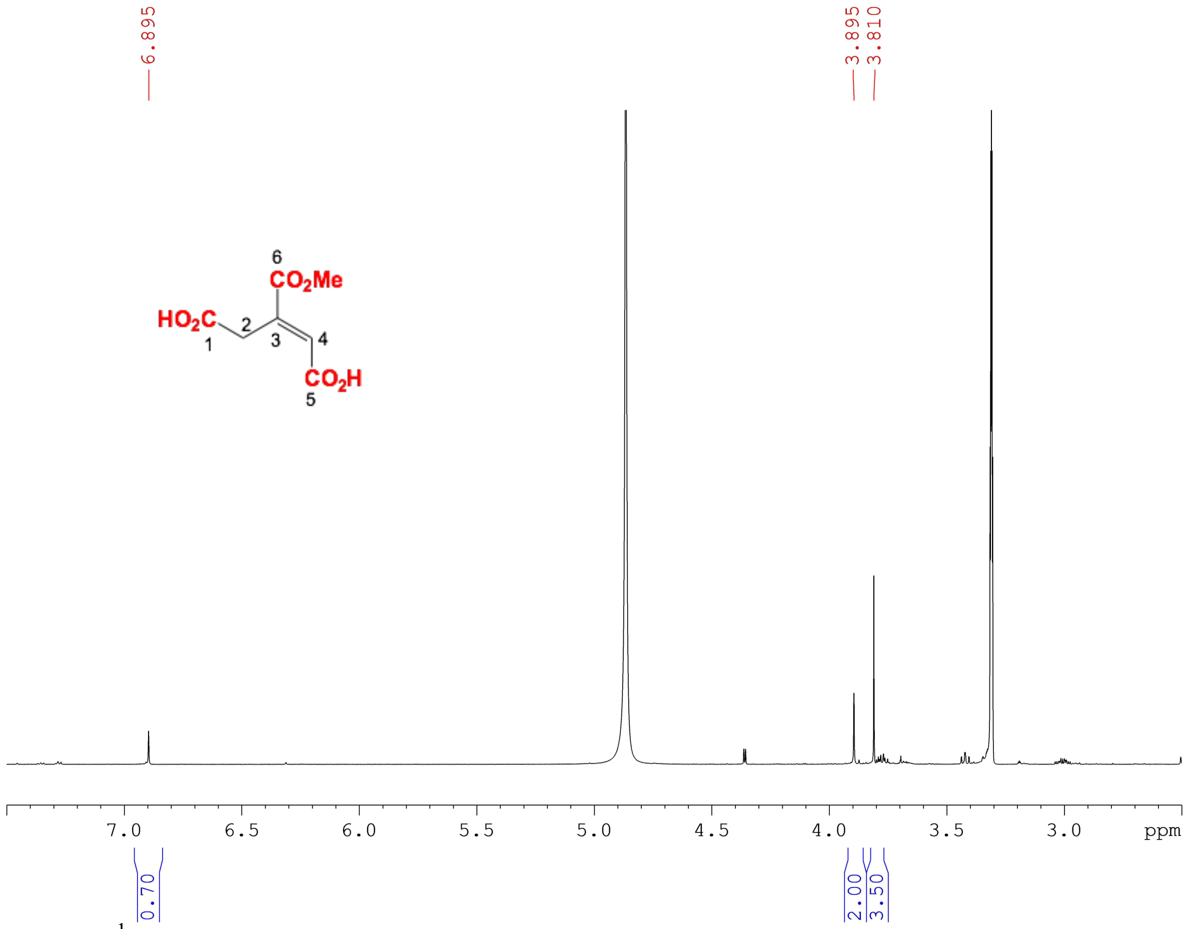

Figure S10. ^{1}H NMR (methanol-d_4) spectrum of aconitate B (**2**)

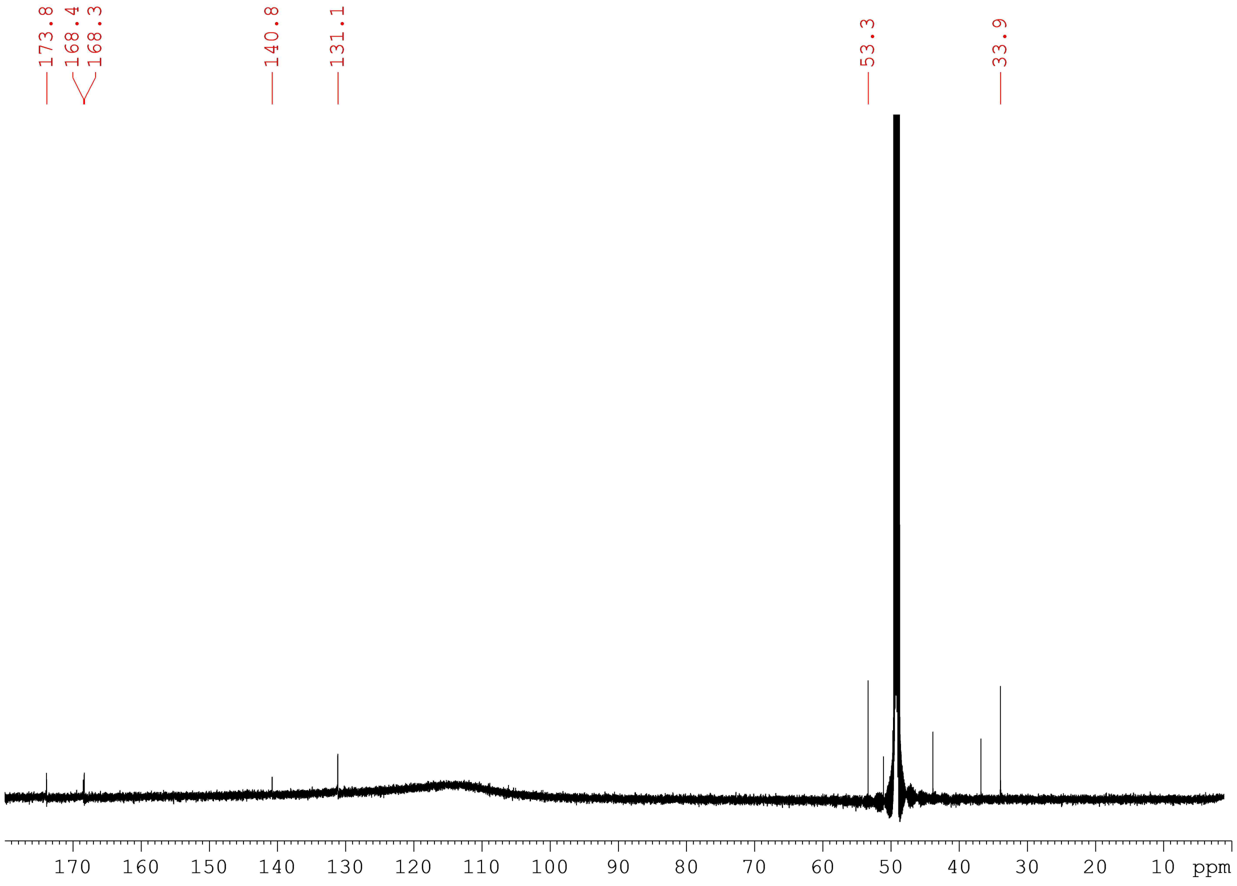

Figure S11. ^{13}C NMR (methanol-d_4) spectrum of aconitate B (**2**)

Table S2. 1D and 2D NMR (methanol-d_4) data for **1** and **2**

Pos.	aconitate A **(1)**			aconitate B **(2)**		
	$\delta_{H,}$ mult (*J* in Hz)	δ_C	HMBC	$\delta_{H,}$ mult (*J* in Hz)	δ_C	HHMBC
1		172.7			173.8	
2	3.89, s	33.8	1, 3, 4, 6	3.89, s	33.9	1, 3, 4, 6
3		141.3			140.8	
4	6.92, s	131.0	2, 3, 6	6.89, s	131.1	2, 3, 6
5		168.7			168.3	
6		169.3			168.4	
1-OCH_3	3.67, s	52.6	1			
6-OCH_3				3.81, s	53.3	6

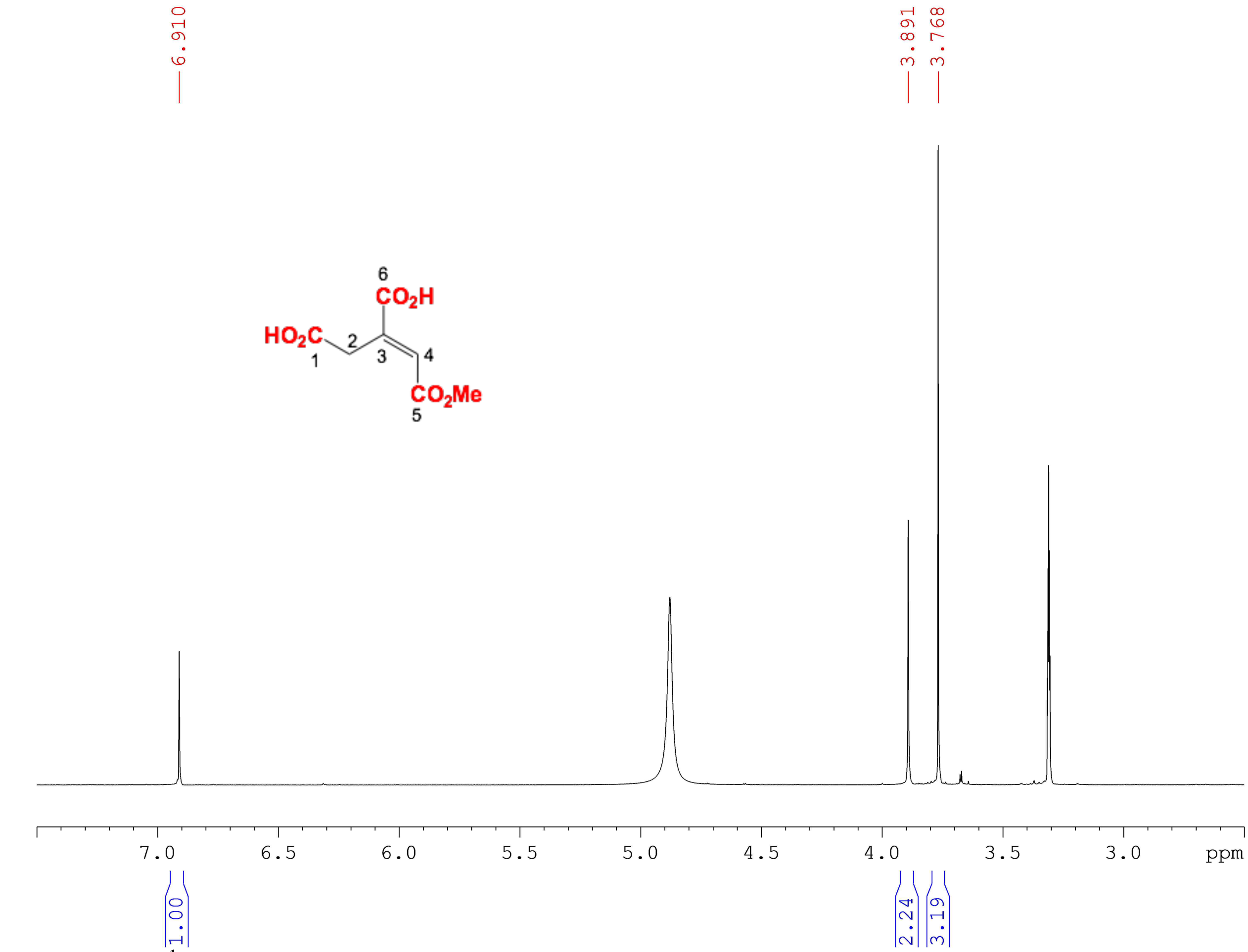

Figure S12. ^{1}H NMR (methanol-d_4) spectrum of aconitate C (**3**)

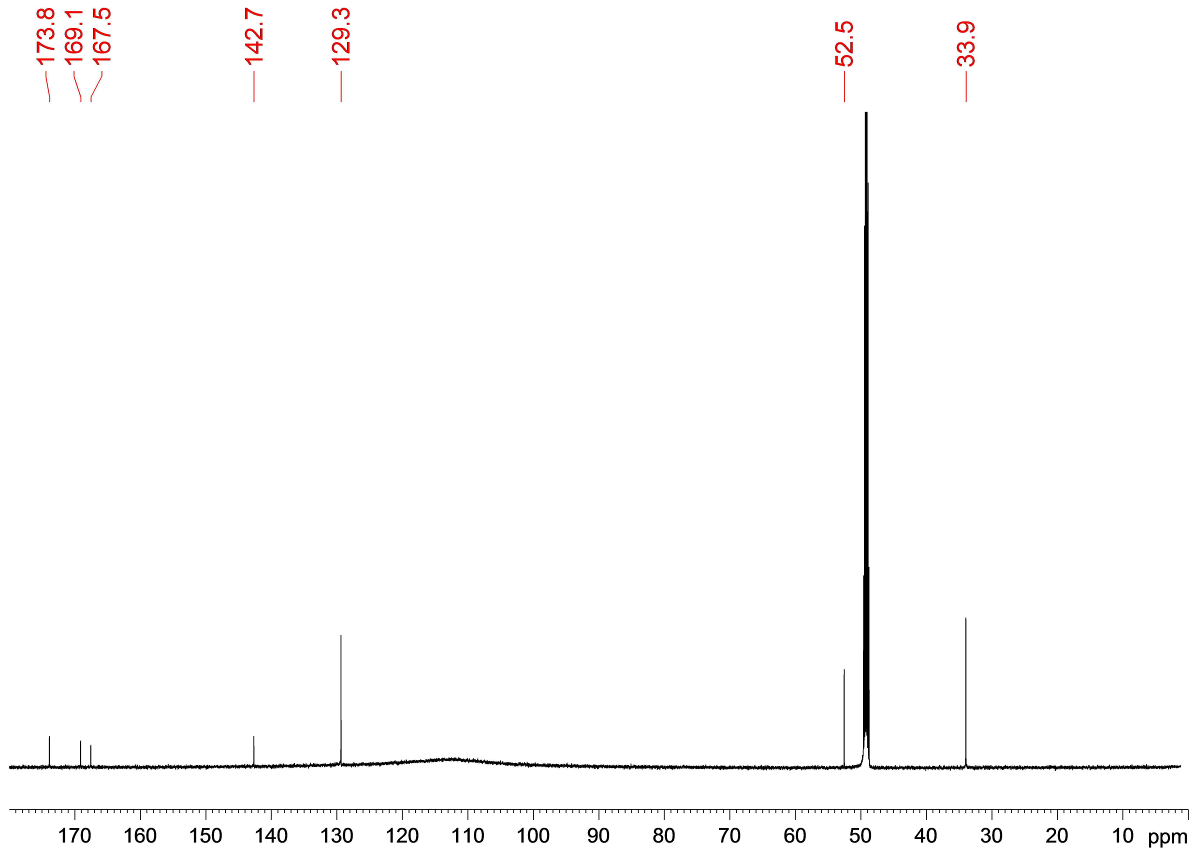

Figure S13. ^{13}C NMR (methanol-d_4) spectrum of aconitate C (**3**)

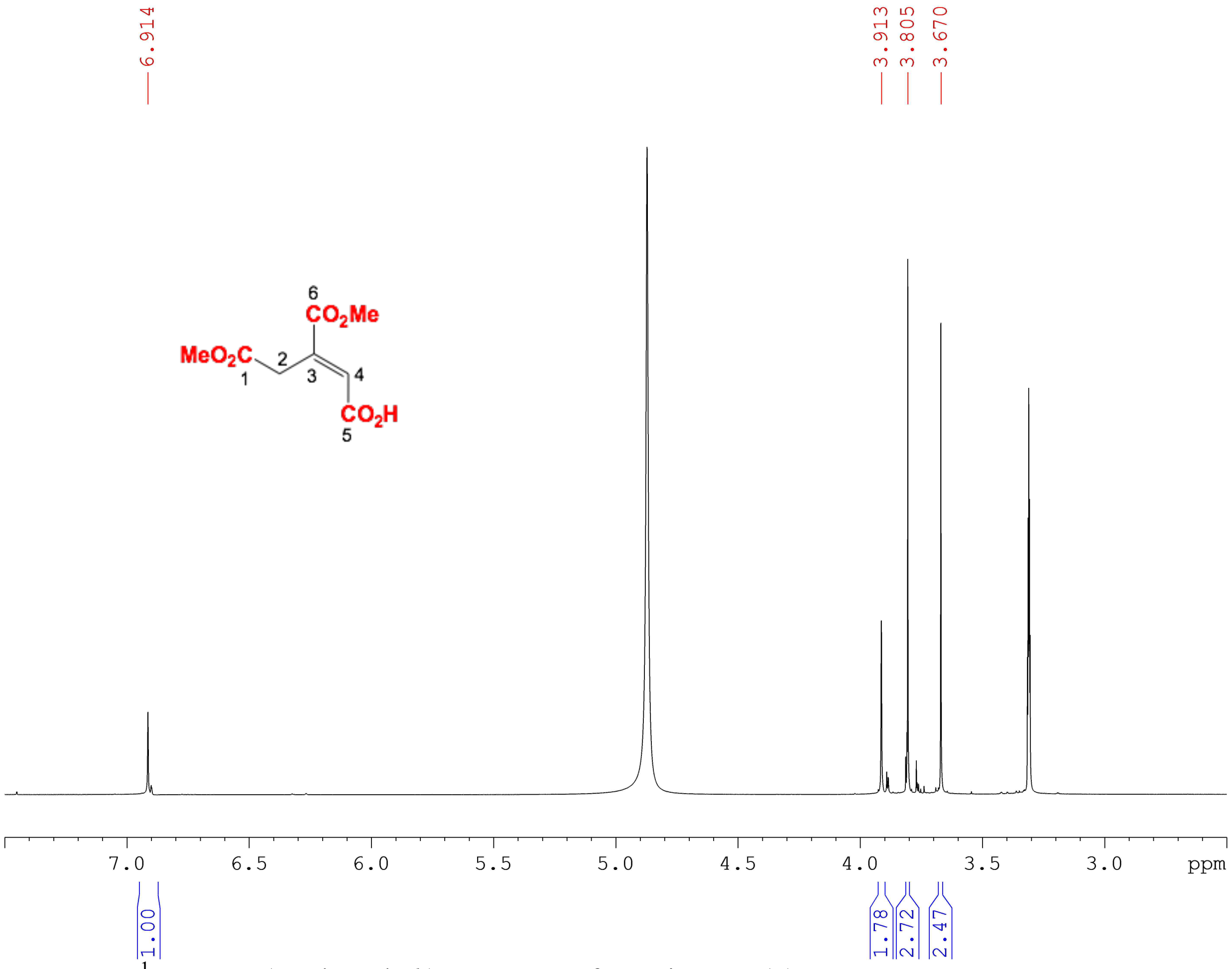

Figure S14. ^{1}H NMR (methanol-d_4) spectrum of aconitate D (**4**)

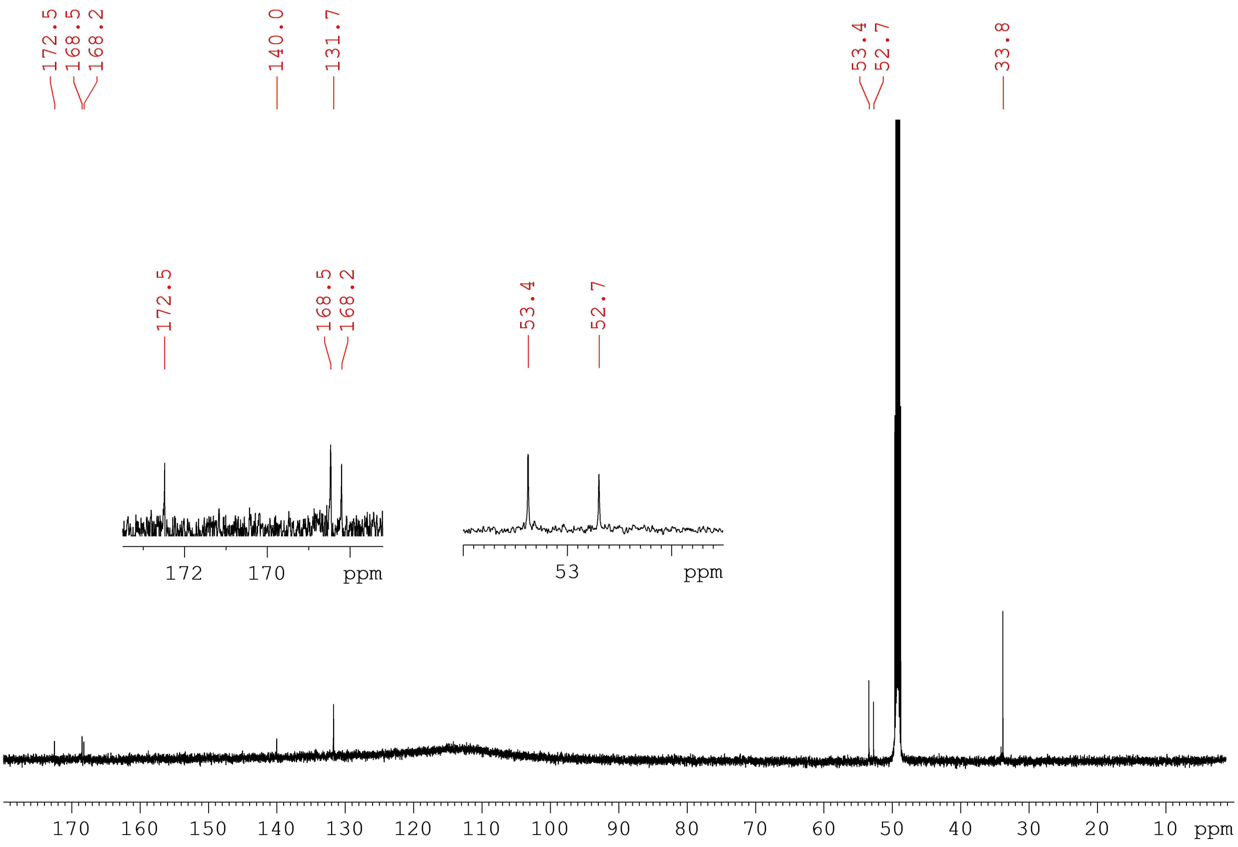

Figure S15. ^{13}C NMR (methanol-d_4) spectrum of aconitate D (**4**)

Table S3. 1D and 2D NMR (methanol-d_4) data for **3** and **4**

Pos.	aconitate C (**3**)			aconitate D (**4**)		
	$\delta_{H,}$ mult (*J* in Hz)	δ_C	HMBC	$\delta_{H,}$ mult (*J* in Hz)	δ_C	HMBC
1		173.8			172.5	
2	3.89, s	33.9	1, 3, 4, 6	3.91, s	33.8	1, 3, 4, 6
3		142.7			140.0	
4	6.91, s	129.3	2, 3, 5, 6	6.91, s	131.7	2, 3, 6
5		167.5			168.5	
6		169.1			168.2	
1-OCH_3				3.67, s	52.7	1
5-OCH_3	3.77, s	52.5	5			
6-OCH_3				3.80, s	53.4	6

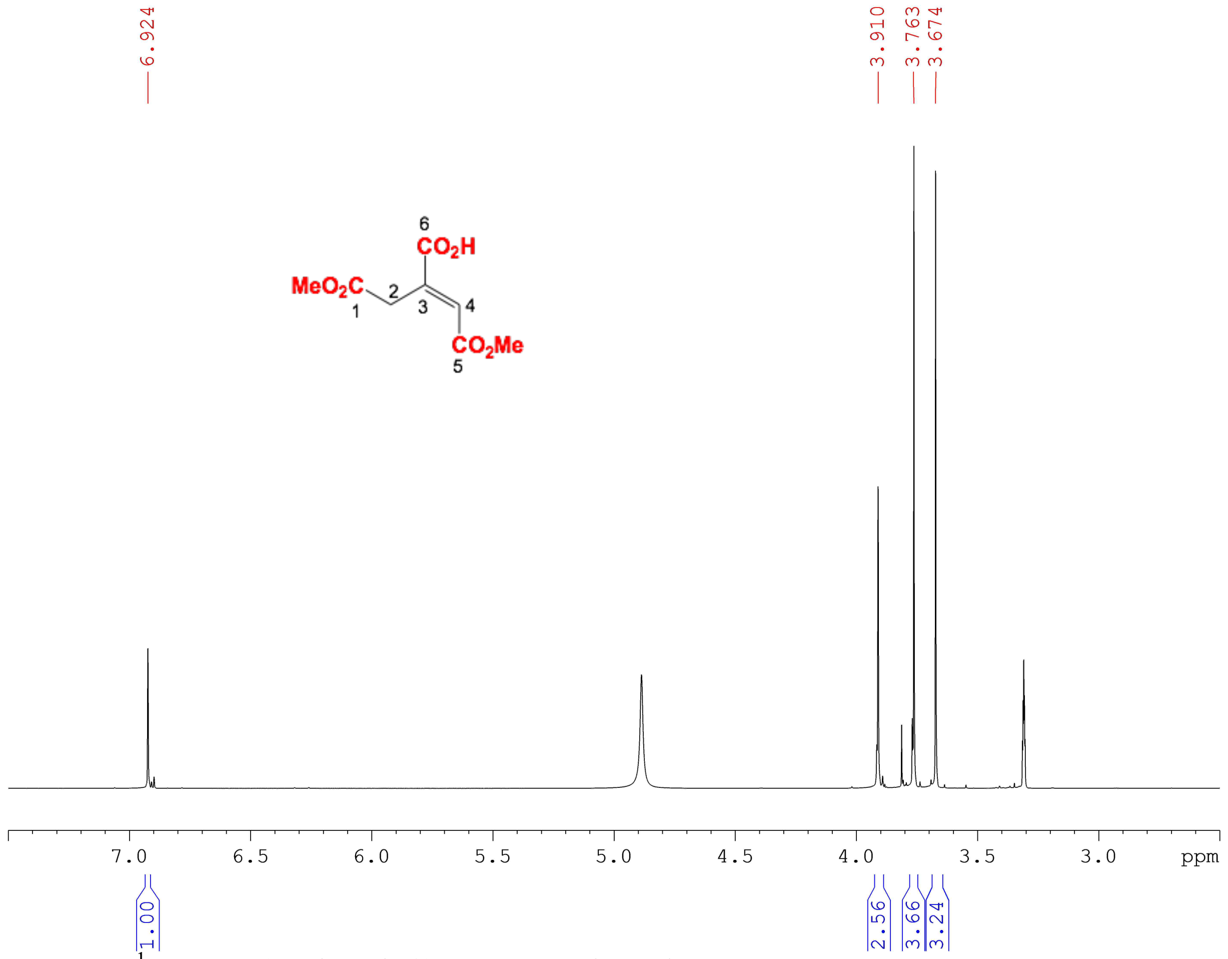

Figure S16. ^{1}H NMR (methanol-d_4) spectrum of aconitate E **(5)**

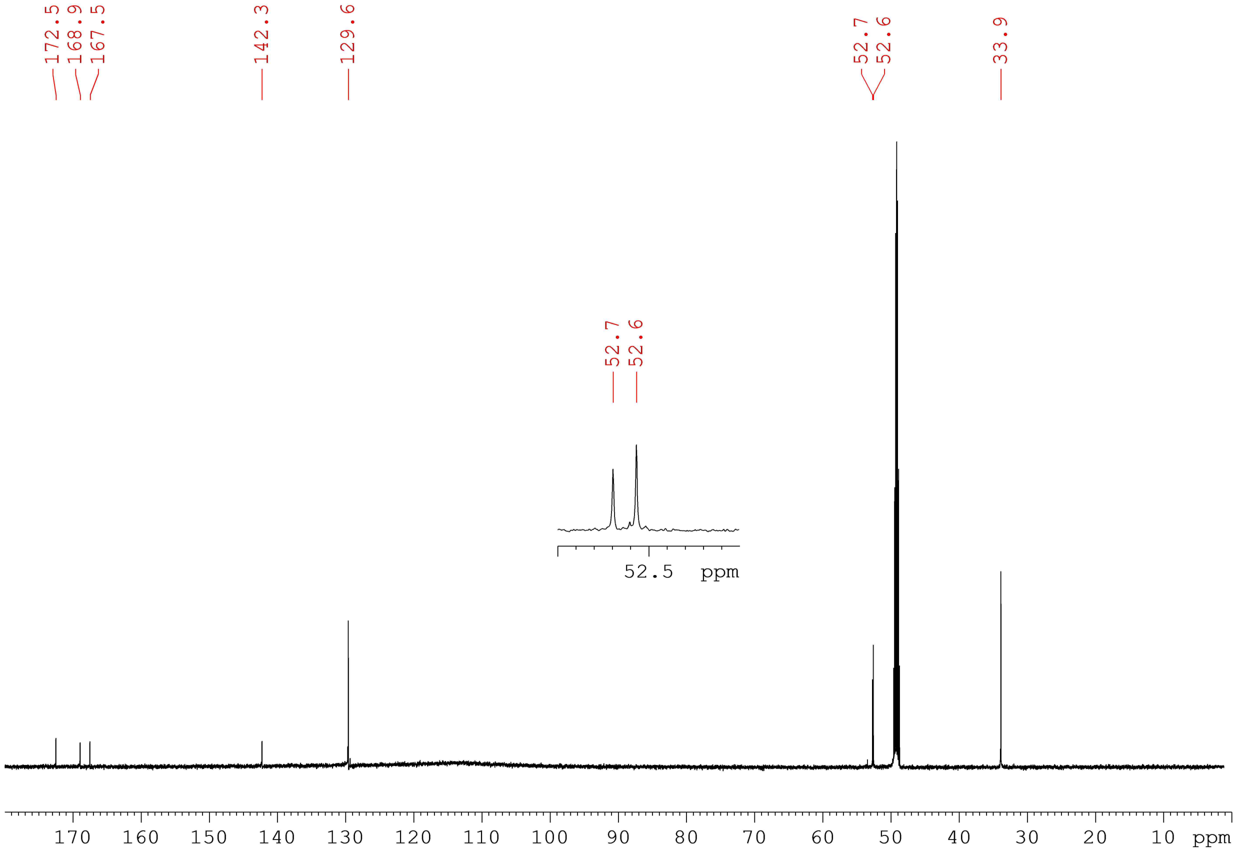

Figure S17. ^{13}C NMR (methanol-d_4) spectrum of aconitate E **(5)**

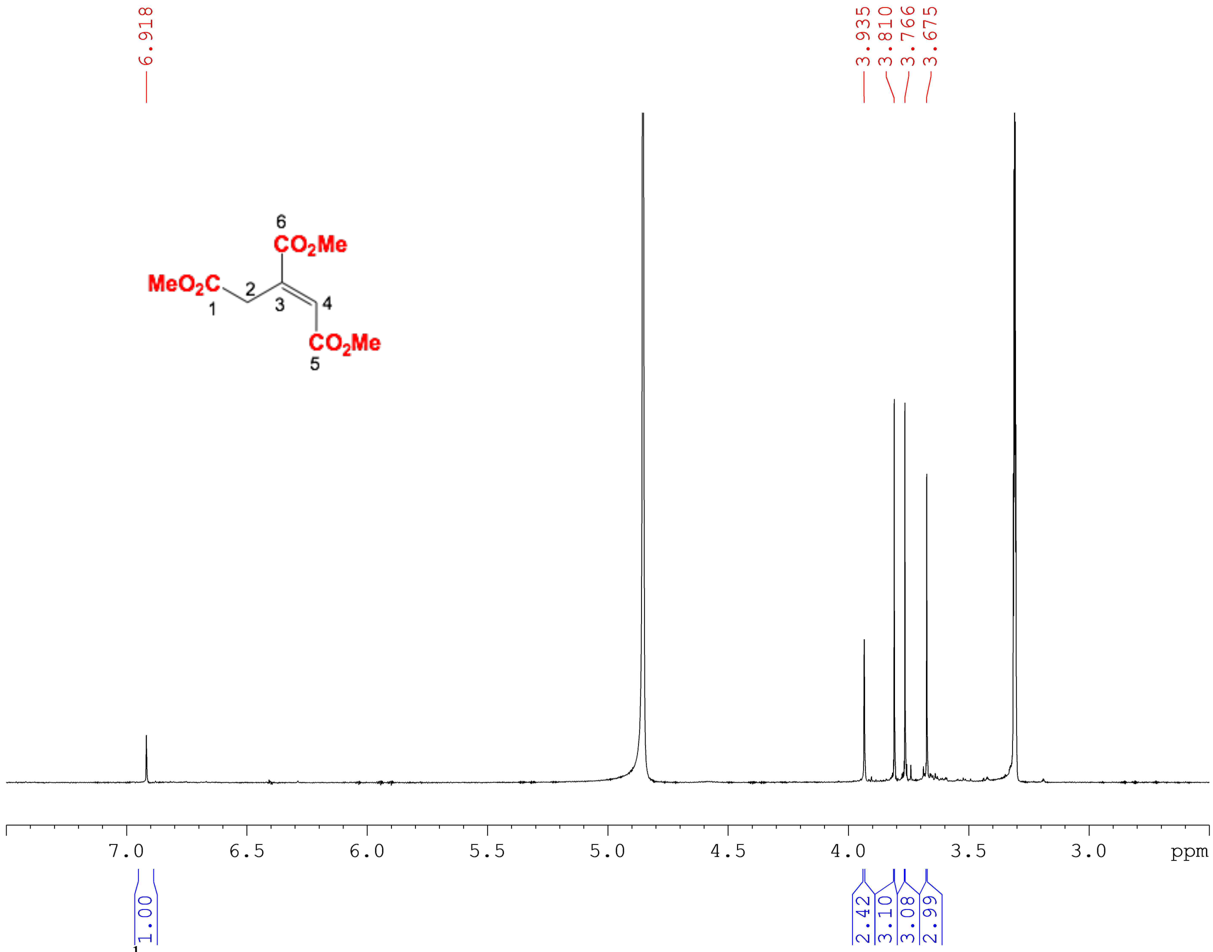

Figure S18. ^{1}H NMR (methanol-d_4) spectrum of aconitate F **(6)**

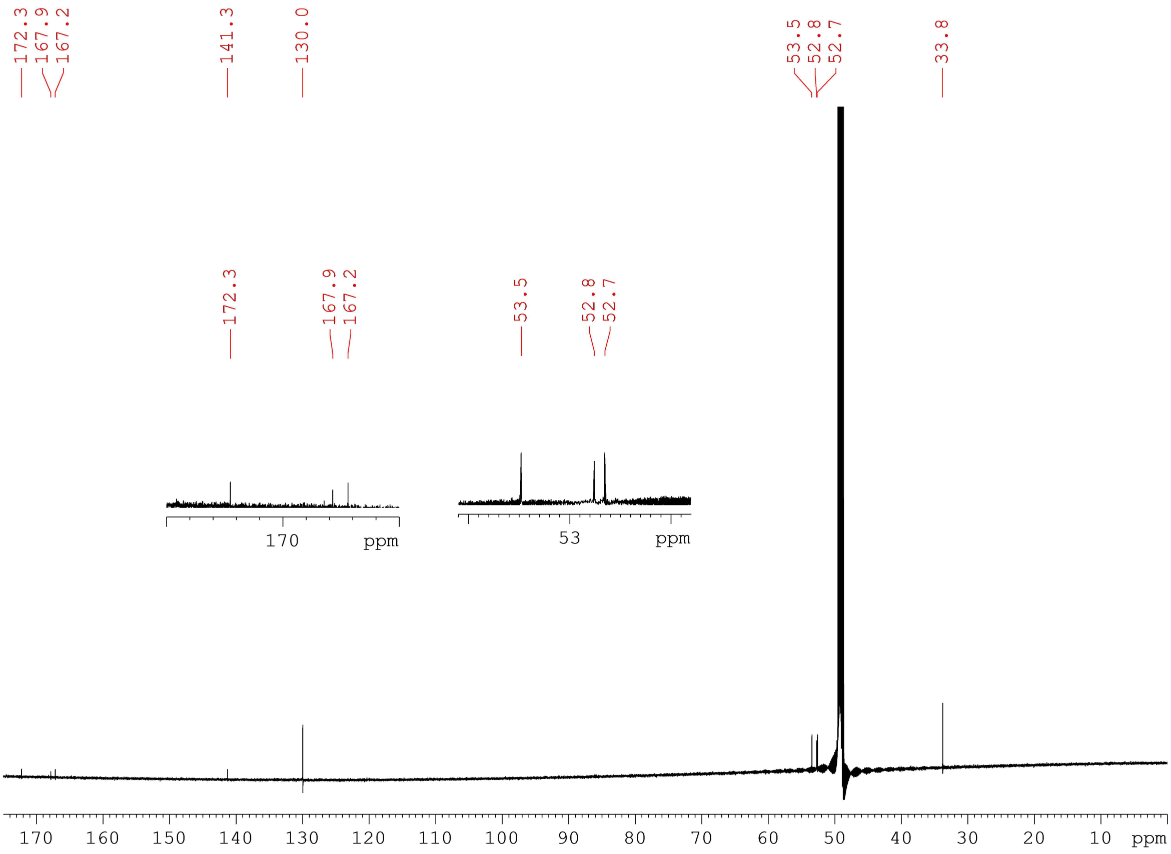

Figure S19. ^{13}C NMR (methanol-d_4) spectrum of aconitate F **(6)**

Table S4. 1D and 2D NMR (methanol-d_4) data for **5** and **6**

Pos.	aconitate E (**5**)			aconitate E **(6)**		
	$\delta_{H,}$ mult (*J* in Hz)	δ_C	HMBC	$\delta_{H,}$ mult (*J* in Hz)	δ_C	HMBC
1		172.5			172.2	
2	3.91, s	33.9	1, 3, 4, 6	3.93, s	33.8	1, 3, 4, 6
3		142.3			141.3	
4	6.92, s	129.6	2, 3, 5, 6	6.92, s	130.0	2, 3, 6
5		167.5			167.2	
6		168.9			167.8	
1-OCH_3	3.67, s	52.7	1	3.67, s	52.8	1
5-OCH_3	3.76, s	52.6	5	3.76, s	52.7	5
6-OCH_3				3.81, s	53.5	6

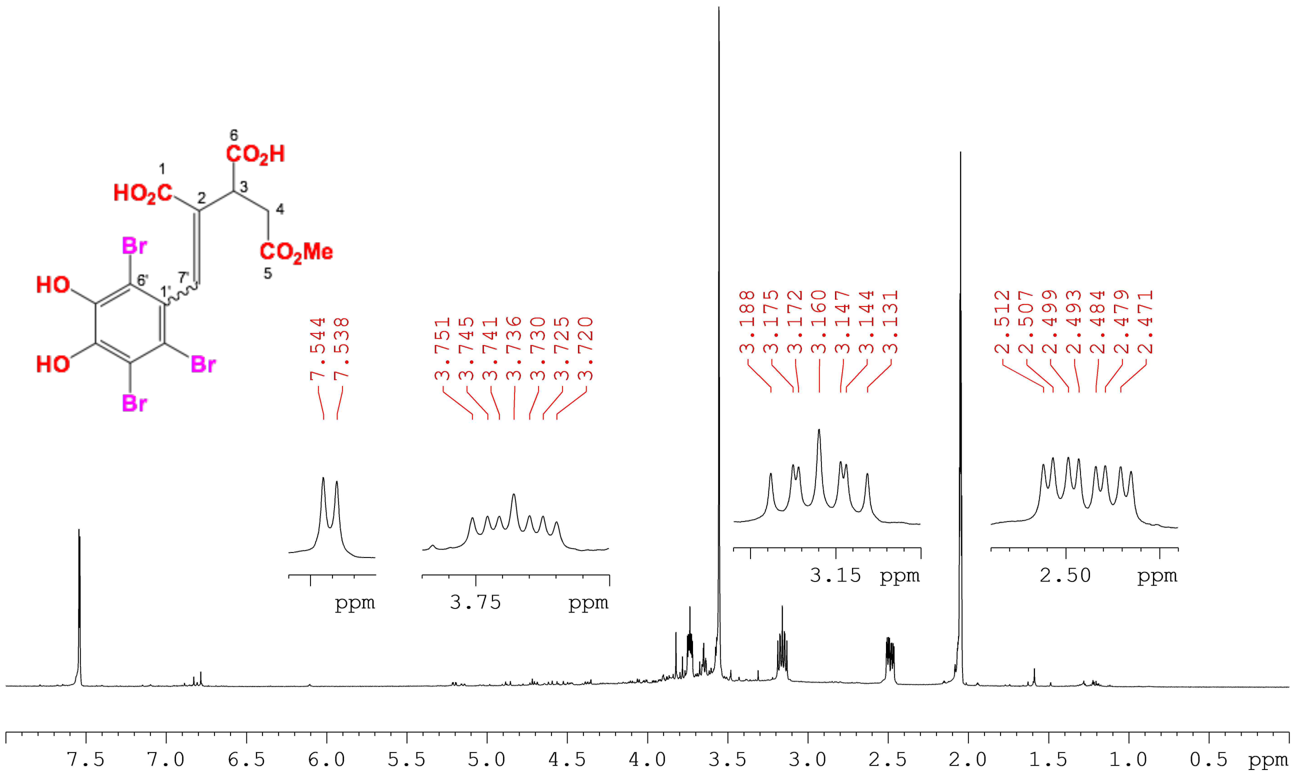

Figure S20. ^{1}H NMR (acetone-d_6) spectrum of symphyocladins C/D **(7a/b)**

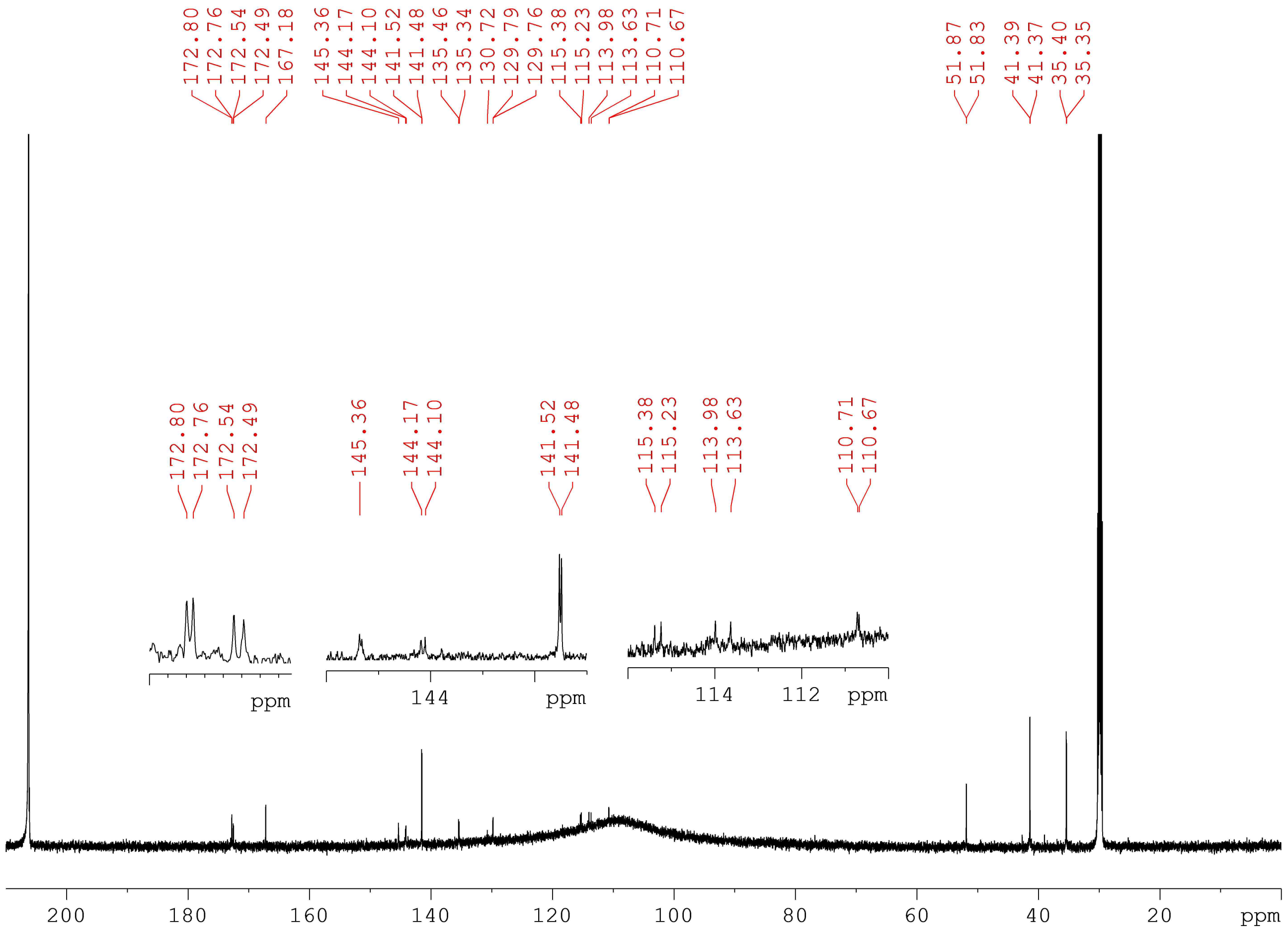

Figure S21. ^{13}C NMR (acetone-d_6) spectrum of symphyocladins C/D **(7a/b)**

Table S5. 1D and 2D NMR data (600 MHz, acetone-d_6) of symphyocladins C/D **(7a/b)**

pos	$\delta_{H,}$ mult (*J* in Hz)	δ_C	COSY	HMBC	ROESY
1		167.18			
2		135.46/135.34			
3	3.74, m	41.39/41.37	4a, 4b	1, 2, 4, 5, 6, 7′	7′
4a	3.17, m	35.40/35.35	3, 4b	2, 3, 5, 6,	7′
4b	2.490/2.489, dd *(16.8, 7.8)*		3, 4a	2, 3, 5, 6,	7′
5[a]		172.80/172.76			
6[a]		172.54/172.49			
5-OCH_3	3.55, s	51.87/51.83		5	
1′		129.79/129.76			
2′		115.38/115.23			
3′		113.98/113.63			
4′[b]		144.17/144.10			
5′[b]		145.36/145.32			
6′		110.71/110.67			
7′	7.544/7.538, s	141.52/141.48		1, 2, 3, 1′, 2′, 6′	3, 4a, 4b

[a-b] assignments are interchangeable within the same letter.

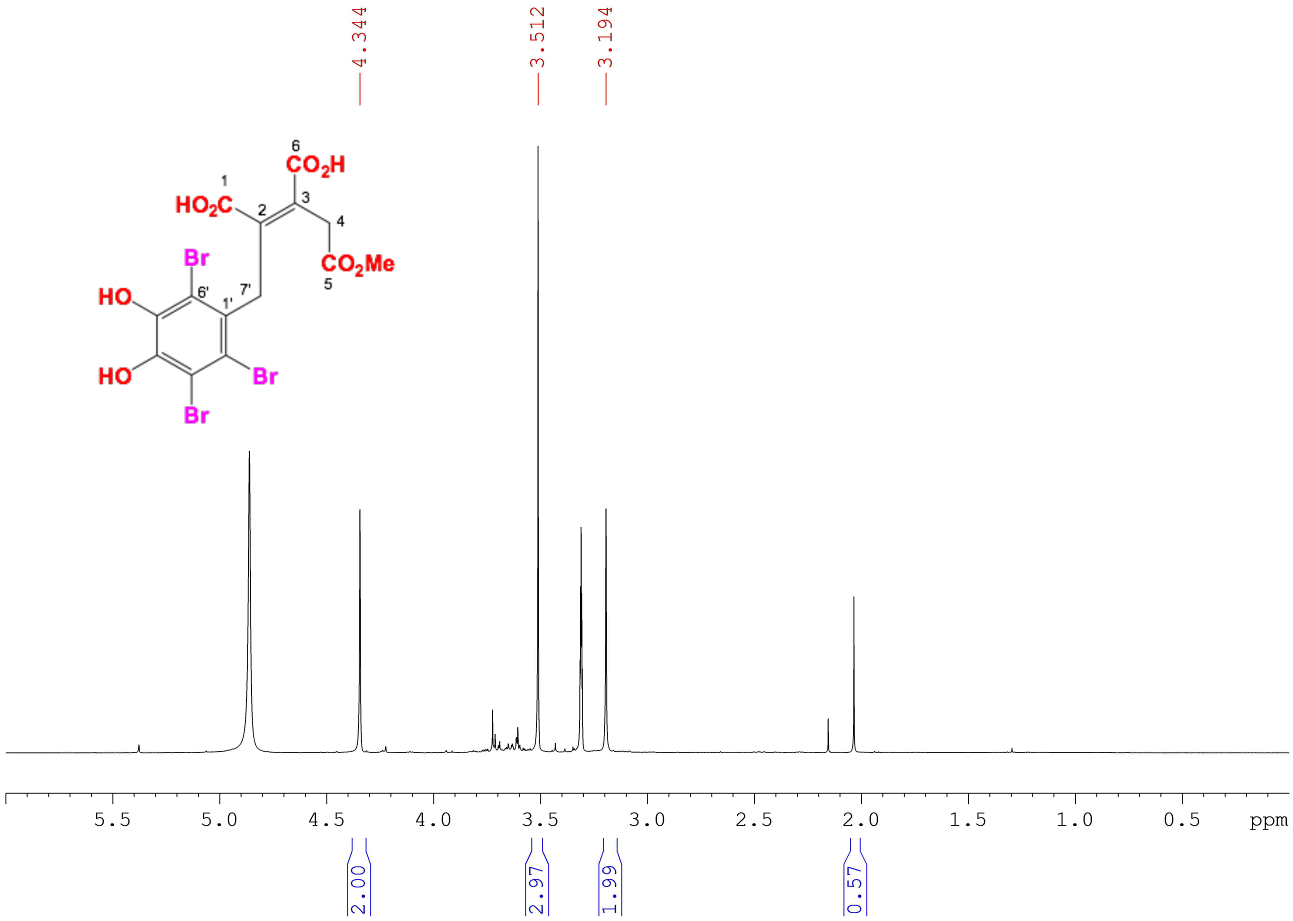

Figure S22. ^{1}H NMR (methanol-d_4) spectrum of symphyocladins H/I **(8a/b)** (prior to equilibration)

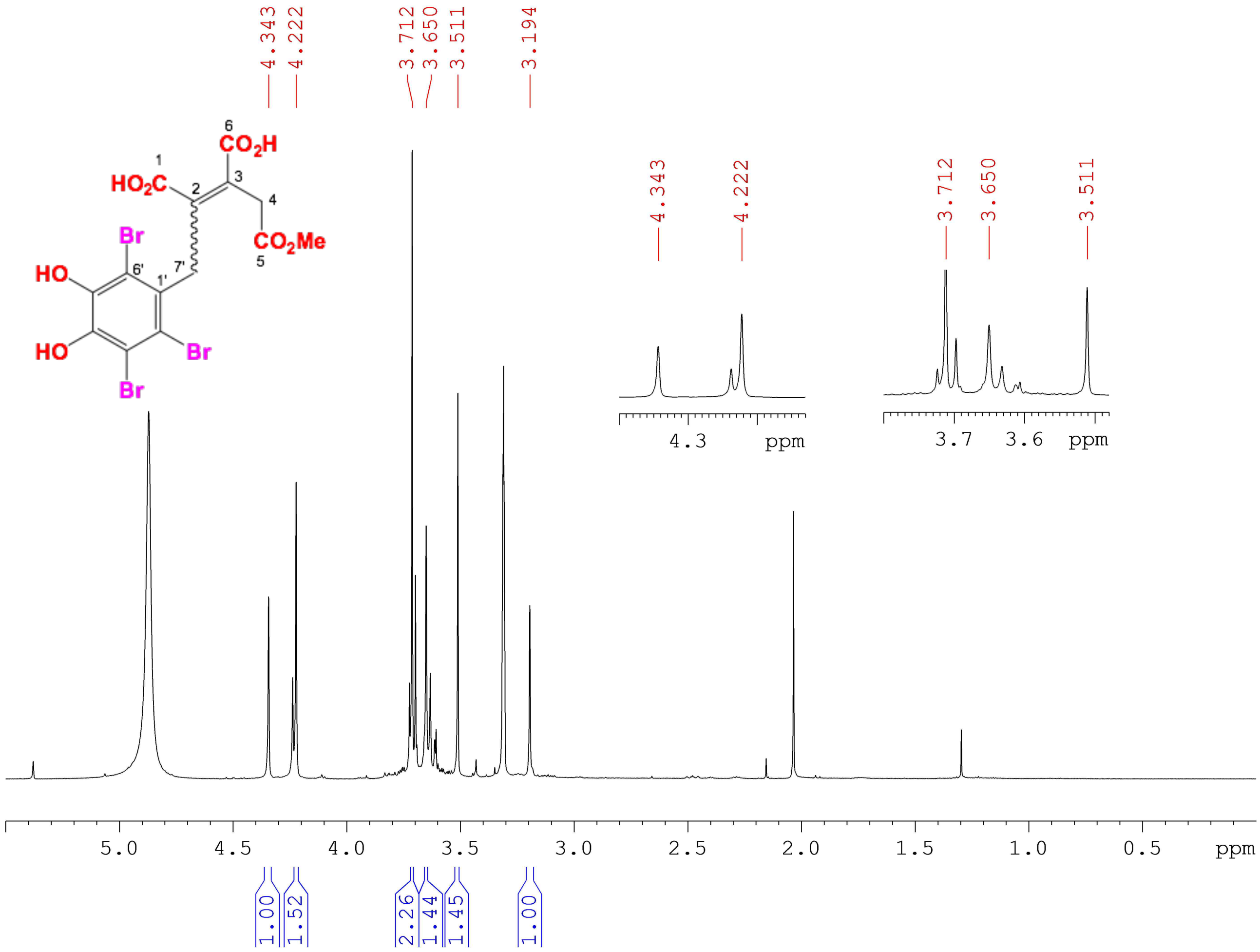

Figure S23. ^{1}H NMR (methanol-d_4) spectrum of symphyocladins H/I **(8a/b)** (after overnight storage, with $\Delta^{2,3}$ equilibration of a mixture of *E* and *Z* isomers)

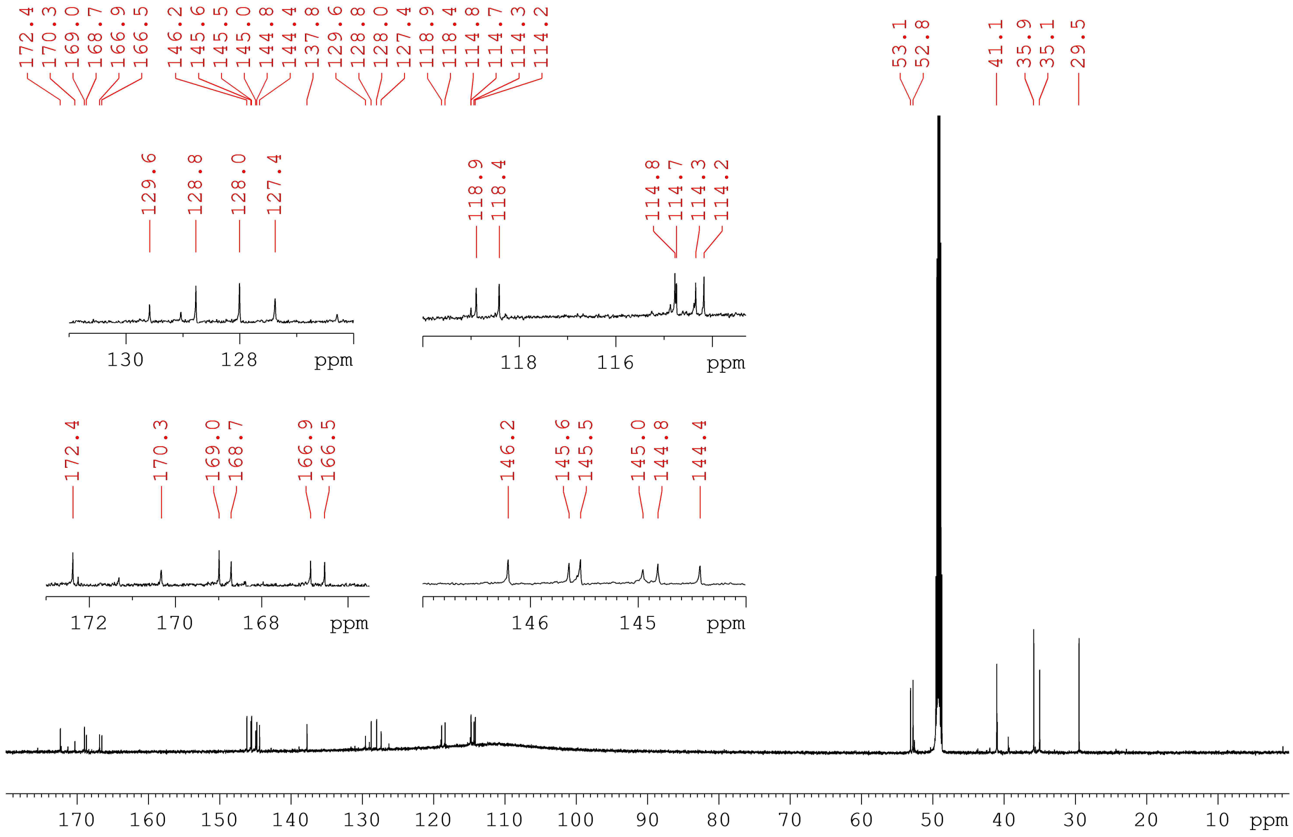

Figure S24. ^{13}C NMR (methanol-d_4) spectrum of symphyocladins H/I **(8a/b)**

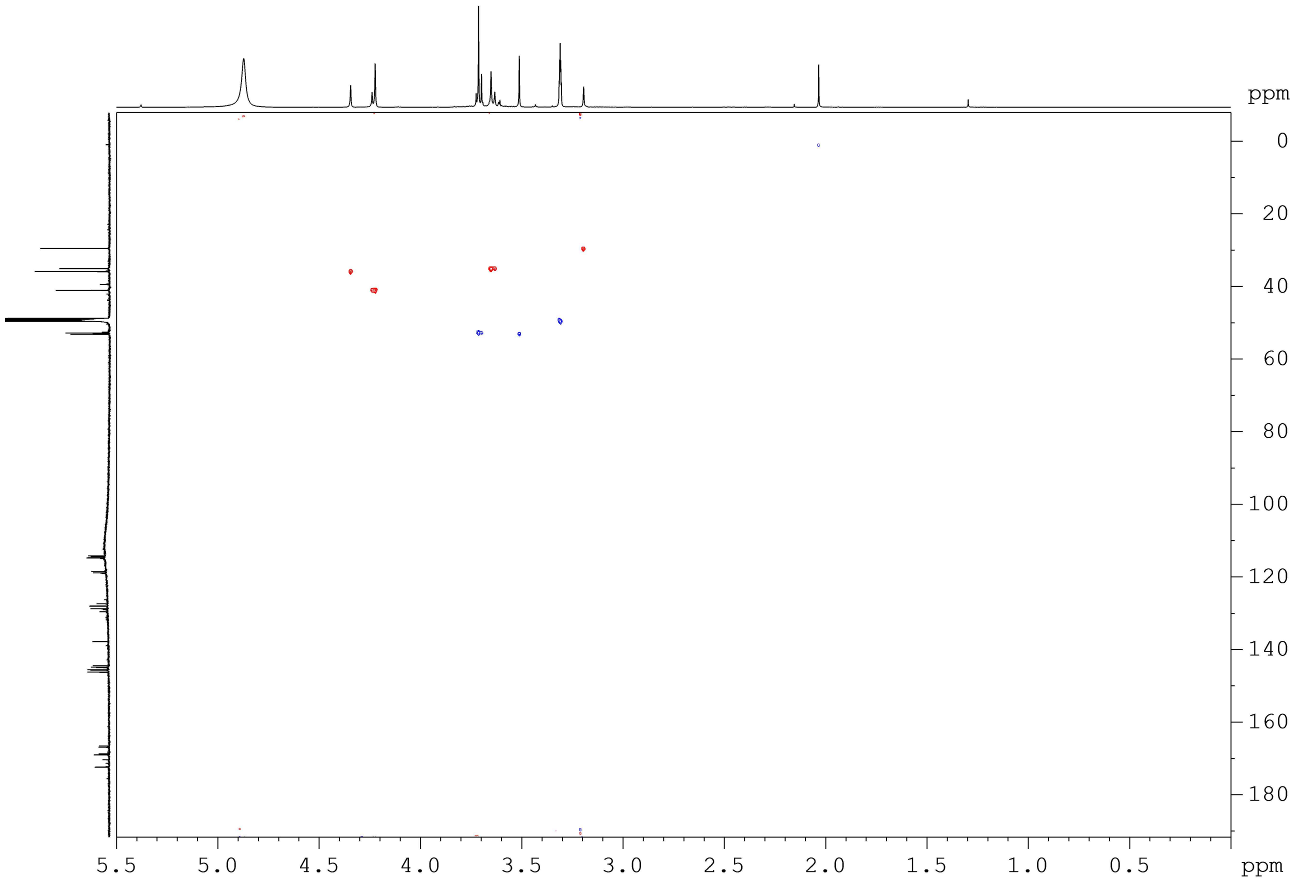

Figure S25. HSQC (methanol-d_4) spectrum of symphyocladins H/I **(8a/b)**

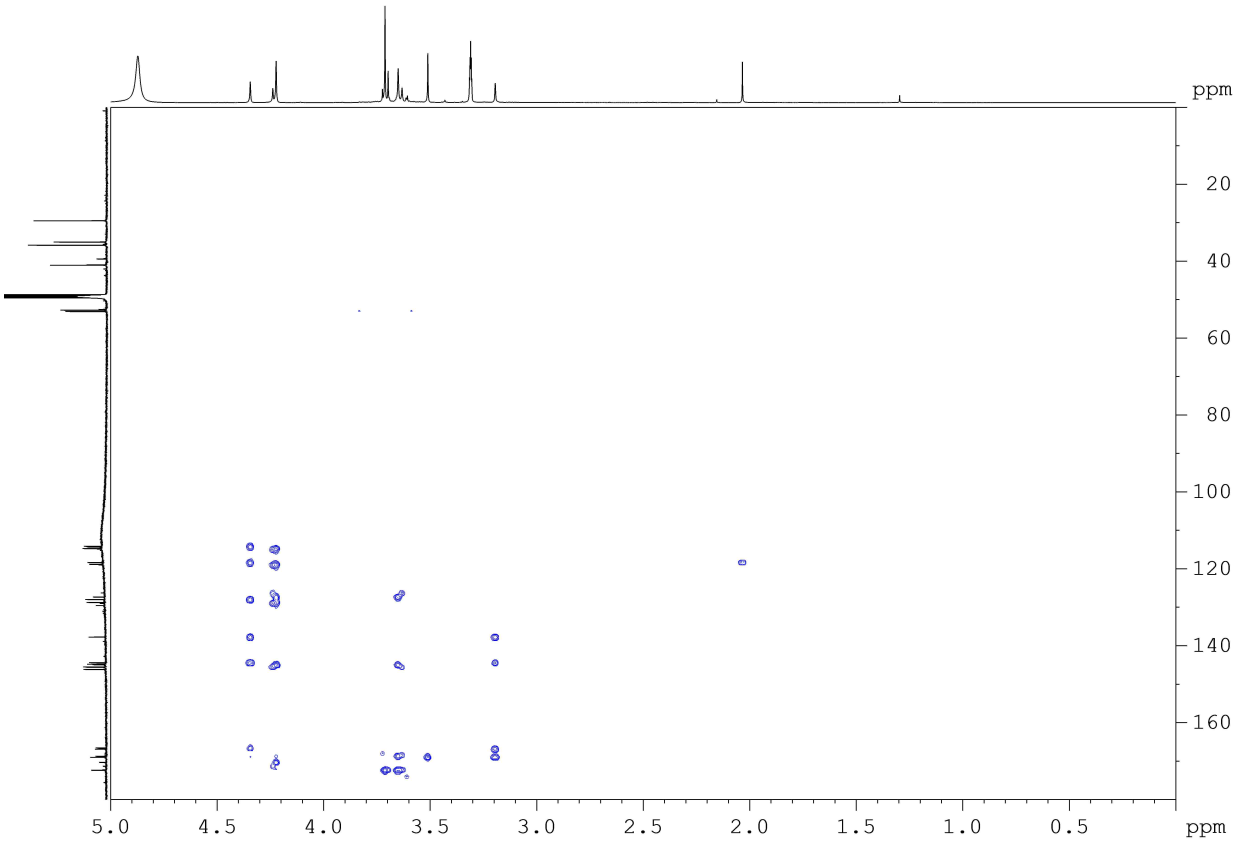

Figure S26. HMBC (methanol-d_4) spectrum of symphyocladins H/I **(8a/b)**

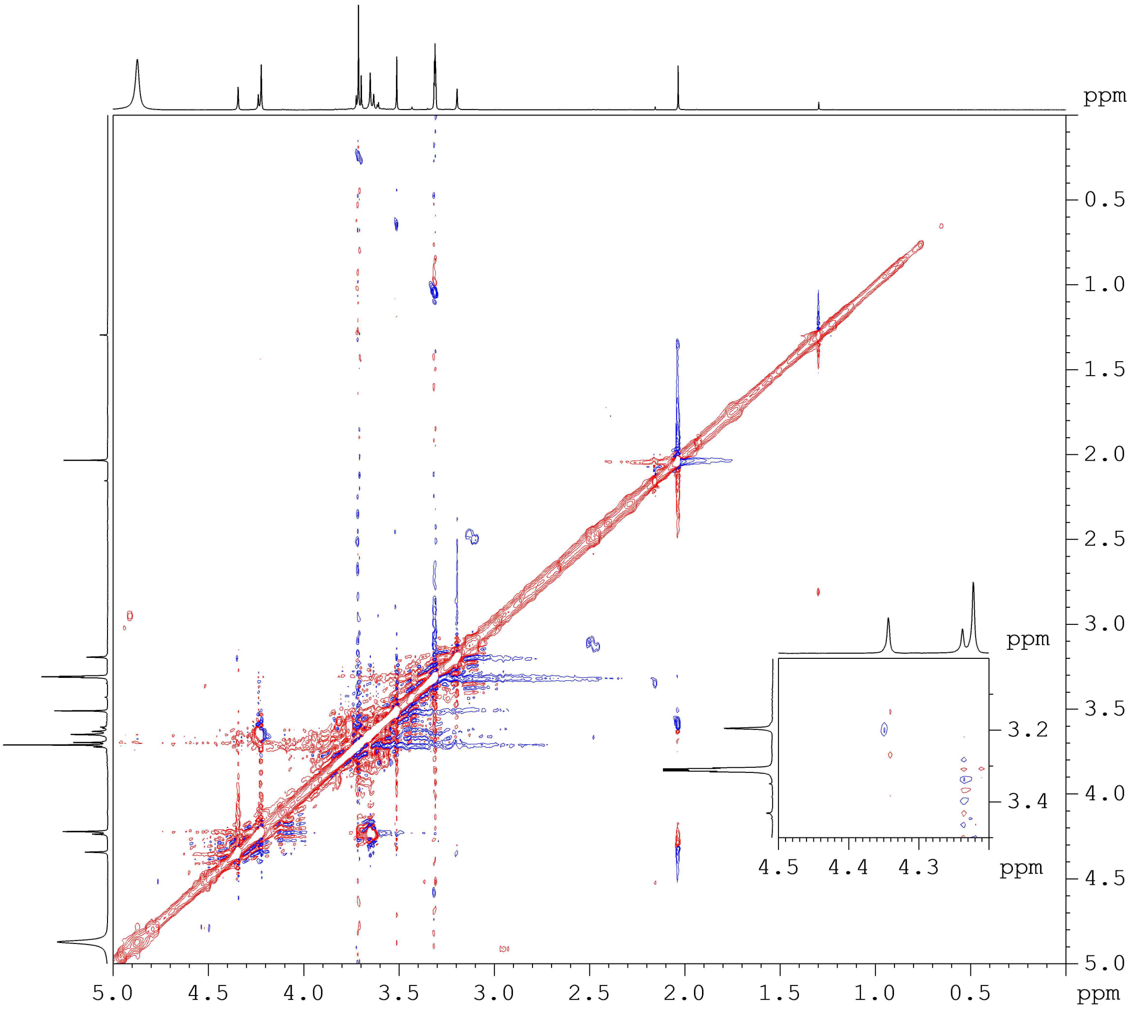

Figure S27. ROESY (methanol-d_4) spectrum of symphyocladins H/I **(8a/b)**

Table S6. 1D and 2D NMR data (600 MHz, methanol-d_4) of symphyocladins H/I **(8a/b)**

pos	major (*E*)			minor (*Z*)			
	$\delta_{H,}$ mult (*J* in Hz)	δ_C	HMBC	$\delta_{H,}$ mult (*J* in Hz)		HMBC	ROESY
1		170.3			166.5		
2		145.0			144.4		
3		127.4			137.8		
4	3.65, s	35.0	2, 3, 5, 6	3.19, s	29.5	2, 3, 5, 6	7′
5		168.7			166.8		
6		172.4			169.0		
5-OCH_3	3.71, s	52.8	5	3.51, s	53.1	5	
1′		128.8			127.9		
2′		118.9			118.4		
3′		114.8			114.2		
4′[a]		146.2			145.5		
5′[a]		145.6			144.8		
6′		114.7			114.3		
7′	4.22, s	41.1	1, 2, 3, 1′, 2′, 6′	4.34, s	35.9	1, 2, 3, 1′, 2′, 6′	4

[a]assignments are interchangeable

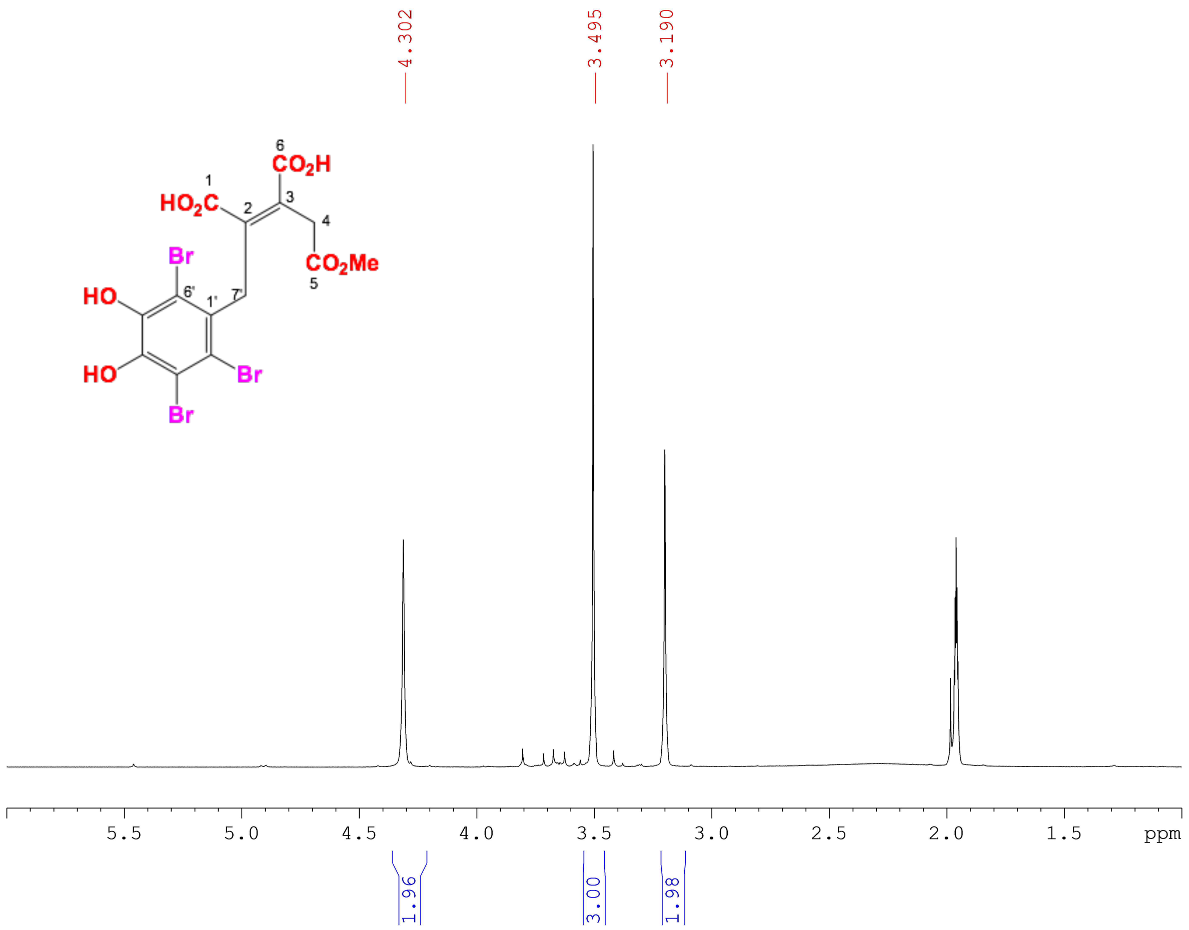

Figure S28. ^{1}H NMR (acetonitrile-d_3) spectrum of symphyocladin H **(8a)**

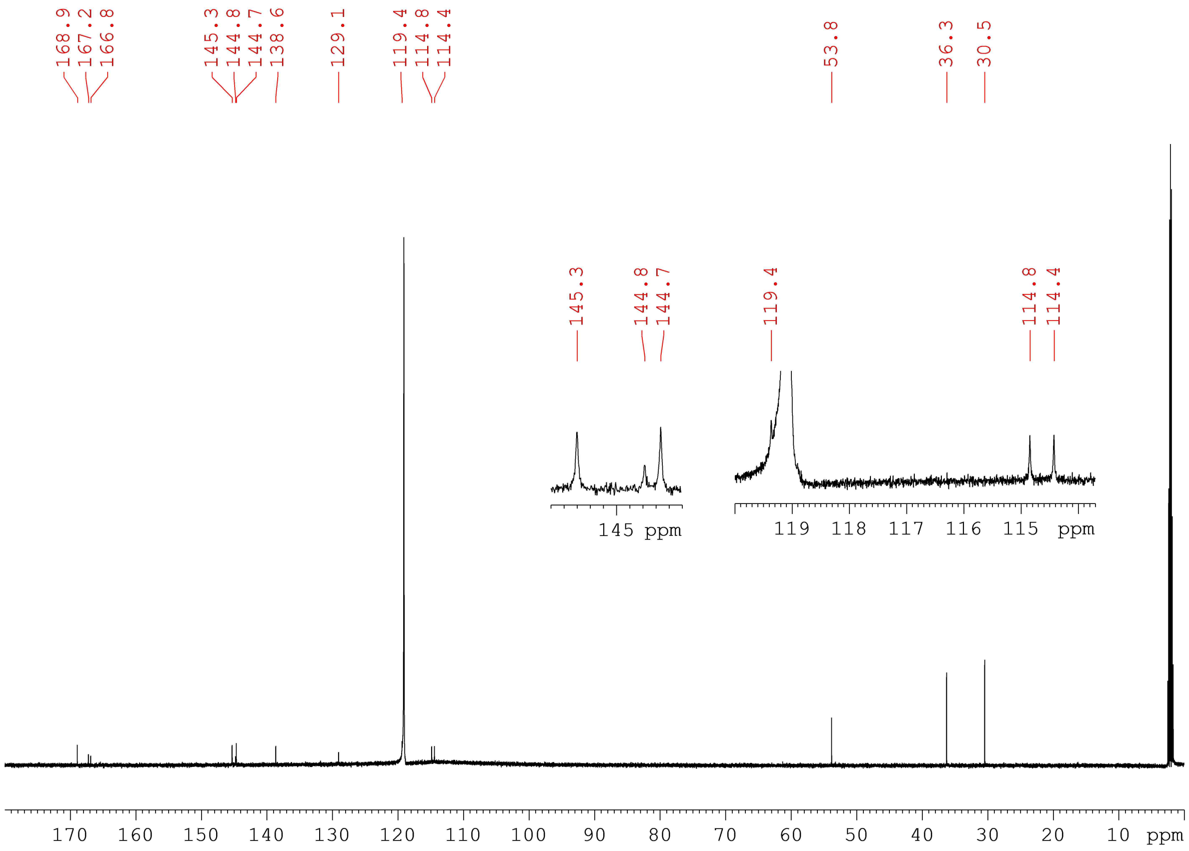

Figure S29. ^{13}C NMR (acetonitrile-d_3) spectrum of symphyocladin H **(8a)**

Table S7. 1D and 2D NMR data (600 MHz, acetonitrile-d_3) of symphyocladin H **(8a)**

pos	$\delta_{H,}$ mult (*J* in Hz)	δ_C	HMBC	ROESY
1		166.8		
2		144.8		
3		138.6		
4	3.19, s	30.5	2, 3, 5, 6	7′
5		168.9		
6		167.2		
5-OCH_3	3.49, s	53.8	5	
1′		129.1		
2′		119.4		
3′		114.8		
4′[a]		145.3		
5′[a]		144.7		
6′		114.4		
7′	4.30, s	36.3	1, 2, 3, 1′, 2′, 6′	4

[a] assignments are interchangeable

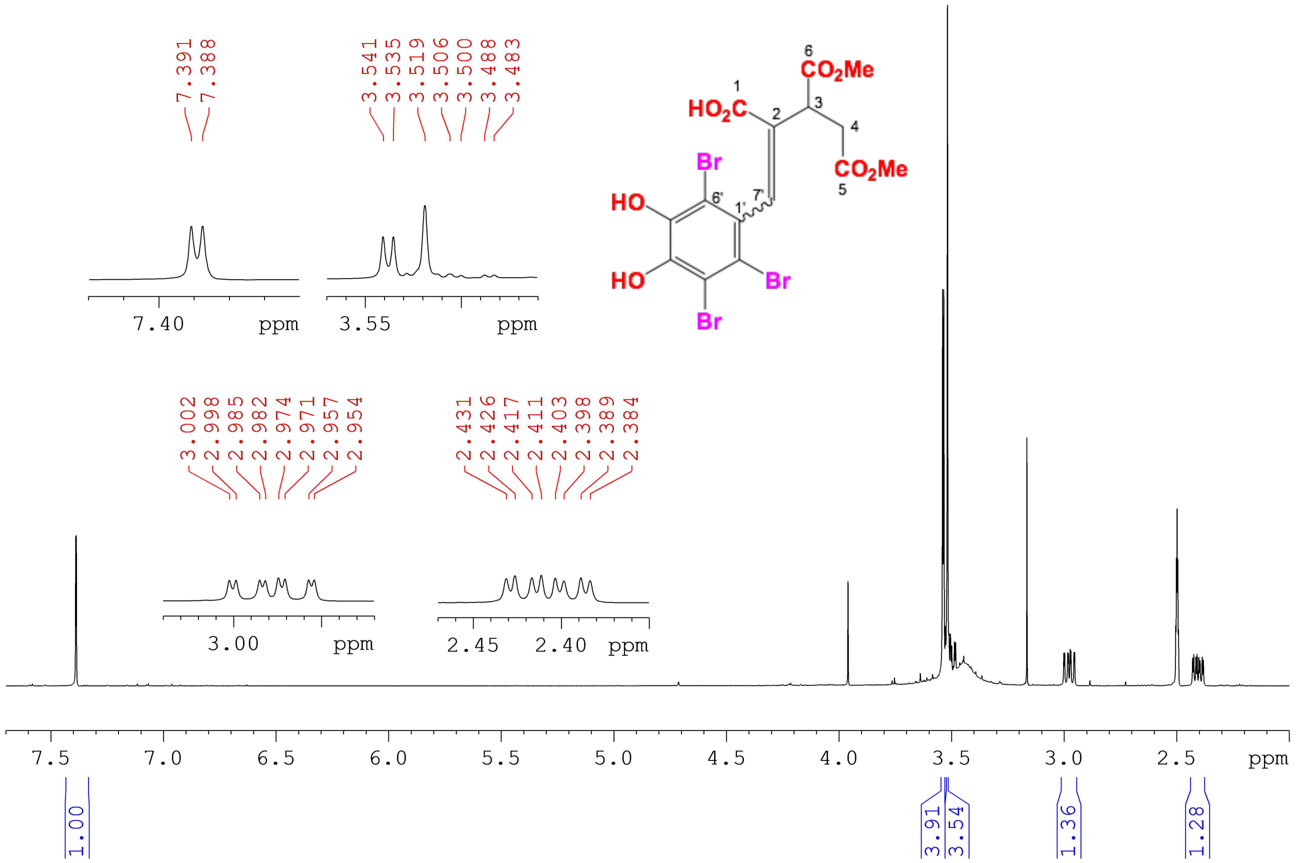

Figure S30. ^{1}H NMR (DMSO-d_6) spectrum of symphyocladins J/K **(9a/b)**

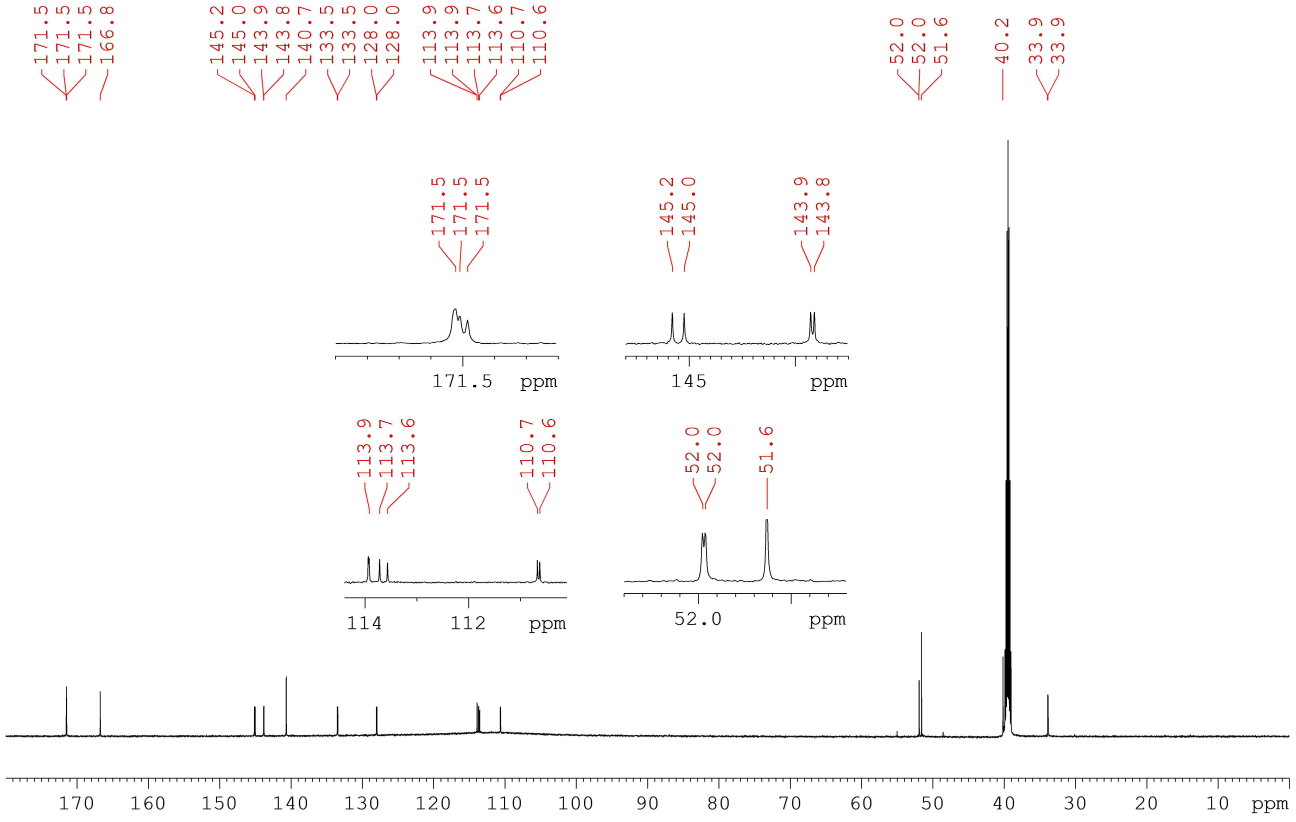

Figure S31. ^{13}C NMR (DMSO-d_6) spectrum of symphyocladins J/K **(9a/b)**

Table S8. 1D and 2D NMR data (600 MHz, DMSO-d_6) of symphyocladins J/K **(9a/b)**

pos	δ_H, mult (J in Hz)	δ_C	COSY	HMBC	ROESY
1		166.8			
2		133.5			
3	3.50, m, overlap	40.2	4a, 4b	1, 2, 4, 5, 6, 7′	
4a	2.98/2.97, dd (16.8, 10.8)	33.9	3, 4b	2, 3, 5, 6	7′
4b	2.41/2.40, dd (16.8, 3.0)		3, 4a	2, 3, 5, 6	7′
5[a]		171.5			
6[a]		171.5			
5-OCH_3[b]	3.52, s	52.0		5	
6-OCH_3[b]	3.541/3.53, s	51.6		6	
1′		128.07			
2′[c]		113.9			
3′[c]		113.7/113.6			
4′[d]		143.9/143.8			
5′[d]		145.2/145.0			
6′		110.7/110.6			
7′	7.39/7.38, s	140.7		1, 2, 3, 1′, 2′, 6′	4a, 4b

[a-d] assignments are interchangeable within the same letter.

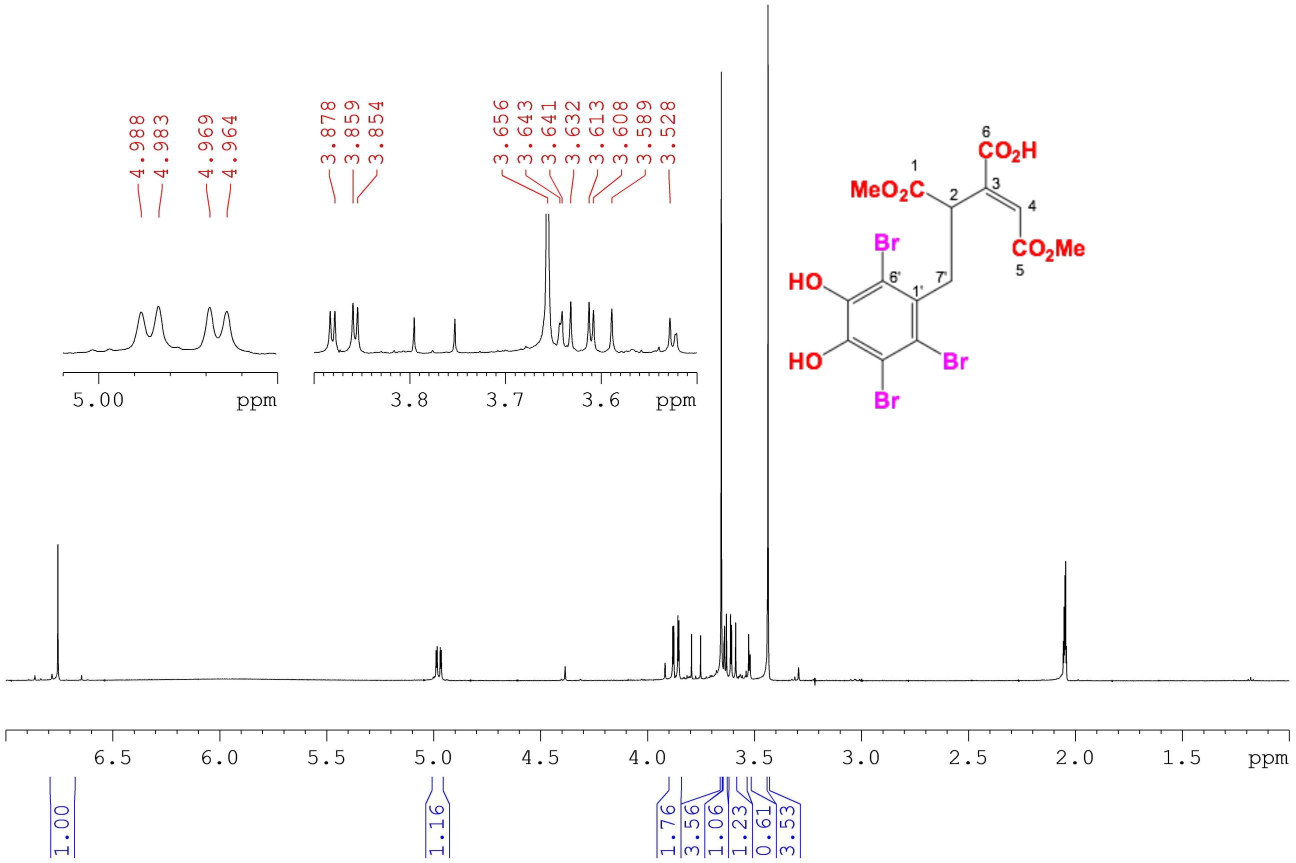

Figure S32. ^{1}H NMR (acetone-d_6) spectrum of symphyocladin L **(10)**

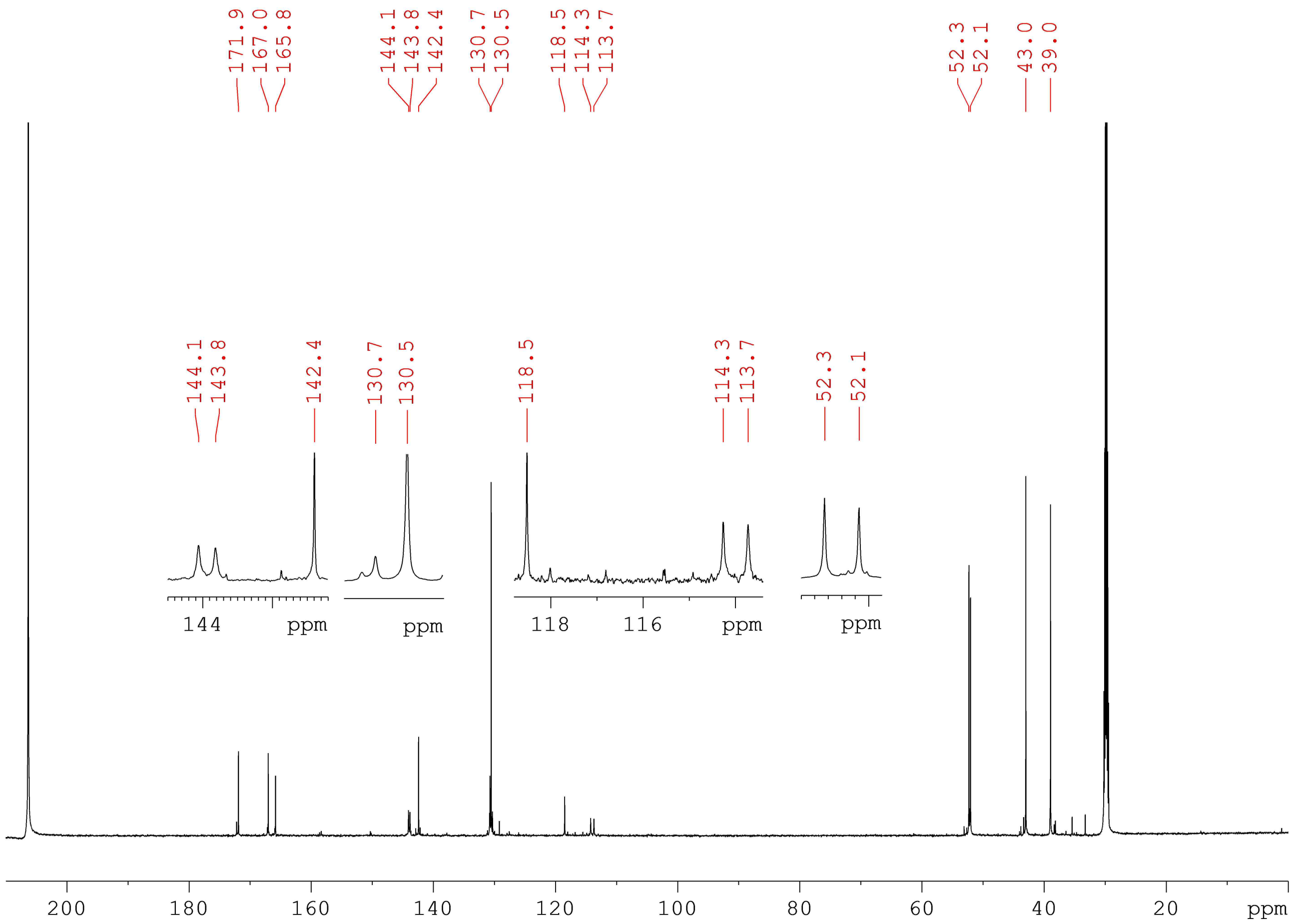

Figure S33. ^{13}C NMR (acetone-d_6) spectrum of symphyocladin L **(10)**

Table S9. 1D and 2D NMR data (600 MHz, acetone-d_6) of symphyocladin L **(10)**

pos	δ_H, mult (J in Hz)	δ_C	COSY	HMBC
1		171.9		
2	4.98, dd (11.4, 3.0)	43.0	7′a, 7′b	1, 3, 4, 6, 7′
3		142.4		
4	6.76, s	130.5		2, 3, 6
5		165.8		
6		167.0		
1-OCH_3	3.66, s	52.3		1
5-OCH_3	3.44, s	52.1		5
1′		130.7		
2′		118.5		
3′		113.7		
4′[a]		144.1		
5′[a]		143.8		
6′		114.3		
7′a	3.87, dd (14.4, 3.0)	39.0	2, 7′b	1, 2, 3, 1′, 2′, 6′
7′b	3.61, dd (14.4, 11.4)		2, 7′a	1, 2, 3, 1′, 2′, 6′

[a] assignments are interchangeable

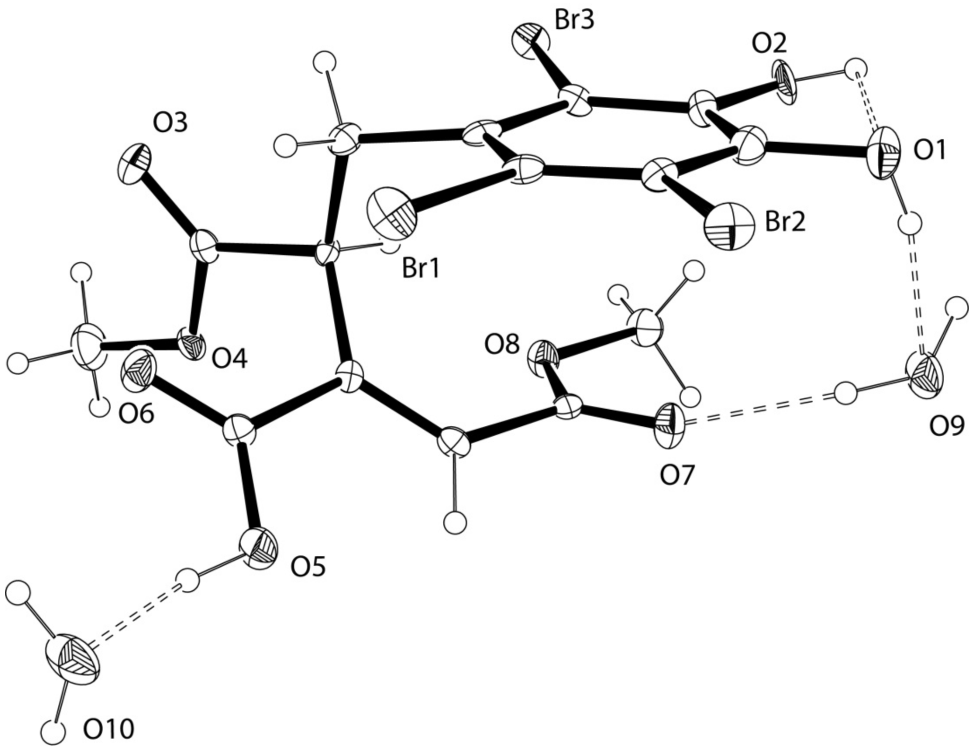

Figure S34. ORTEP view of symphyocladin L dihydrate (**10**) (30% probability ellipsoids shown).

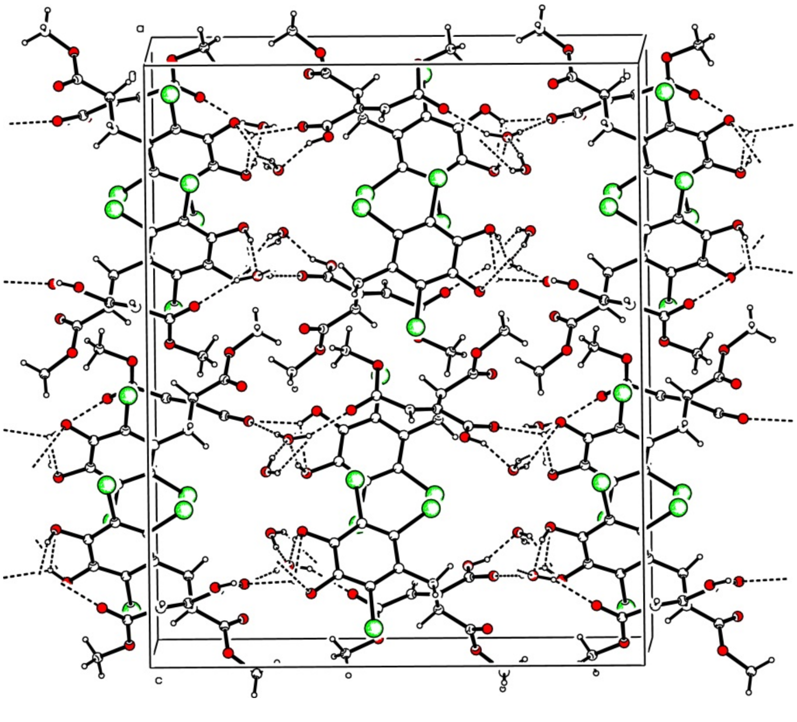

Figure S35. PLATON view of the unit cell of symphyocladin L dihydrate (**10**) showing H-bonding.

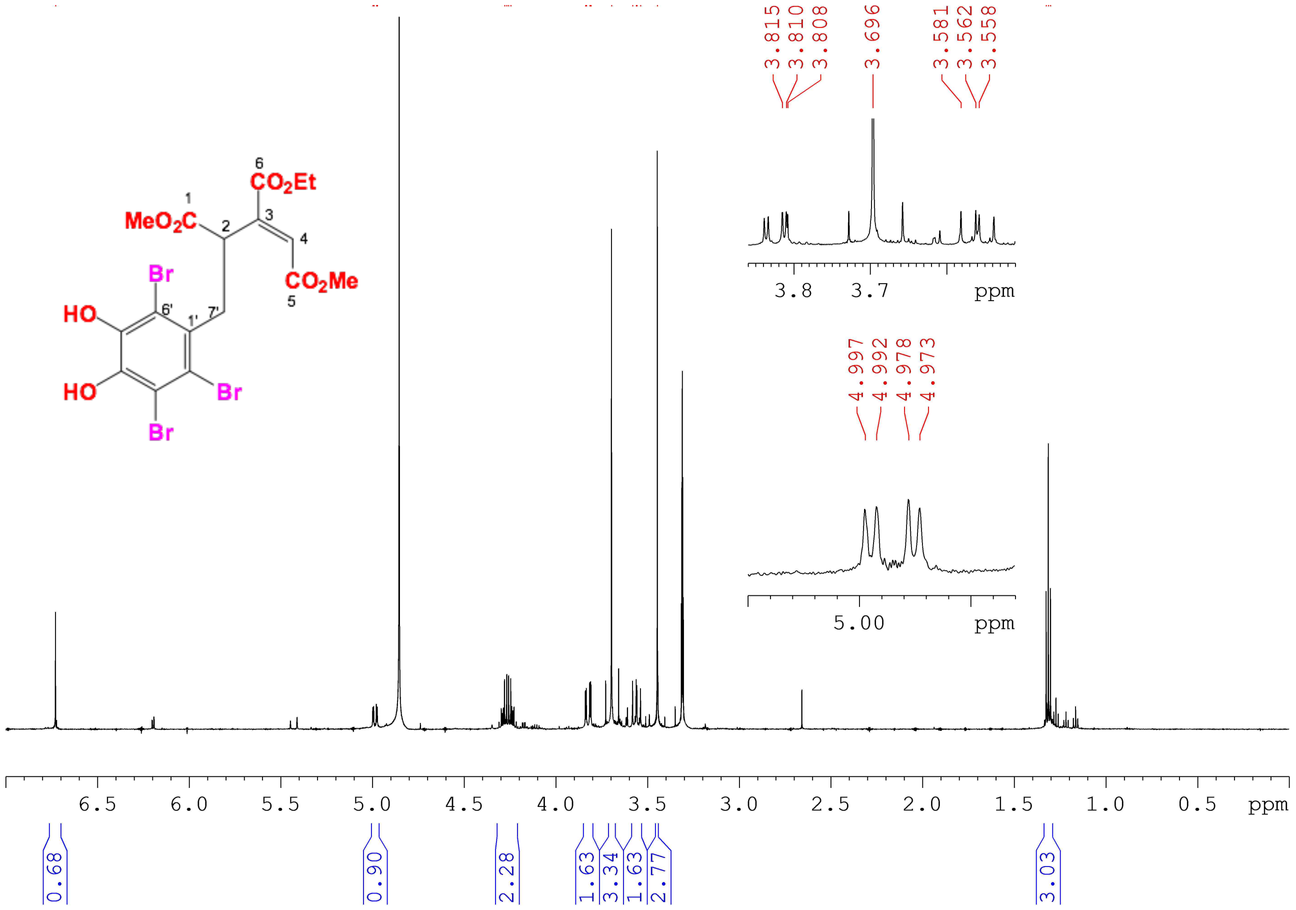

Figure S34. ^{1}H NMR (methanol-d_4) spectrum of symphyocladin M **(11)**

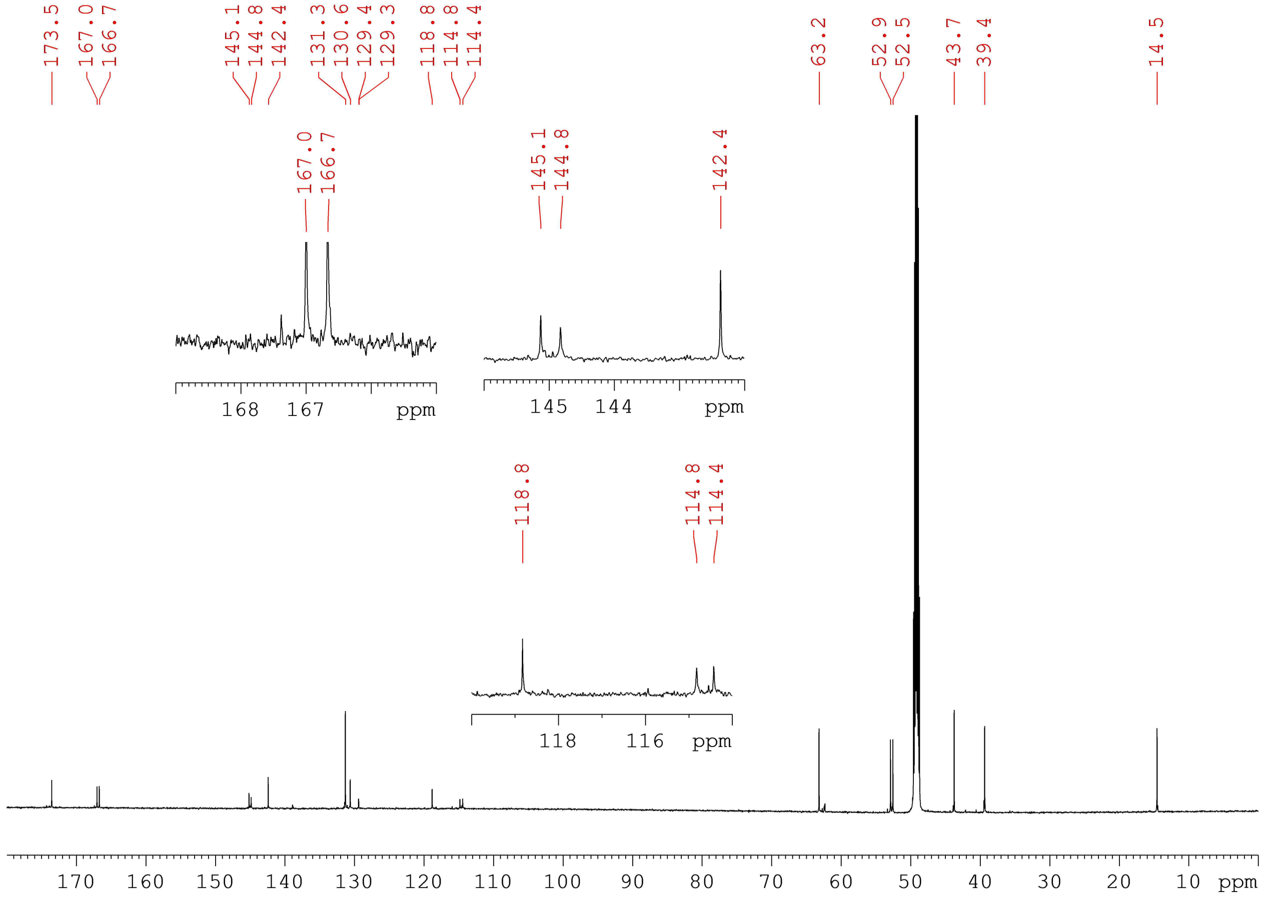

Figure S35. ^{13}C NMR (methanol-d_4) spectrum of symphyocladin M **(11)**

Table S10. 1D and 2D NMR data (600 MHz, methanol-d_4) of symphyocladin M **(11)**

pos	$\delta_{H,}$ mult (*J* in Hz)	δ_C	COSY	HMBC
1		173.5		
2	4.98, dd (11.4, 3.0)	43.7	7′a, 7′b	1, 3, 4, 6, 7′
3		142.4		
4	6.73, s	131.3		2, 5, 6
5		166.7		
6		167.0		
1-OCH_3	3.70, s	52.9		1
6-$OC\underline{H}_2CH_3$	4.26, br q (7.2)	63.2	8	6, 8
6-$OCH_2C\underline{H}_3$	1.31, t (7.2)	14.5	7	7
5-OCH_3	3.45, s	52.5		5
1′		130.6		
2′		118.8		
3′		114.4		
4′[a]		145.1[a]		
5′[a]		144.8[a]		
6′		114.8		
7′a	3.81, dd (14.4, 3.0)	39.4	2, 7′b	1, 2, 3, 1′, 2′, 6′
7′b	3.56, dd (14.4, 11.4)		2, 7′a	

[a] assignments are interchangeable;

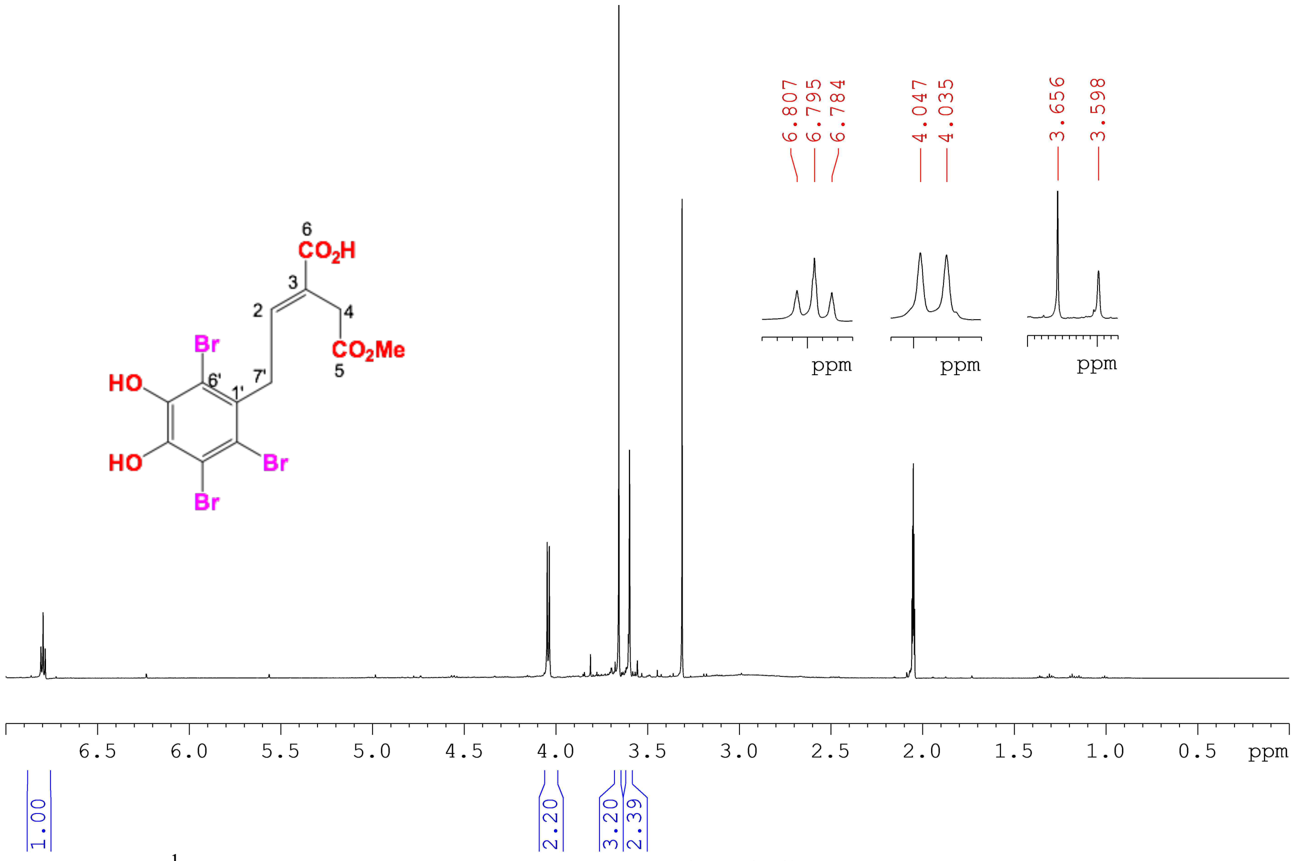

Figure S36. ^{1}H NMR (acetone-d_6) spectrum of symphyocladin N **(12)**

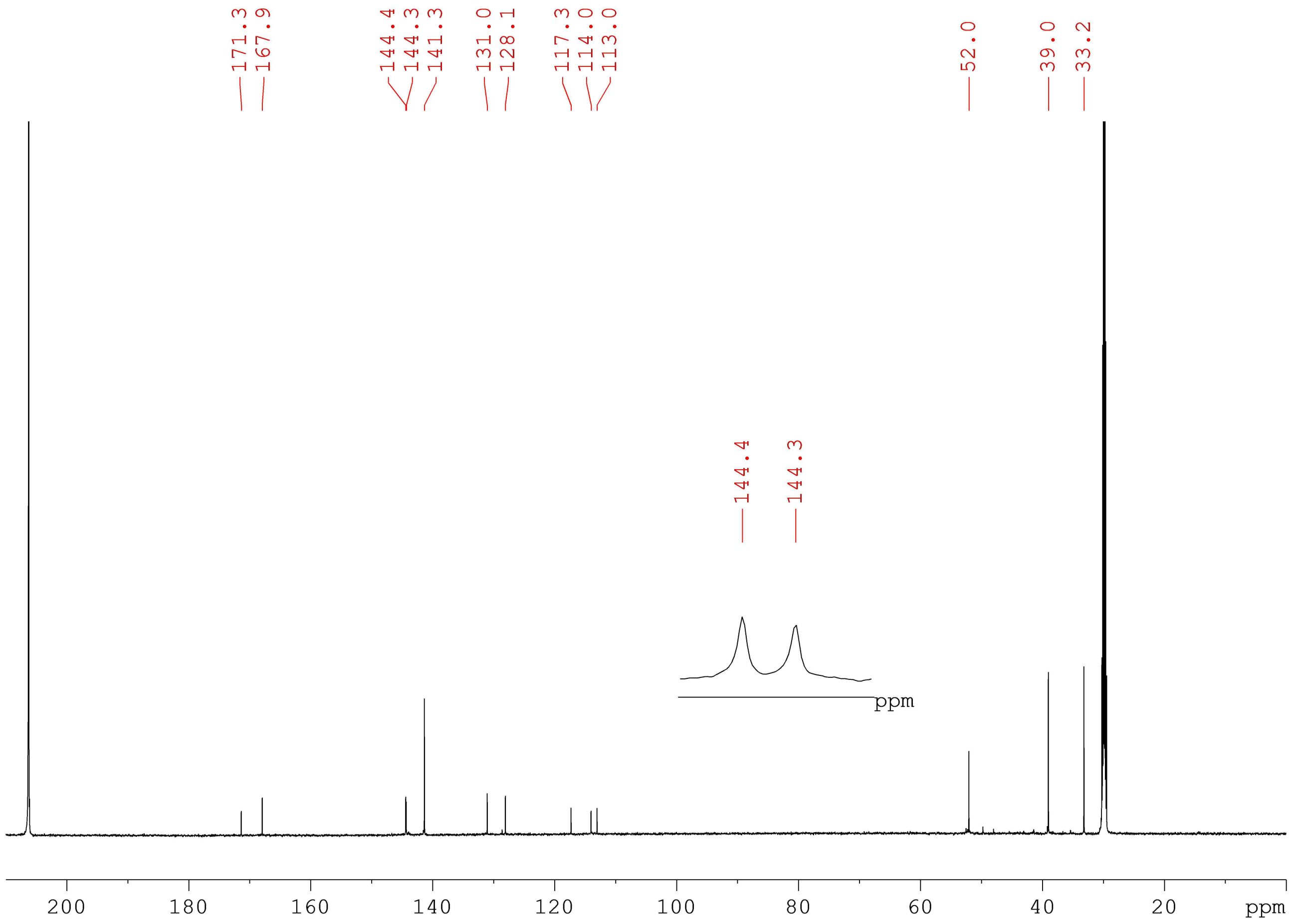

Figure S37. ^{13}C NMR (acetone-d_6) spectrum of symphyocladin N **(12)**

Table S11. 1D and 2D NMR data (600 MHz, acetone-d_6) of symphyocladin N **(12)**

pos	$\delta_{H,}$ mult (*J* in Hz)	δ_C	COSY	HMBC	ROESY
1					
2	6.79, brt (6.6)	141.3	7′	3, 4, 6, 1′, 7′	
3		128.1			
4	3.60, brs	33.2		2, 3, 5, 6,	7′
5		171.3			
6		167.9			
5-OCH_3	3.66, s	52.0		5	
1′		131.0			
2′		117.3			
3′		114.0			
4′[a]		144.4			
5′[a]		144.3			
6′		113.0			
7′	4.05, s	39.0	2	2, 3, 1′, 2′, 6′	4

[a] assignments are interchangeable

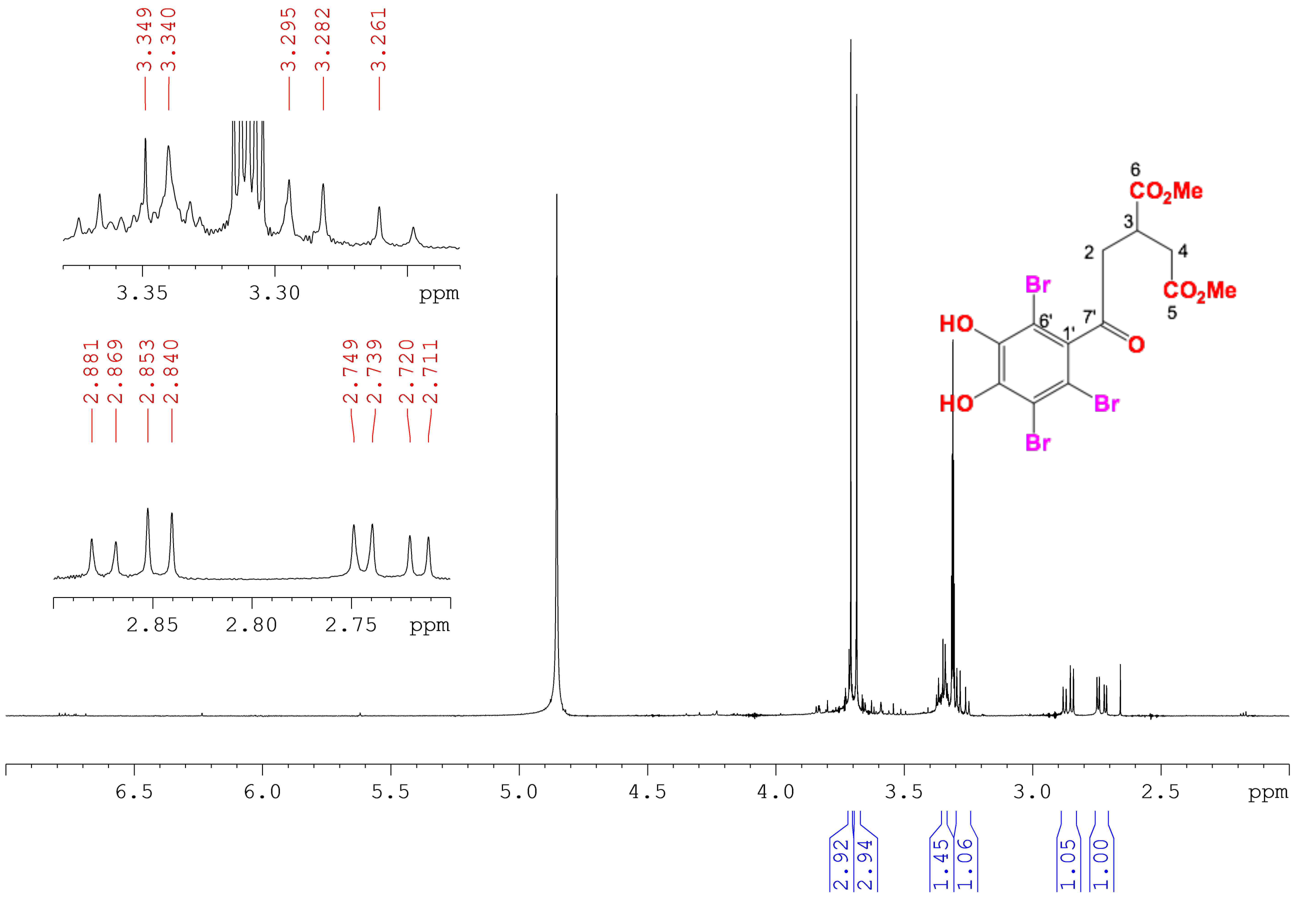

Figure S38. ^{1}H NMR (methanol-d_4) spectrum of symphyocladin O **(13)**

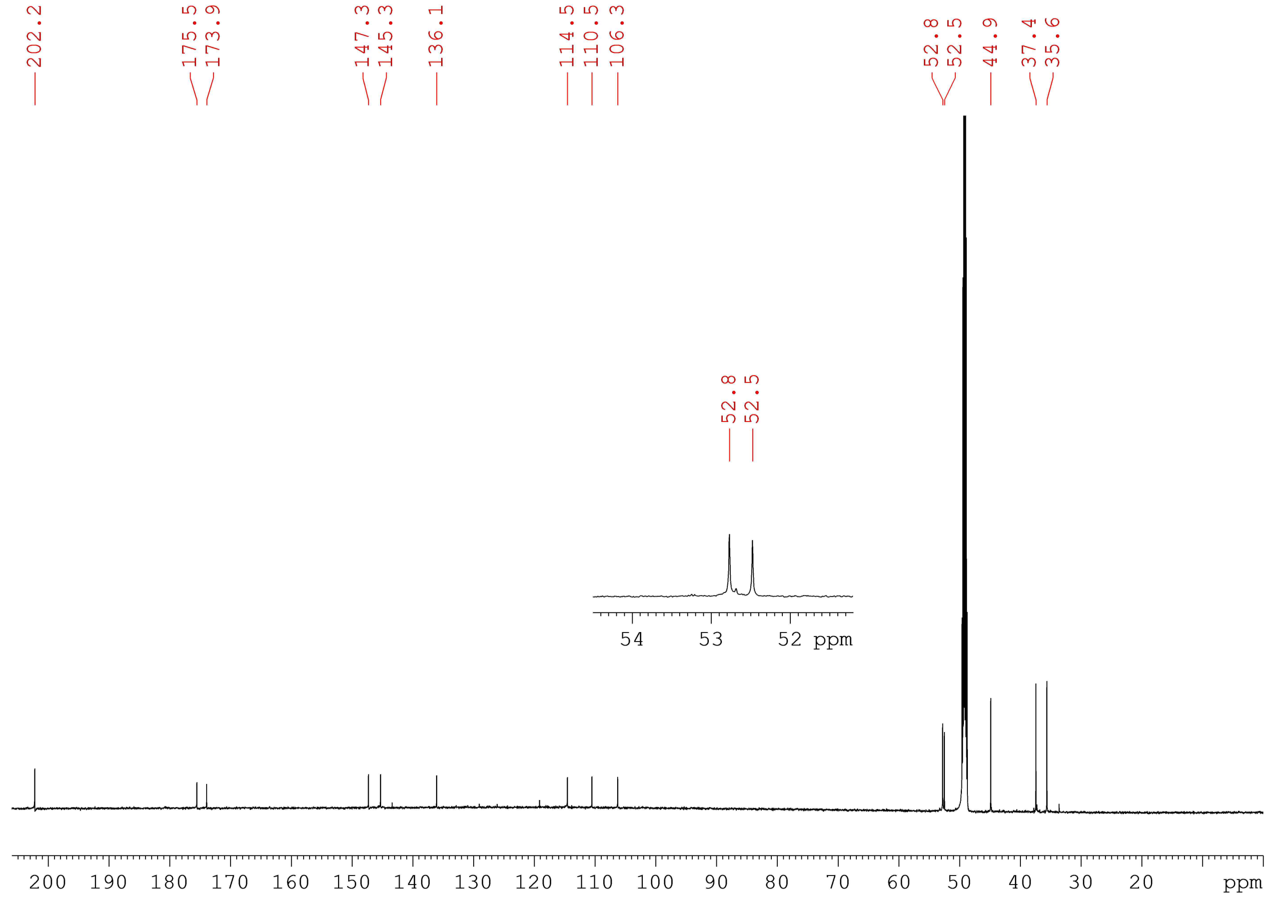

Figure S39. ^{13}C NMR (methanol-d_4) spectrum of symphyocladin O **(13)**

Table S12. 1D and 2D NMR data (600 MHz, methanol-d_4) of symphyocladin O **(13)**

pos	δ_H, mult (J in Hz)	δ_C	COSY	HMBC
2a	3.35, m, overlap	44.9	2b, 3	3, 4, 6, 7′
2b	3.27, dd (20.4, 7.8)		2a, 3	3, 4, 6, 7′
3	3.34, m, overlap	37.4	2a, 2b, 4a, 4b	2, 4, 5, 6, 7′
4a	2.86, dd (17.4, 7.2)	35.6	3, 4b	2, 3, 5, 6
4b	2.73, dd (17.4, 6.0)		3, 4a	2, 3, 5, 6
5		173.9		
6		175.5		
5-OCH_3	3.68, s	52.5		5
6-OCH_3	3.71, s	52.8		6
1′		136.1		
2′		114.5		
3′		110.5		
4′[a]		147.3		
5′[a]		145.3		
6′		106.3		
7′		202.2		

[a] assignments are interchangeable

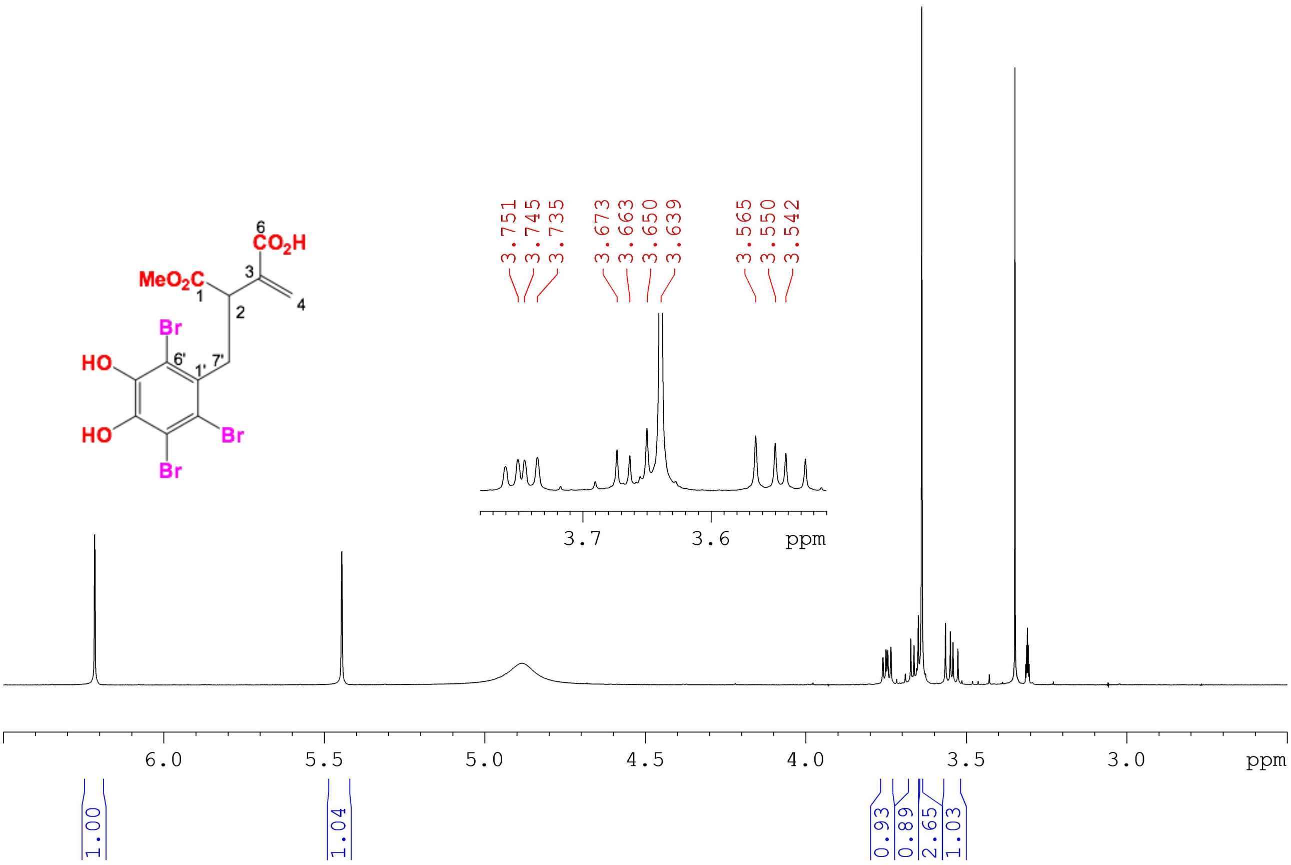

Figure S40. ^{1}H NMR (methanol-d_4) spectrum of symphyocladin P **(14)**

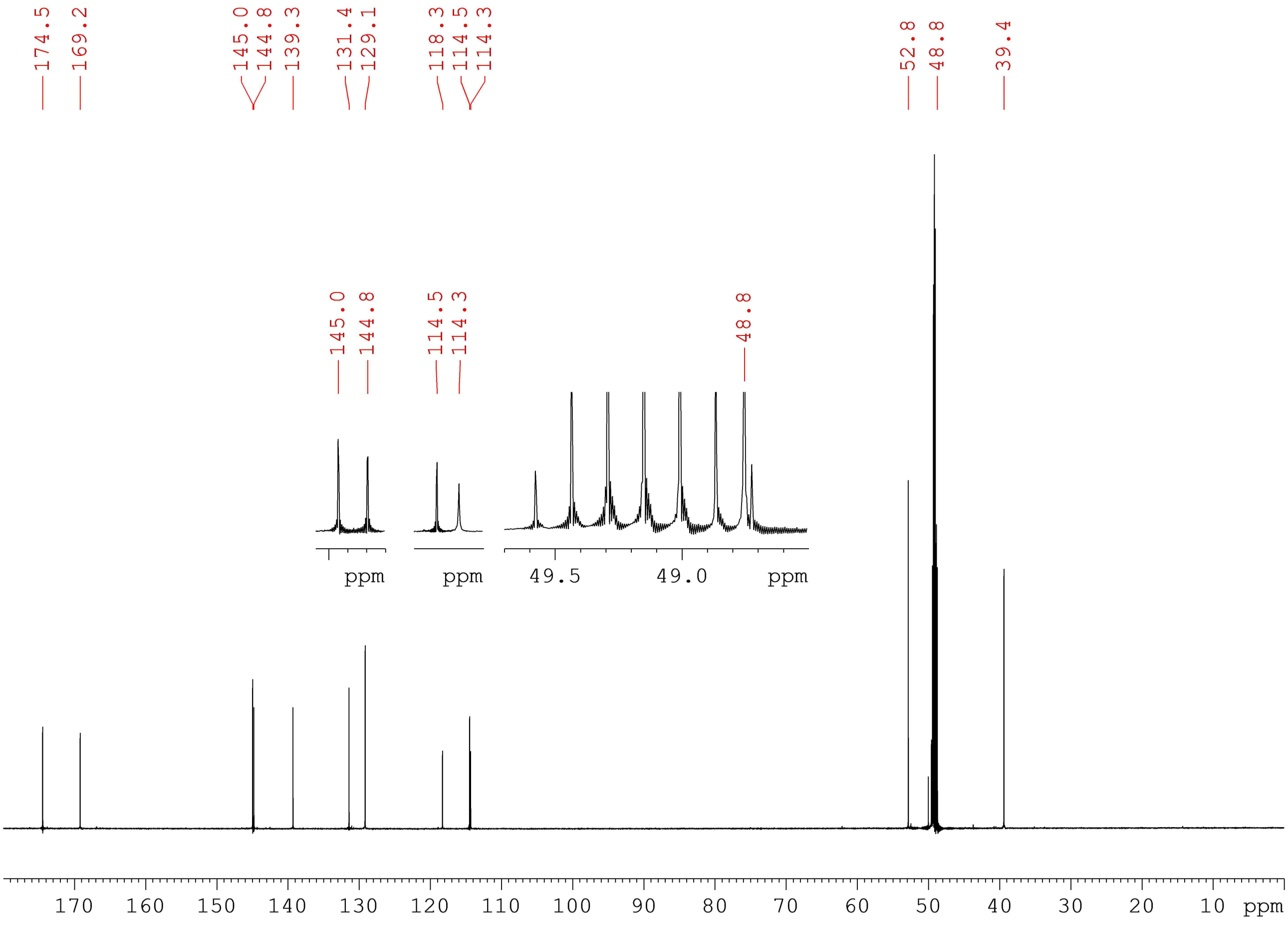

Figure S41. ^{13}C NMR (methanol-d_4) spectrum of symphyocladin P **(14)**

Table S13. 1D and 2D NMR (600 MHz, methanol-d_4) of symphyocladin P **(14)**

pos	$\delta_{H,}$ mult (J in Hz)	δ_C		HMBC	ROESY
1		174.5			
2	3.75, dd (9.0, 6.0)	48.8	7′a, 7′b	1, 3, 4, 6, 1′, 7′	4b
3		139.3			
4a	6.22, d (1.2)	129.1	4b	2, 3, 6	
4b	5.44, br s		4a	2, 3, 6	2
6		169.2			
1-OCH_3	3.64, s	52.8		1	
1′		131.4			
2′		118.3			
3′		114.5			
4′[a]		145.0			
5′[a]		144.8			
6′		114.3			
7′a	3.65, dd (13.8, 6.0)	39.4	2, 7′b	1, 2, 3, 1′, 2′, 6′	
7′b	3.54, dd (13.8, 9.0)		2, 7′a	1, 2, 3, 1′, 2′, 6′	

[a] assignments are interchangeable

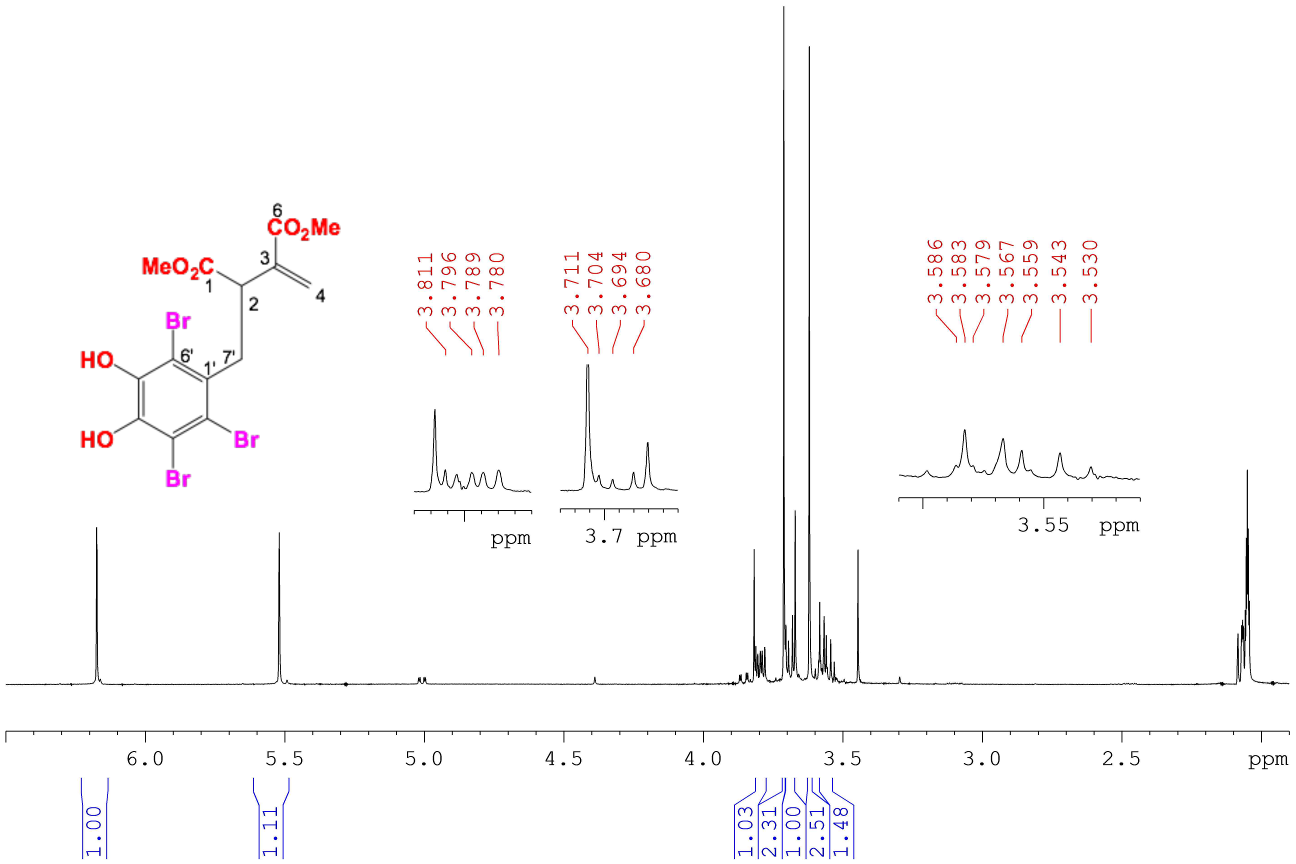

Figure S42. ^{1}H NMR (acetone-d_6) spectrum of symphyocladin Q **(15)**

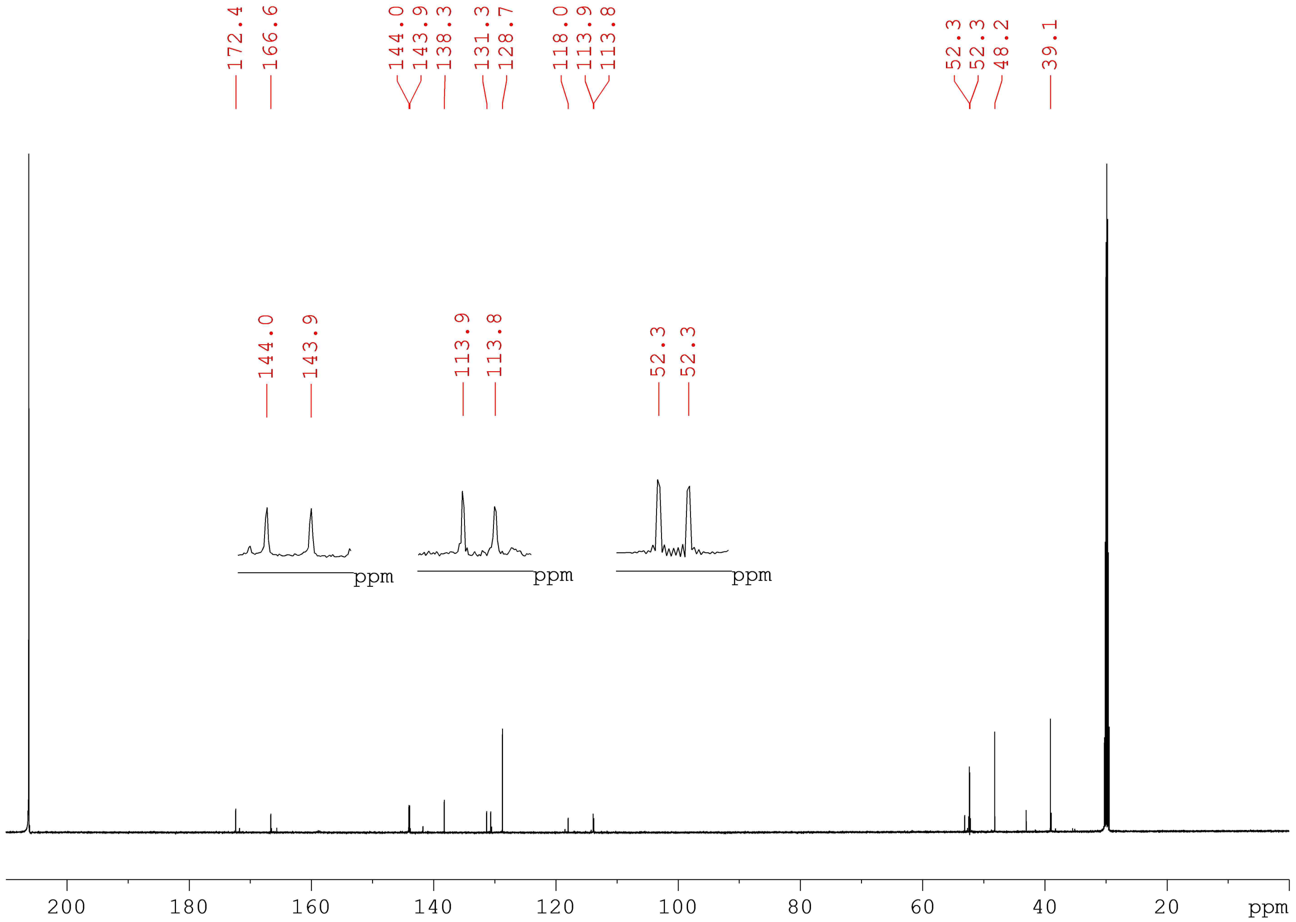

Figure S43. ^{13}C NMR (acetone-d_6) spectrum of symphyocladin Q **(15)**

Table S14. 1D and 2D NMR data (600 MHz, acetone-d_6) of symphyocladin Q **(15)**

pos	δ_H, mult (*J* in Hz)	δ_C	COSY	HMBC	ROESY
1		172.4			
2	3.79, dd (5.4, 9.6)	48.2	7′a, 7′b	1, 3, 4, 6, 1′, 7′	4b
3		138.3			
4a	6.17, d (1.2)	128.7	4b	2, 3, 6	
4b	5.52, s		4a	2, 3, 6	2
6		166.6			
1-OCH_3	3.62, s	52.3		1	
6-OCH_3	3.71, s	52.3		6	
1′		131.3			
2′		118.0			
3′		113.9			
4′[a]		144.0			
5′[a]		143.9			
6′		113.8			
7′a	3.69, dd (14.4, 5.4)	39.1	2, 7′b	1, 2, 3, 1′, 2′, 6′	
7′b	3.56, dd, (14.4, 9.6)		2, 7′a	1, 2, 3, 1′, 2′, 6′	

[a] assignments are interchangeable
